# Elevated temperatures diminish the effects of a highly resistant rice variety on the brown planthopper

**DOI:** 10.1038/s41598-020-80704-4

**Published:** 2021-01-08

**Authors:** Finbarr G. Horgan, Arriza Arida, Goli Ardestani, Maria Liberty P. Almazan

**Affiliations:** 1 EcoLaVerna Integral Restoration Ecology, Bridestown, Kildinan, Co. Cork Ireland; 2 Escuela de Agronomía, Facultad de Ciencias Agrarias Y Forestales, Universidad Católica del Maule, Casilla 7-D, Curicó, Chile; 3 Environment and Sustainable Resource Management, University College Dublin, Belfield, Dublin 4, Ireland; 4 International Rice Research Institute, DAPO Box 7777, Metro Manila, Philippines; 5 Department of Veterinary and Animal Sciences, University of Massachusetts, Amherst, MA 01003 USA

**Keywords:** Ecology, Climate sciences

## Abstract

This study compares the effects of temperature (constant at 15, 20, 25, 30 and 35 °C) on adult longevity, oviposition, and nymph development of the brown planthopper, *Nilaparvata lugens*, on susceptible and resistant rice varieties. The resistant variety contained the *BPH32* gene. In our experiments, nymphs failed to develop to adults at 15, 20 and 35 °C on either variety. Host resistance had its greatest effect in reducing adult survival at 20–25 °C and its greatest effect in reducing nymph weight gain at 25 °C. This corresponded with optimal temperatures for adult survival (20–25 °C) and nymph development (25–30 °C). At 25 and 30 °C, adult females achieved up to three oviposition cycles on the susceptible variety, but only one cycle on the resistant variety. Maximum egg-laying occurred at 30 °C due to larger numbers of egg batches produced during the first oviposition cycle on both the susceptible and resistant varieties, and larger batches during the second and third oviposition cycles on the susceptible variety; however, resistance had its greatest effect in reducing fecundity at 25 °C. This revealed a mismatch between the optimal temperatures for resistance and for egg production in immigrating females. Increasing global temperatures could reduce the effectiveness of anti-herbivore resistance in rice and other crops where such mismatches occur.

## Introduction

Global atmospheric CO_2_ concentrations surpassed 400 ppm in 2017, at that time representing the highest post-glaciation levels for at least 800 K years^1,2^. With continuously high CO_2_ emissions, global temperatures are predicted to rise by between 1.5 and 3 °C before 2100 (from 1850s levels^3^). Increasing global temperatures will affect insect functions, including herbivory of crop plants, at regional scales^4,5^. Rice is a predominantly tropical and subtropical crop. Rice is the major staple for people living in Asia and is produced on an estimated 108 M hectares in South and Southeast Asia alone^6^. Recent models suggest that, compared to other staple crops, rice is largely resilient to increasing temperatures and other global changes^7,8^. However, rice is vulnerable to a range of insect herbivores, some of which exhibit large-scale responses to weather conditions. Furthermore, gradual changes in the life-histories and distributions of a number of rice herbivores have been correlated with global climate anomalies^9–11^. For example, researchers have documented a northward shift in distribution of the green shield bug, *Nezara viridula*, in Japan that may be related to warmer winter temperatures^12^. Furthermore, planthoppers and leaffolders exhibit outbreak dynamics related to extreme weather events, and some research suggests that recent increases in pest population densities could be associated with increasingly warm local weather conditions^9,10^. Despite such observations of direct climate effects on rice pest populations, it is the interactions between climate and rice crop management that are likely to have the greatest effects on rice herbivores. For example, high fertilizer rates and the misuse or overuse of pesticides destabilize pest populations^13^, which could potentially exacerbate climate related outbreaks. In contrast, crop management practices that promote pest population stability will increase crop resilience against pests under changing and more variable climates^14–16^.

Among possible stabilizing management practices, host plant resistance has received sustained public research attention for the last several decades^13,17^. A large focus on rice resistance to the brown planthopper, *Nilaparvata lugens*, has been largely due to the destructive potential of the species and to successes in identifying gene-for-gene resistance mechanisms^17^. To date, over 40 genes for resistance to planthoppers have been identified, and many have been introgressed into high-yielding rice varieties using marker-assisted selection^17^. However, the selection of virulent planthopper populations, capable of feeding on resistant varieties, is often rapid (e.g., 10 to 15 generations in tropical Asia) and, consequently, the availability of broad spectrum and durable resistant varieties can become limited^13^. Despite these trends, the rice variety IR62 has maintained its resistance for over 30 years^18^. In screening trials, the variety effectively reduced the fitness of planthoppers at multiple sites in South and Southeast Asia^18^. Resistance in IR62 is associated with the *BPH32* gene derived from PTB33^19,20^. The *BPH32* gene encodes a protein with a signal peptide and SCR-domain that likely binds to planthopper glycoproteins or tissues to inhibit feeding^20^. Selection studies have further shown that planthoppers adapted to feed on IR62 will gain virulence against the resistant variety Rathu Heenati^21,22^. Such virulence-adapted planthoppers often possess relatively high densities of yeast-like symbionts (YLS) compared to non-virulent planthoppers feeding on susceptible varieties, which may compensate for the effects of anti-feedants^21,23^. Yeast-like symbionts are essential for planthopper nutrition and egg development^24–26^. Resistance in Rathu Heenati has been attributed to a number of genes that also include *BPH32*^27^. Rathu Heenati and derived rice lines have high flavonoid concentrations that function as planthopper anti-feedants^28,29^. Meanwhile, volatiles from Rathu Heenati and PTB33 increase planthopper adult and nymph mortality and decrease adult settling and feeding^30^. Because of the spectrum and durability of varieties with the *BPH32* gene, PTB33, Rathu Heenati and related lines have become popular resistance donors in rice breeding programs that target planthopper hotspots^20,27,29,31,32^.

The fitness (survival × reproduction) of insect herbivores is known to vary on resistant plants under the influence of ambient temperatures. This may include reductions in the effectiveness of host resistance at low^33,34^ and high temperatures^33–35^. Such losses of resistance can result from temperature-related decreases in the production of defensive compounds, decreases in the effectiveness of those compounds at sub-optimal temperatures^36^, or because the stimulatory effects of temperature on herbivore fitness overwhelm the plant’s defenses^35^. Despite the rapid progress in identifying novel resistance genes against the brown planthopper and in elucidating related resistance mechanisms, surprisingly few studies have examined the stability of rice resistance against planthoppers in the context of a changing global climate. Indeed, only a few studies have systematically assessed the responses of planthoppers to ambient temperatures as mediated through the rice host, even on susceptible varieties^37^. Furthermore, studies that have examined temperature effects on rice resistance to planthoppers (including the whitebacked planthopper, *Sogatella furcifera*) have mainly applied only standard screening protocols^34,38–40^ that confound resistance and tolerance, or have focused on varieties with genes that are currently ineffective throughout Asia^33,34^. Therefore, it is still largely unknown how planthopper fitness and population parameters will be differentially affected by the combined and separate effects of host resistance and elevated temperatures as the global climate continues to warm. Factorial experiments that include at least two levels of host plant resistance across gradients of temperature are required to better elucidate such potential effects.

In the present study, we document the effects of temperature on planthopper life-history, and examine the interactions between temperature and rice resistance against the brown planthopper on IR62. Because resistance in IR62 is largely attributed to anti-feeding^20,28,41^, we predicted that optimal temperatures for resistance would coincide with optimal temperatures for nymph growth and development. As a corollary, we predicted that resistance would contribute relatively less to fitness reductions at sub-optimal temperatures because of slower development at low temperatures and physiological stresses, including a loss of symbionts, at higher temperatures. We further assessed the stability of resistance against ovipositing female planthoppers at optimal and sub-optimal temperatures and examined patterns of egg-laying by planthoppers on IR62 and on the susceptible variety, IR22, to elucidate factors contributing to fecundity at different temperatures. To our knowledge, this is the first study to detail daily patterns in egg-laying by the brown planthopper across a gradient of temperatures and to describe temperature effects on ovipostion cycles. The study is also the first to examine brown planthopper resistance using a full-factorial design that includes a temperature gradient. We discuss our results in terms of developing climate resilient crop production systems for Asia.

## Results

### Adult survival and oviposition

**Figure 1:**
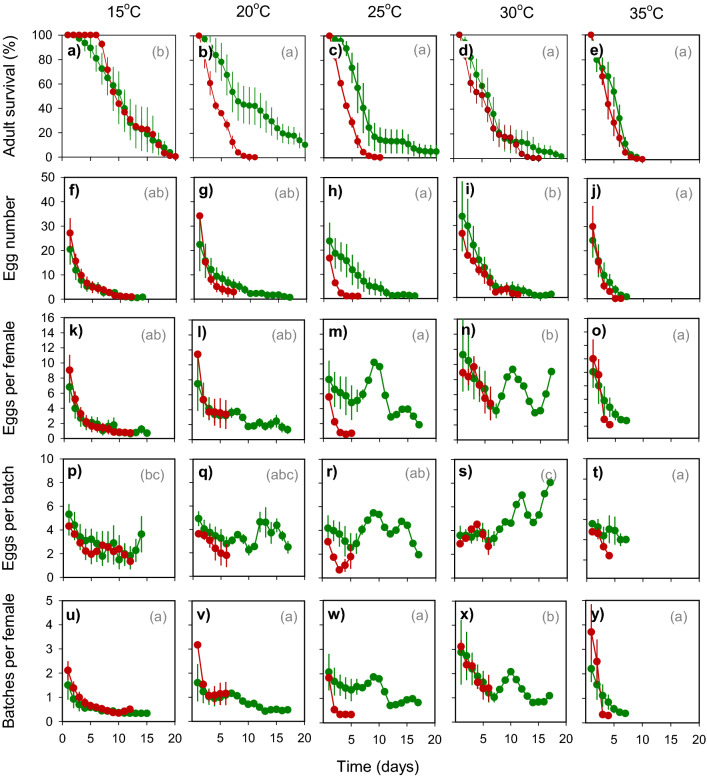
Daily survival and egg-laying by *Nilaparvata lugens* on susceptible (IR22: green circles) and resistant (IR62: red circles) rice varieties. Plants were grown at 15 °C (**a,f,k,p,u**), 20 °C (**b,g,l,q,v**), 25 °C (**c,h,m,r,w**), 30 °C (**d,i,n,s,x**) and 35 °C (**e,j,o,t,y**). Relative humidity was maintained at 80%. Results for Adult longevity (**a–e**) and the total number of eggs deposited (**f–j**) are indicated. The number of eggs per surviving female (**k–o**), the size of egg batches (**p–t**) and the number of batches produced per female (**u–y**) are indicated as components of egg-laying displaying three oviposition cycles on IR22 between 15 and 30 °C. Standard errors are included (N = 4). Lowercase letters indicate homogenous temperature groups for each parameter (Tukey: *P* ≤ 0.05).

**Figure 2:**
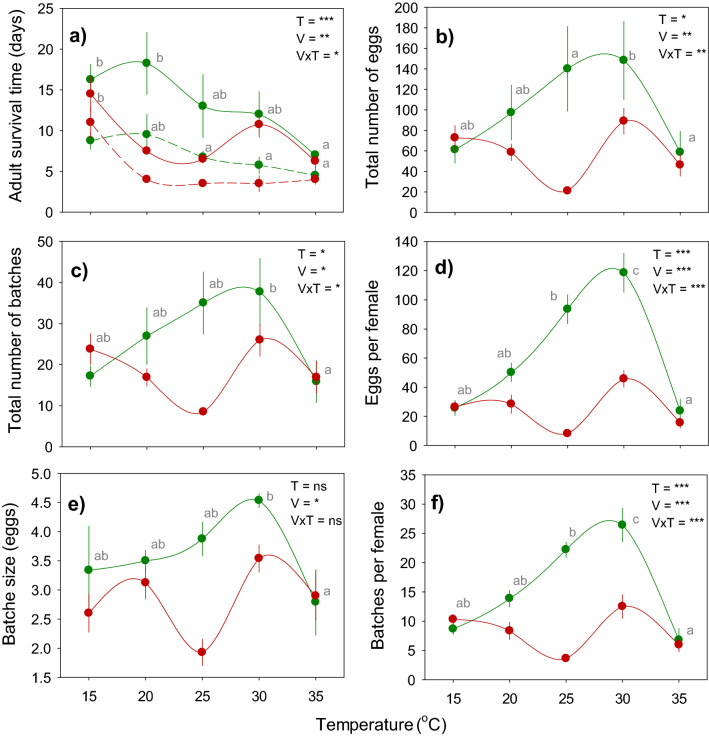
Mean survival and egg-laying by *Nilaparvata lugens* on susceptible (IR22: green circles) and resistant (IR62: red circles) rice varieties across a range of temperatures. The times to 50% (dashed lines) and 100% (solid lines) mortality of adult females are indicated in **a**. The total numbers of eggs (**b**), total number of batches (**c**), the number of eggs per female (**d**), batch size (**e**) and the numbers of batches per female (**f**) are also indicated. Numbers are based on accumulated data over 20 days of the experiment. Relative humidity was maintained at 80%. Bars indicate standard errors (N = 4). Effects of variety (V), temperature (T) and their interaction (V × T) are indicated as ns (*P* > 0.05), * (*P* ≤ 0.05), ** (*P* ≤ 0.01), *** (*P* ≤ 0.005); lowercase letters indicate homogenous temperature groups (Tukey, *P* ≤ 0.05).

We examined survival and egg-laying in adult females on the susceptible rice variety IR22, and on the resistant variety IR62 at 15, 20, 25, 30 and 35 °C. In particular, we examined cyclic oviposition responses to the varieties and temperatures. Adult survival declined over the course of the experiment with significant two and three-way interactions due to differences in the rates of decline at different temperatures and for the two varieties (Table 1). Longevity was lower on IR62 and was greatest at 15 °C, but not significantly different for temperatures ≥ 25 °C (Fig. 1a–e). A significant three-way interaction was due to similar patterns in longevity on both hosts at 15 °C, but reduced longevity on the resistant host at 20–30 °C. Whereas total female longevity (time to 100% mortality) peaked at 15 °C, the time to 50% mortality peaked at 20 °C (Fig. 2a). Furthermore, total longevity on IR62 showed a clear increase at 30 °C due to a comparatively extended longevity among late survivors on the variety.

**Table 1:** F-values from repeated measures GLM for adult female longevity and oviposition parameters (see Fig. 1).

Source of variation^1^	Df^2^	F-values^3^					
		Adult longevity	Total eggs laid	Total batches produced	Eggs per surviving female	Batch size	Batches per surviving female
**Within-subject effects**							
Time	5 (3)	84.769***	46.109***	44.473***	36.957***	22.265***	28.964***
Time*variety	5 (3)	9.349***	4.698***	6.779***	4.795***	2.424 ns	5.127**
Time*temperature	20 (12)	5.013***	2.695***	1.767*	4.908***	6.841***	1.792 ns
Time*run	15 (9)	1.161 ns	1.884*	2.293**	1.612 ns	1.284 ns	1.702 ns
Time*variety * temperature	20 (12)	2.666***	1.264 ns	1.216 ns	2.493**	1.911*	1.674 ns
Error	135 (81)						
**Between-subject effects**							
Variety	1	17.292***	5.423*	2.875 ns	2.142 ns	8.953**	0.140 ns
Temperature	4	10.792***	3.93**	3.324*	3.875**	5.265***	5.244***
Run	3	1.916 ns	1.075 ns	0.845 ns	0.320 ns	0.244 ns	0.288 ns
Variety*temperature	4	3.980 ns	2.244 ns	3.399*	2.878*	3.696*	1.473 ns
Error	27						

^1^Time = time in days as experiment progresses; run = temporal replicate that includes observations for all temperatures conducted across different climate chambers.

^2^Degrees of freedom in parentheses are for batches, eggs and batch size per female (i.e., last three columns).

^3^ ns = *P* > 0.05, *P ≤ 0.05, **P ≤ 0.01, ***P ≤ 0.001.

The number of egg batches per female, the size of the egg batches, and the total numbers of batches and of eggs laid varied throughout the course of the experiment (Table 1; Fig. 1). Egg-laying on both IR22 and IR62 was highest at 30 °C (Fig. 2; Table 2). Individual females on IR22 displayed clear oviposition cycles in the numbers of batches produced and the size of batches. At 25 and 30 °C, peaks were observed at about 1, 9 and 14–15 days after the initiation of experiments, representing 1st, 2nd and 3rd oviposition cycles, respectively (Fig. 1m–n). At 30 °C, batch sizes increased over successive cycles on IR22 (Fig. 1s). On the resistant variety IR62, adults laid eggs during only a single, initial cycle (Fig. 1k–o). The larger batches (Fig. 1p–t) and higher numbers of batches (Fig. 1u–y) per planthopper at 30 °C and on IR22 resulted in significant [time × temperature] and [time × variety] interactions (Table 1). However, over the course of the experiment, the lowest numbers of eggs were produced at 25 and 35 °C because of effective resistance at 25 °C and lower survival at 35 °C (Fig. 2; Table 2).

**Table 2:** F-values from univariate GLMs for adult female longevity and oviposition at the end of 20 days (see Fig. 2).

Source of variation^1^	Df	F-values^2^						
		Time to 50% mortality	Time to 100% mortality	Total number of eggs	Total number of batches	Number of eggs per female	Number of batches per female	Batch size
Variety	1	6.334**	11.081***	7.482**	4.292*	50.683***	5.966*	51.454***
Temperature	4	6.607***	5.440***	3.441*	2.966*	15.877***	3.496 ns	17.648***
Run	3	0.343 ns	3.145*	1.392 ns	1.476 ns	0.333 ns	0.430 ns	0.301 ns
Variety *temperature	4	3.167*	2.349 ns	3.061*	3.358*	12.186***	1.988 ns	13.74***
Error	27							

^1^Run = temporal replicate that includes observations for all temperatures conducted across different climate chambers.

^2^ ns = *P* > 0.05, *P ≤ 0.05, **P ≤ 0.01, ***P ≤ 0.001.

### Nymph survival and development

**Figure 3:**
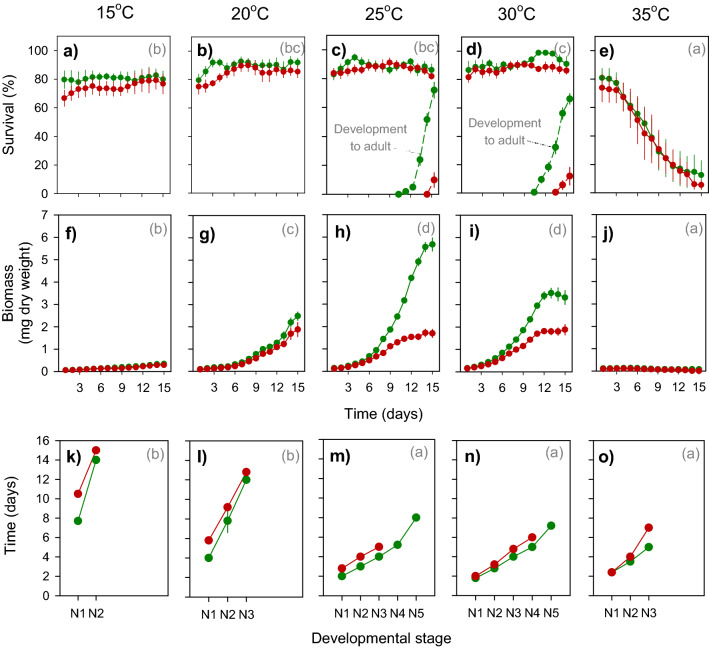
Daily survival and biomass of *Nilaparvata lugens* nymphs on susceptible (IR22: green circles) and resistant (IR62: red circles) rice varieties, with associated nymphs development stages. Plants were grown at 15 °C (**a,f,k**), 20 °C (**b,g,l**), 25 °C (**c,h,m**), 30 °C (**d,i,n**) and 35 °C (**e,j,o**). Relative humidity was maintained at 80%. Results for nymph survival, including development to adults (**a-e**), nymph biomass (**f-j**), and nymph developmental stages (**k–o**) are indicated. N2 = second instar, N3 = third instar, etc., A = adult. Standard errors are included (N = 5). Lowercase letters indicate homogenous temperature groups (Tukey, *P* ≤ 0.05).

**Figure 4:**
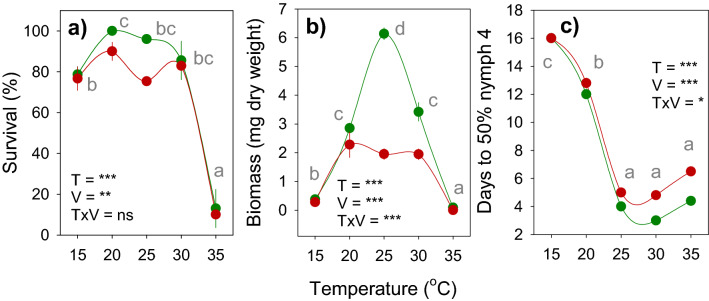
Mean survival, biomass and development of *Nilaparvata lugens* nymphs on susceptible (IR22: green circles) and resistant (IR62: red circles) rice varieties across a range of temperatures. Nymph survival at the end of 15 days is indicated in **a**. The biomass of surviving nymphs (**b**) and the time for 50% of nymphs to develop to fourth instars (**c**) are also indicated. Numbers are based on accumulated data over 15 days of the experiment (23 and 30 days for nymph development at 20 °C and 15 °C, respectively). Relative humidity was maintained at 80%. Bars indicate standard errors (N = 5). Effects of variety (V), temperature (T) and their interaction (V × T) are indicated as ns (*P* > 0.05), * (*P* ≤ 0.05), ** (*P* ≤ 0.01), *** (*P* ≤ 0.005); lowercase letters indicate homogenous temperature groups (Tukey, *P* ≤ 0.05).

We monitored nymph survival and development on IR22 and IR62 at 15, 20, 25, 30 and 35 °C. Survival, nymph dry weights and development stages were recorded. Nymph survival was relatively constant between 15 and 30 °C, but declined rapidly over the course of 15 days at 35 °C (Table 3; Fig. 3a–e). This produced significant time and temperature effects and a significant [time × temperature] interaction (Table 3). Host variety had no significant effect on nymph survival over the course of the experiment (Table 3); however, fewer nymphs had survived on IR62 by the end of the experiment (Temperature: F_4,36_ = 91.632, *P* < 0.001; Variety: F_1,36_ = 7.634, *P* = 0.009; Interaction: F_4,36_ = 0.928, *P* = 0.459; Fig. 4a).

**Table 3:** Results (F-values) of repeated measures GLMs for nymph survival and biomass over 15 days with results (F-values) of multivariate GLM for nymph development stages (nymphs 2 to 4)(see Fig. 3).

Source of variation^1^	Df	F-values^2^				
		Nymph survival	Nymph biomass	Nymph development		
				N1	N2	N3
**Within-subject effects**						
Time	14	8.143***	675.566***	-	-	-
Time*variety	14	1.626 ns	33.600***	-	-	-
Time*temperature	56	13.494***	109.702***	-	-	-
Time*run	56	1.568**	1.504**	-	-	-
Time*variety*temperature	56	1.157 ns	10.834***	-	-	-
Error	504					
**Between-subject effects**						
Variety	1	2.958 ns	141.268***	128.647***	7.673**	40.070***
Temperature	4	50.382***	557.817***	789.971***	327.969***	2321.609***
Run	4	2.289 ns	2.368 ns	1.588 ns	1.619 ns	0.391 ns
Variety*temperature	4	0.078 ns	27.685***	32.471***	1.367 ns	2.896*
Error	36					

^1^Time = time in days as experiment progresses; run = temporal replicate that includes observations for all temperatures conducted across different climate chambers.

^2^ ns = *P* > 0.05, * = *P* ≤ 0.05, **P ≤ 0.01, ***P ≤ 0.001.

**Figure 5:**
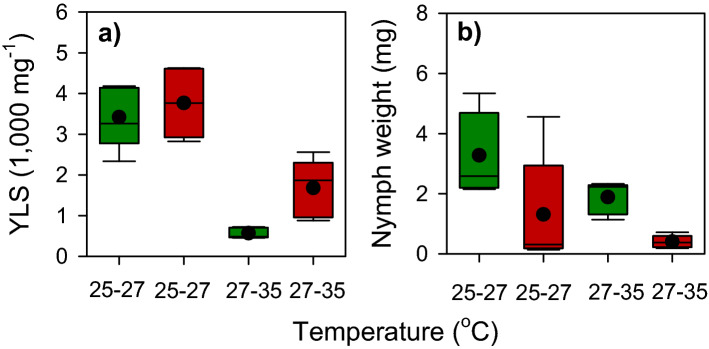
(**a**) Densities of yeast-like symbionts (YLS) in planthoppers reared on IR22 (green squares) and IR62 (red squares). Nymphs were maintained in an insectary at 27 °C for 7 days after exposure to 25 °C or 35 °C during 3 days. The corresponding wet weights of the nymphs are indicated in **b**. Black circles indicate sample means (N = 5).

Nymphs gained biomass over the course of the experiment (Table 3; Fig. 3f–j). This was affected by temperature and host variety with the greatest biomass achieved at 25 and 30 °C (Table 3). All two-way and three-way interactions were significant because of similar weight gains at 15 and 35 °C (irrespective of variety) and similar biomass during early parts of the experiment on both varieties, but a later divergence to produce smaller nymphs on IR62 (Table 3). Biomass at the end of the experiment was greatest for planthoppers reared at 25 °C and on IR22 (Temperature: F_4,36_ = 193.612, *P* < 0.001; Variety: F_1,36_ = 57.724, *P* < 0.001; Interaction: F_4,36_ = 12.754, *P* < 0.001; Fig. 4b). We assessed YLS densities in planthopper nymphs exposed to 25 °C and 35 °C for 3 days and maintained at 27 °C for a further 7 days. Nymphs feeding on IR62 had higher YLS densities (F_1,16_ = 5.874, *P* = 0.028) under both temperature regimes. After exposure to 35 °C, YLS densities declined by ca 56% and 84% in planthoppers feeding on IR62 and IR22, respectively (F_1,16_ = 67.296, *P* < 0.001). Despite differences in the proportional losses in symbionts on the two varieties, nymphs on IR62 showed a greater loss in body weight (70%) compared to nymphs on IR22 (43%) (F_1,16_ = 10.289, *P* = 0.005) and at 35 °C (F_1,16_ = 4.559, *P* = 0.049) (Fig. 5).

The times for nymphs to develop to second (N1), third (N2) and fourth (N3) instars were affected by temperature and host variety (Table 3). Development was slower at 15 and 20 °C, but rates were similar at higher temperatures (Fig. 3k–o). Development to fourth instar was between 1 day (> 20 °C) and 3 days (15 °C) slower on IR62 (Table 3; Fig. 4c). There was a significant [temperature × variety] interaction for development time to second (N2) instar because of similar rates on both varieties at above 30 °C, but slower rates on the resistant variety at between 15 and 25 °C (Table 3; Fig. 3k–o). Interactions were not significant for the other nymph stages. Adults emerged only at 25 and 30 °C (Table 3; Fig. 3h,i). Survivors were monitored at 15 °C until 30 days and at 20 °C until 23 days without any adults emerging. The proportion of surviving nymphs emerging as adults was similar between 25 and 30 °C (F_1,12_ = 3.175, *P* = 0.100), but fewer adults emerged on IR62 (F_1,12_ = 38.485, *P* < 0.01; Fig. 3h,i).

### Stability of resistance

**Figure 6:**
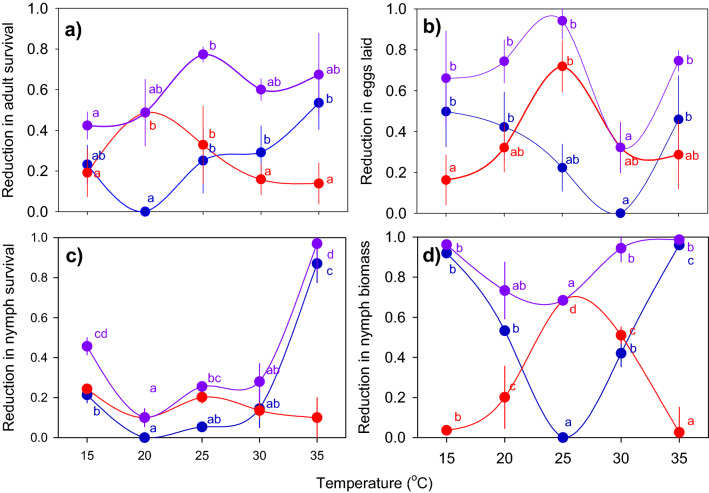
Reductions in the fitness of *Nilaparvata lugens* on IR62 over a temperature gradient. Graphs indicate the total reductions (violet circles) in adult longevity (**a**), the number of eggs laid (**b**), nymph survival (**c**) and nymph biomass (**d**). In each case, this is composed of a fitness reductions due to temperature (blue circles – estimated based on reductions relative to optimal temperatures on a highly susceptible variety) and reductions due to the resistance gene(s) (red circles – estimated as the total reduction minus the temperature-related reduction in fitness). Standard errors are shown (N = 4 for **a** and **b**, N = 5 for **c** and **d**). Lowercase letters indicate homogenous temperature groups (Tukey, *P* < 0.05).

We estimated the separate effects of temperature and host resistance on planthopper fitness by measuring fitness reductions at sub-optimal temperatures in IR22 (compared to optimal) and fitness reductions on IR62 compared to IR22 at each exposure temperature. The optimal temperature for adult longevity was 20 °C (Fig. 6a). Resistance had its greatest effects on longevity at 20 and 25 °C (significantly higher than at 15, 30 or 35 °C). The combined effects of both temperature and resistance gene(s) resulted in highest mortalities at 25 °C (Table 4; Fig. 6a). Egg-laying was highest at 30 °C with significantly lower numbers of eggs laid at 15, 20 and 35 °C. However, resistance was most effective at 25 °C. This mismatch between optimal temperatures for egg laying and host resistance to oviposition produced a significant decline in the combined reducing effects of temperature and resistance at 30 °C (Table 4; Fig. 6b).

**Table 4:** F-values from univariate GLMs for factors producing a decline in fitness of *Nilaparvata lugens* on resistant rice over a range of temperatures (see also Fig. 5).

Parameter	F-values^1^		
	Temperature-related declines	Resistance-related declines	Combined reduction in fitness
Adult longevity^2^	5.647**	18.292***	3.626*
Eggs laid^2^	7.468***	3.341*	5.000**
Nymph survival^3^	16.534***	2.793 ns	18.788***
Nymph biomass^3^	60.542***	63.660***	7.041***

^1^ ns = *P* > 0.05, *P ≤ 0.05, **P ≤ 0.01, ***P ≤ 0.001.

^2^Degrees of freedom = 4,12; no significant effect of run.

^3^Degrees of freedom = 4,16; no significant effect of run.

Nymph mortality was highest at 35 °C and significantly higher than at 20–30 °C. Temperature had no effect on the functioning of resistance against nymph survival and the overall effects of the resistance gene(s) on survival were low such that combined temperature and resistance effects were greatest at 15 and 35 °C (Table 4; Fig. 6c). 25 °C was the optimal temperature at which nymphs gained biomass (Fig. 6d). Biomass declined at higher (30–35 °C) and lower (15–20 °C) temperatures. Resistance was most effective at 25 °C and moderately effective at 20 and 30 °C. Nevertheless, the lowest reductions in weight gain were observed at 25 °C because of the large effect of optimal temperature on nymph development and weight gain (Table 4; Fig. 6d).

## Discussion

Recently, Horgan et al.^37^ reviewed knowledge of the thermal niches of the brown planthopper. These authors indicated that whereas a number of studies had defined temperature tolerances and development thresholds for different planthopper populations, few studies had focused on the indirect, plant-mediated effects of temperature. Low temperature limits for nymphs and adults are between 8–16 °C with chill-comas at ≈ 3 °C (adult) and ≈ 7 °C (nymphs). At the other extreme, high temperatures for development are between 34–37 °C (nymphs) and 36–40 °C (adults) with heat-coma temperatures at 37–41 °C (nymphs) and 40–43 °C (adults)^37^. In the present study, all planthoppers died within two days of exposure to 40 °C (see methods), confirming that lethal temperatures for adults and nymphs occur at about that temperature. At 35 °C, nymph survival declined to < 20% on both rice varieties, nymph weights also declined, and development was delayed with no adults emerging. One of the most significant effects of high temperatures (e.g., ≈ 35 °C) is to alter the binding of enzymes (i.e., shifting the Michaelis constant^42^) affecting interactions between enzymes and their substrates. This will affect the functioning of important metabolites including digestive proteases and juvenile hormones^43,44^, thereby retarding development—as observed in our experiments with planthopper nymphs. Many of the effects of high temperatures can also be attributed to the loss of YLS: we monitored YLS in planthoppers exposed during 3 days to 35 °C and found that densities declined by 56–84%, with greater losses in the susceptible variety. Because of the essential role of YLS in planthopper nutrition, aposymbiotic nymphs fail to gain weight or develop to adults^21,24–26^. Furthermore, YLS are abundant in gravid females and are passed to the eggs prior to oviposition^24^. The removal of YLS from females does not affect their normal survival and development; however, it does affect reproduction and egg development^25^. We did not examine hatchability in the present study, but because YLS densities were significantly reduced at 35 °C, we suggest that any eggs produced at that temperature were unlikely to develop. The effects of temperature on hatchability of brown planthopper eggs have been reviewed by Horgan et al.^37^ – hatchability declines to < 40% at temperatures of ≥ 35 °C.

Low temperatures directly affect insect herbivores by increasing lags in neural signal transmission and by reducing the insect’s ability to generate action potentials in muscle^42,45^. Juvenile hormones can also fail at certain low temperatures^43^. However, the threshold temperatures for such effects are species-specific^45^. Based on the failure of nymphs to gain weight in our experiments, we suggest that neural and muscle functions were already reduced at 15 °C; this caused a decline in food intake, and the nymphs appeared sluggish and were less responsive to mechanical stimuli. At both 15 and 20 °C, nymphs had delayed development and failed to emerge as adults. Such effects can also be due to changes in rice plant physiology at low temperatures including a reduction in concentrations of soluble sugars (i.e., < 20°C^34^). For example, similar survival, growth rates and development rates of nymphs on IR22 and IR62 at 20 °C in our experiments, indicate that although nymphs consumed sufficient phloem to gain body weight, the phloem sap was of relatively poor quality at that temperature, even on IR22. Unlike nymphs, adult planthoppers survived for longer at low temperatures; however, despite this longevity, the adults displayed only weak oviposition cycles (20 °C) or displayed only an initial, first cycle of egg laying (15 °C). These patterns depict a lower success in ovariole development at temperatures of ≤ 20 °C, even on IR22. Nymphs on IR22 gained the greatest biomass at 25 °C. The nymphs also had high survival and rapid development at 25 °C, albeit with marginally faster development at 30 °C. Meanwhile, adults deposited larger egg batches (on both varieties) at 30 °C. Changes in planthopper fitness at these temperatures (25–30 °C) are important for understanding the consequences of global warming for herbivore pests. As temperatures increase between the lowest and highest tolerable temperatures for normal planthopper development, the insects are predicted to increase feeding activity and may gain increased nutrients from their rice host^46^. For example, concentrations of soluble sugars increase in rice and concentrations of several defensive allelochemicals decline as temperatures rise (including in susceptible varieties^33,47^). Furthermore, the activity of planthopper feeding effectors such as endo-β-1,4-glucanase increases as temperatures rise (i.e., from 27 to 37 °C)^48^. We suggest that changes such as these increase egg-laying and nymph development rates at 30 °C.

Our results indicate that IR62 maintains strong resistance against the Laguna (Philippines) population of the brown planthopper. In our study, IR62 exhibited clear antibiosis effects on planthopper nymphs. Rice responds to planthopper feeding by activating both the Jasmonic Acid (JA) and Salicylic Acid (SA) pathways. This induces a range of responses in both susceptible and resistant varieties, including augmentation in the concentrations of a wide variety of defensive secondary chemicals; however, compared to susceptible varieties the rate and magnitude of such responses is higher in varieties with the *BPH32* gene^29^. Rice lines with the *BPH32* gene also produce a range of constitutively expressed defense chemicals. For example, Stevenson et al.^28^ have shown that high schaftoside concentrations in varieties derived from Rathu Heenati were directly associated with planthopper mortality^28^. Furthermore, in a study by Saxena and Okech^30^, volatiles from Rathu Heenati and PTB33 (both of which share resistance genes with IR62) were shown to increase adult and nymph mortality and reduce adult feeding. The volatiles also reduced female settling^30^. Low and high temperatures can affect the production of constitutively expressed defensive secondary metabolites in rice^33,34^ and may alter the efficiency of the JA and SA pathways^49^. However, the apparent loss of host resistance at 35 °C in our study was mainly due to severe direct effects of very high temperatures on the planthopper and its YLS that obscured any effects of resistance on nymphs, and much of the effects on adults. However, resistance still functioned to reduce fitness (by ≈ 20%) at the higher temperature. Similarly, low feeding rates at 15 °C, likely reduced nymph exposure to rice defenses and obscured any effects of the resistance gene(s) at low temperatures.

IR62 reduces egg-laying of planthoppers in two ways: firstly, antixenosis deters females from settling and ovipositing on the plant; secondly, antibiosis reduces nutrient intake by gravid females and the conversion of nutrients to eggs. In choice studies, avirulent planthoppers tend to lay fewer eggs on IR62 than on susceptible varieties (e.g., TN1 and IR22) – although the effect can be weak^21^. In our experiments, adults were confined to their host plants. Reduced egg-laying in such non-choice experiments is mainly due to a lower production of eggs while feeding on the adult host plant. Our results clearly indicate that adults (≈ 20%) on IR22 exhibited three oviposition cycles (at 25 and 30 °C, and to some extent at 20 °C). Similar cycles have been observed in the green rice leafhopper, *Nephotettix cincticeps*^50^, but to our knowledge, they have not been observed previously in the brown planthopper. Based on the feeding history of the insects in our experiments, these oviposition cycles were due to resources attained during nymph development on the natal host (in our case on TN1) with further ovariole development on the adult host (1st cycle), as well as acquisition of resources from the adult host during the 2nd and 3rd cycles. Planthoppers on IR22 produce large numbers of eggs relative to planthoppers on TN1 and other susceptible hosts^23^, supporting the idea that the large numbers of eggs produced during the 2nd and 3rd cycles were due to resources from the adult host, IR22. On IR62, adults failed to produce a 2nd or 3rd cycle – even where they survived for up to 10 (25 °C) or 15 (30 °C) days. This indicates that the eggs deposited on IR62 were mainly derived from ovarioles produced during the pre-oviposition stage when planthoppers were still on the natal host TN1. Defenses in IR62 reduced the number of eggs per batch at 20, 25 and 35 °C, but had no apparent effect at 15 °C. Reductions in the size of batches on IR62 (compared to the 1st cycle on IR22) suggest that the planthoppers failed to acquire extra nutrients to support ovariole maturation—supporting the idea that resistance is related to antifeedants in the host phloem. This effect was greatest at 25 °C (see also Lu et al.^51^). However, at 30 °C, egg numbers were relatively high on both IR22 and IR62 indicating that the planthoppers successfully acquired further resources from the plants to increase egg production at that temperature. Because IR62 was effective against nymphs at 30 °C, but less effective against adults at the same temperature, we suggest that the defenses of IR62 were compromised by the increased activity of adults at the higher temperature together with a greater feeding capacity (as suggested by trends in egg production). In a similar case, Havko et al.^35^ found that at relatively high temperatures (i.e., 29 °C versus 22 °C), high feeding rates of the cabbage looper (*Trichoplusia ni*) on Arabidopsis overwhelmed JA-mediated defenses; but the high temperature did not affect the expression of JA-responsive genes or the production of glucosinolates. One important mechanism by which phloem feeders neutralize host defenses is by consuming large amounts of xylem to dilute phloem-based toxins. High production of xylem-based honeydew has been reported for planthoppers feeding on IR62, even after several generations of adaptation^23^. Detailed studies on the production and actions of defense metabolites across gradients of tolerable temperatures are recommended to further elucidate the mechanisms leading to a lower efficiency of antibiosis defenses against adult planthoppers in rice at elevated temperatures. Currently, a range of near-isogenic rice lines with planthopper resistant genes are available to support further research in this area^17,32^.

IR62 has been widely planted in Cambodia and in Mindanao (Philippines); although current rates of adoption are not known^19^. Temperatures in these regions have increased in recent decades^52,53^. For example, average temperature anomalies in the Philippines have increased by about 0.1 °C each decade since the 1950s^53^. Over the same period, the annual temperature anomaly increased by about 0.23 °C each decade in Cambodia^52^. During 2019, temperatures of 30 + °C were recorded in Malaybalay (Mindanao) on 148 days and in Kampong-Chhnang (Cambodia) on 349 days, with minimum temperatures above 27–28 °C during extended periods at the latter site between February and May^54^. At such temperatures, *BPH32*-derived resistance against immigrating females could be comprised for much of the time. Potential climate-related reductions in the efficiency of host plant resistance, as depicted in the present study, indicate that rice producers must broaden their pest management actions to increase the resilience of future crops. This is further highlighted by observations that several agrochemicals will increase the tolerance of brown planthoppers to adverse high temperatures^55,56^. Rice production systems that incorporate host plant resistance as a component of landscape approaches to promote the diversity of natural enemies^13,15,16^ will enhance the resilience (including the durability) of novel resistance genes and prevent losses from insect pests as global temperatures continue to increase.

## Materials and methods

### Brown planthopper

We used planthoppers from a colony maintained at the International Rice Research Institute (IRRI). The colony was initiated in 2009 with > 500 wild-caught individuals from Laguna Province (Philippines: 14°10′N, 121°13′E). Planthoppers from the region have noted virulence against a range of resistance genes including *BPH1*, *BPH2*, *BPH5*, *BPH7*, *BPH8*, *BPH18*, *BPH25* and *BPH26*^18^. The planthoppers were reared continuously on a susceptible variety, TN1 (≥ 30-day old rice plants), in wire mesh cages (91.5 × 56.5 × 56.5 cm; H × L × W). The colony was kept under greenhouse conditions (26–37 °C, 12:12 day:night [D:N]). Feeding plants were replaced every 3–5 days.

### Host plants

IR62 is a modern rice variety with confirmed resistance to brown planthopper populations from South and Southeast Asia^18^. IR62 acquired resistance from the Indian donor variety PTB33^19^. PTB33 possesses at least two genes for resistance to the planthopper (*BPH32* and *BPH26* [synonym with *BPH2*]^20^). IR62 is a promising source of resistance for future rice breeding because it appears counter-selective for virulence against *BPH1* and *BPH2*^22^. We used the variety IR22 as a susceptible control in our experiments. IR22 has no known resistance to planthoppers. The variety has similar phenological and morphological development to IR62^19^. Seeds of the two varieties were acquired through the IRRI Germplasm Collection. The seeds were germinated in a greenhouse and planted at 5–6 days after sowing (DAS) to #0 pots (7 × 11 cm: H × D) filled with paddy soil. Rice plants received no applications of pesticides or fertilizers. The rice plants were placed inside the corresponding climate chambers until they were used in bioassays (see below).

### Temperature bioassays

Bioassays were conducted in chambers with the Conviron CMP6050 Control System (Conviron, Winnipeg, Canada). Temperature treatments were rotated between four separate chambers – with the temperature settings changed after each run of the experiments. For experiments with nymphs, a fifth replicate for each temperature was conducted by randomly assigning the temperatures among chambers. There were between three and five subsamples (i.e., rearing cages – see below) per variety and time replicate, with subsamples randomized within the chambers. Temperatures ranged from 15 to 35 °C, with relative humidity maintained at 80% across all temperatures. The Conviron system maintained humidity at high and low temperatures using integrated fine-drop misters. We used a 12 h: 12 h [day: night] light regime at all temperatures. The temperature range represented a low temperature at which nymphs can survive and develop, and the critical maximum temperature (CT_max_) for brown planthopper nymphs, respectively^37^. We also conducted experiments at 40 °C; however, nymphs and adults failed to survive beyond 2 days and the adults did not lay eggs. Further information on the responses by brown planthopper adults and nymphs to temperatures ranging from 15 to 40 °C have been presented by Horgan et al.^37^. The bioassays were conducted as follows:

Oviposition experiments: Plants of each variety were individually covered with acetate rearing cages (50 × 10 cm: H × D). The cages had a mesh top to allow air circulation. A single mated gravid female was introduced to each cage at 20 DAS using a suction aspirator. Temperatures were 15, 20, 25, 30 and 35 °C. Temperatures were replicated across the chambers (i.e., N = 4), with chambers assigned randomly to each temperature. Each replicate consisted of continual observations from one day to 20 days after caging the females. The plants under each acetate cage were changed daily and the condition of the adults noted (i.e., surviving or dead). Plants that were exposed to females were dissected to count the numbers of egg clusters and the numbers of eggs per cluster. Replicates for each complete set of temperatures (henceforth a ‘run’) took ≈ 60 days to complete.

Nymph survival and development: Sufficient rice seedlings were prepared to be able to assess daily nymph survival and development at each temperature through destructive sampling (i.e., 15 days × 5 subsamples × 2 varieties = 150 seedlings per temperature replicate [up to 300 seedlings for three of the replicates at 15 °C]). Each temperature was replicated five times (N = 5) as described above. Ten newly emerged nymphs were placed on plants (one plant per cage) of each variety at 20 DAS under each temperature treatment. The plants were covered with acetate rearing cages (50 × 10 cm: H × D) with mesh widows for ventilation. Nymphs were allowed to feed and develop for 15 days with groups of ten plants (susceptible and resistant) randomly selected for sampling per temperature, per day. The number of survivors and their developmental stages were recorded and the insects were dried in an oven for 5 days and weighed to estimate total nymph biomass per plant. Each run usually took ≈ 60 days to complete.

### Yeast like symbiont densities after optimal and high temperatures

Ten plants of IR22 and 10 of IR62 (20 DAS) were each infested with 10 neonates. The plants were covered with acetate rearing cages (50 × 10 cm: H × D) with mesh widows for ventilation. The plants were divided into two groups (5 × IR22 and 5 × IR62 each) and placed in chambers at 25 °C and 35 °C (relative humidity 80% and 12: 12 h light: darkness) for 3 days after which the plants were placed in an insectary at 27 °C until neonates were 10 days old. Nymphs were allowed to develop for a total of 10 days to improve estimates of symbiont densities. The nymphs were then weighed. Yeast-like symbiont densities were estimated using the method described by Ferreter et al.^23^. The nymphs were homogenized in 500 μl physiological saline solution (0.9% NaCL). For each sample, an aliquot of 10 μl was transferred to a hemocytometer and the YLS counted under a compound microscope (× 40 magnification)^23^.

### Data analyses

Results from the oviposition and nymph survival experiments were analyzed using repeated measures general linear models (GLM) with days after first exposure as the repeated measure and temperature, variety and their interaction as main factors. Because experimental runs took up to 60 days to complete, we included ‘run’ as a blocking factor in our analyses to control for possible changes in the planthopper colony during the time that the research was conducted (e.g., short-term temperature assimilation, etc.). Survival, the numbers of egg batches, and the numbers of eggs laid were analyzed only for the first 6 days of the experiment because of low survival after that time at some temperatures. Similarly, because egg batches were produced by planthoppers across all replicates and treatments for only 4 days, batch numbers per female, eggs per female, and the size of egg batches were each analyzed only for the first 4 days – representing the peak of the first oviposition cycle. We analyzed nymph survival and nymph biomass during 15 days using repeated measures GLMs. Prior to analyses, survival was arcsine-transformed; nymph biomass, the number of egg batches, the numbers of eggs, and batch size per female were log-transformed; and the total number of batches and eggs were ranked.

Nymph biomass and survival at the end of 15 days, and the total number of batches and eggs laid, adult longevity, batches and eggs per females, and batch size at the end of each experiment (including all days) were further analyzed using univariate GLM. For these analyses, longevity was measured as the time to 50% and 100% mortality. Batch number, total egg number, the number of eggs per planthopper, and batch size were log-transformed before analyses. We analyzed nymph development based on the time for 50% of nymphs to develop to second (N1), third (N2) and fourth instars (N3). We used a multiple GLM to analyze nymph development times (N1, N2, N3) and univariate GLM to analyze the proportion of nymphs developing to adults at 25 and 30 °C with ‘run’ included as a blocking factor (see above)^37^.

We estimated the separate effects of temperature and host plant resistance on planthopper fitness. To estimate the effects of temperature we calculated percentage reductions in fitness measured on IR22 at each temperature (T) compared to optimal temperatures for each parameter (i.e., the reduction in fitness due to sub-optimal temperature at T_a_ = 1-(fitnessT_a_/fitnessT_optimal_), where a = 15 °C, 20 °C, etc.). To estimate the effects of host resistance, we calculated percent reductions in fitness at each temperature by comparing fitness measures on IR62 and IR22 (i.e., the reduction in fitness due to resistance at T_a_ = 1-(fitness_IR62_/fitness_IR22_)_Ta_, where a = 15 °C, 20 °C, etc.). Total reductions in fitness were then determined as the sum of both fitness reductions at each temperature. Reductions calculated as such for adult longevity (until 50% mortality), total number of eggs laid, nymph survival at 15 days, and nymph weight at 15 days, were analyzed using univariate GLMs.

Post-hoc Tukey tests were performed for the factor ‘temperature’. Residuals were plotted following parametric analyses to test for normality and homogeneity.

## Supplementary Information

Supplementary Information 1.

Supplementary Information 2.

## References

[1] Lüthi D (2008). High-resolution carbon dioxide concentration record 650,000–800,000 years before present. Nature.

[2] NASA. *Global Vital Signs: Vital Signs of the Planet* https://climate.nasa.gov/ (2019).

[3] Pachauri, R. K. et al. Climate Change 2014: synthesis report. Fifth Assessment Report on the Intergovernmental Panel on Climate Change 151. Geneva, Switzerland. (2014)

[4] Bale JS (2002). Herbivory in global climate change research: direct effects of rising temperature on insect herbivores. Glob. Change Biol..

[5] Forrest JRK (2016). Complex responses of insect phenology to climate change. Curr. Opin. Insect Sci..

[6] Food and Agriculture Organisation of the United Nations. *FAOSTAT Crops* http://www.fao.org/faostat/en/#home (2019).

[7] Lobell DB, Schlenker W, Costa-Roberts J (2011). Climate trends and global crop production since 1980. Science.

[8] Ray DK (2019). Climate change has likely already affected global food production. PLoS ONE.

[9] Ali MP (2014). Will climate change affect outbreak patterns of planthoppers in Bangladesh?. PLoS ONE.

[10] Ali MP (2019). Increased temperature induces leaffolder outbreak in rice field. J. Appl. Entomol..

[11] Hu, G. *et al.* Outbreaks of the brown planthopper *Nilaparvata lugens* (Stål) in the Yangtze River Delta: immigration or local reproduction? *PLoS ONE* **9**, e88973 (2014). 10.1371/journal.pone.0088973 PMC3928339 24558459

[12] Yukawa J (2009). Northward range expansion by *Nezara viridula* (Hemiptera: Pentatomidae) in Shikoku and Chugoku Districts, Japan, possibly due to global warming. Appl. Entomol. Zool..

[13] Horgan FG (2018). Integrating gene deployment and crop management for improved rice resistance to Asian planthoppers. Crop Prot..

[14] Ali MP (2019). Establishing next-generation pest control services in rice fields: eco-agriculture. Sci. Rep..

[15] Horgan FG (2019). Effects of vegetation strips, fertilizer levels and varietal resistance on the integrated management of arthropod biodiversity in a tropical rice ecosystem. Insects.

[16] Horgan FG, Jabran K, Florantine S, Chauhan BS (2020). Potential for an impact of global climate change on insect herbivory in cereal crops. Crop Protection Under Climate Change.

[17] Fujita D, Kohli A, Horgan FG (2013). Rice resistance to planthoppers and leafhoppers. Crit. Rev. Plant Sci..

[18] Horgan FG (2015). Virulence of brown planthopper (*Nilaparvata lugens*) populations from South and South East Asia against resistant rice varieties. Crop Prot..

[19] Khush GS, Virk PS (2005). IR Varieties and Their Impact.

[20] Ren J (2016). *Bph32*, a novel gene encoding an unknown SCR domain-containing protein, confers resistance against the brown planthopper in rice. Sci. Rep..

[21] Horgan FG, Ferrater JB (2017). Benefits and potential trade-offs associated with yeast-like symbionts during virulence adaptation in a phloem-feeding planthopper. Entomol. Exp. Appl..

[22] Horgan FG, Garcia CPF, Haverkort F, de Jong PW, Ferrater JB (2020). Changes in insecticide resistance and host range performance of planthoppers artificially selected to feed on resistant rice. Crop Prot..

[23] Ferreter JB (2015). Varied responses by yeast-like symbionts during virulence adaptation in a monophagous phloem-feeding insect. Arthropod-Plant Interact..

[24] Ferrater JB, de Jong PW, Dicke M, Chen YH, Horgan FG (2013). Symbiont-mediated adaptation by planthoppers and leafhoppers to resistant rice varieties. Arthropod-Plant Interact..

[25] Lee YH, Hou RF (1987). Physiological roles of a yeast-like symbiote in reproduction and embryonic development of the brown planthopper, *Nilaparvata lugens*Stål. J. Insect Physiol..

[26] Hongoh Y, Ishikawa H (1997). Uric acid as a nitrogen resource for the brown planthopper, *Nilaparvata lugens*: studies with synthetic diets and aposymbiotic insects. Zool. Sci..

[27] Pan Y (2019). Identification of brown planthopper resistance gene *Bph32* in the progeny of a rice dominant genic male sterile recurrent population using genome-wide association study and RNA-seq analysis. Mol. Breed..

[28] Stevenson PC, Kimmins FM, Grayer RJ, Raveendranath S (1996). Schaftosides from rice phloem as feeding inhibitors and resistance factors to brown planthopper, *Nilaparvata lugens*. Entomol. Exp. Appl..

[29] Uawisetwathana U (2019). Global metabolite profiles of rice brown planthopper-resistant traits reveal potential secondary metabolites for both constitutive and inducible defenses. Metabolomics.

[30] Saxena RC, Okech SH (1985). Role of plant volatiles in resistance of selected rice varieties to brown planthopper, *Nilaparvata lugens* (Stål)(Homoptera; Delphacidae). J. Chem. Ecol..

[31] Kamolsukyeunyong W (2019). Identification of spontaneous mutation for broad-spectrum brown planthopper resistance in a large, long-term fast neutron mutagenized rice population. Rice.

[32] Nguyen CD (2019). The development and characterization of near-isogenic and pyramided lines carrying resistance genes to brown planthopper with the genetic background of japonica rice (*Oryza sativa* L.). Plants.

[33] Salim M, Saxena RC (1991). Temperature stress and varietal resistance in rice: effects on whitebackedplanthopper. Crop Sci..

[34] Wang B-J, Xu H-X, Zheng X-S, Fu Q, Lu Z-X (2010). High temperature modifies resistance performances of rice varieties to brown planthopper, *Nilaparvata lugens* (Stål). Rice Sci..

[35] Havko NE, Kapali G, Das MR, Howe GA (2020). Stimulation of insect herbivory by elevated temperature outweighs protection by the jasmonate pathway. Plants.

[36] Yuan JS, Himanen SJ, Holopainen JK, Chen F, Stewart CN (2009). Smelling global climate change: mitigation of function from plant volatile organic compounds. Trends Ecol. Evol..

[37] Horgan FG, Arida A, Ardestani G, Almazan MLP (2020). Temperature-dependent oviposition and nymph performance reveal distinct thermal niches of coexisting planthoppers with similar thresholds for development. PLoS ONE.

[38] Srinivas M, Devi RS, Varmaand NRG, Jagadeeshwar R (2020). Interactive effect of temperature and CO_2_ on resistance of rice genotypes to brown planthopper, *Nilaparvata lugens* (Stål.). J. Entomol. Zool. Stud..

[39] Zhang L, Wu J, Chen B (1990). Influence of temperature and light on expression of resistance in rice to the brown planthopper, *Nilaparvata lugens* (Homoptera: Delphacidae). J. South China Agric. Univ..

[40] Romena S, Saxena R (1988). Screening for resistance to whitebacked planthopper, *Sogatella furcifera* (Horvath): effect of temperature on seedling damage.

[41] Horgan FG (2018). Resistance and tolerance to the brown planthopper, *Nilaparvata lugens* (Stål), in rice infested at different growth stages across a gradient of nitrogen applications. Field Crops Res..

[42] Neven LG (2000). Physiological responses of insects to heat. Postharvest Biol. Technol..

[43] Bühler A, Lanzrein B, Wille H (1983). Influence of temperature and carbon dioxide concentration on juvenile hormone titre and dependent parameters of adult worker honey bees (*Apis mellifera* L). J. Insect Physiol..

[44] Foissac X, Edwards M, Du J, Gatehouse A, Gatehouse J (2002). Putative protein digestion in a sap-sucking homopteran plant pest (rice brown plant hopper; *Nilaparvata lugens*: Delphacidae)—identification of trypsin-like and cathepsin B-like proteases. Insect Biochem. Mol. Biol..

[45] MacMillan HA, Sinclair BJ (2011). Mechanisms underlying insect chill-coma. J. Insect Physiol..

[46] Vailla S, Muthusamy S, Konijeti C, Shanker C, Vattikuti JL (2019). Effects of elevated carbon dioxide and temperature on rice brown planthopper, *Nilaparvata lugens* (Stål) populations in India. Curr. Sci..

[47] Wang B, Xu H, Zheng X, Fu Q, Lu X (2010). Effect of temperature on resistance of rice to brown planthopper, *Nilaparvata lugens*. Chin. J. Rice Sci..

[48] Ji R (2017). A salivary endo-β-1, 4-glucanase acts as an effector that enables the brown planthopper to feed on rice. Plant Physiol..

[49] Venkatesh J, Kang B-C (2019). Current views on temperature-modulated R gene-mediated plant defense responses and tradeoffs between plant growth and immunity. Curr. Opin. Plant Biol..

[50] Murai M, Kiritani K (1970). Influence of parental age upon the offspring in the green rice leafhopper, *Nephotettix cincticeps* Uhler (Hemiptera: Deltocephalidae). Appl. Entomol. Zool..

[51] Lu K (2016). Nutritional signaling regulates vitellogenin synthesis and egg development through juvenile hormone in *Nilaparvata lugens* (Stål). Int. J. Mol. Sci..

[52] Thoeun HC (2015). Observed and projected changes in temperature and rainfall in Cambodia. Weather Clim. Extremes.

[53] PAGASA. *Observed Climate Trends and Projected Climate Change in the Philippines*. (Philippine Athmospheric, Geophysical and Astronomical Services Administration (PAGASA), Philippines, (2018).

[54] Yu Media Group. Weather Atlas: weather around the world - list of countries. http://www.weather-atlas.com/en/countries (2020).

[55] You LL (2016). Driving pest insect populations: agricultural chemicals lead to an adaptive syndrome in *Nilaparvata Lugens* Stål (Hemiptera: Delphacidae). Sci. Rep..

[56] Ge LQ (2013). Molecular basis for insecticide-enhanced thermotolerance in the brown planthopper *Nilaparvata lugens* Stål (Hemiptera: Delphacidae). Mol. Ecol..

