# Supplementary material for: Elevated temperatures diminish the effects of a highly resistant rice variety on the brown planthopper
Source: Sci Rep. 2021 Jan 8;11:262. doi: 10.1038/s41598-020-80704-4 (PMC7794346; doi:10.1038/s41598-020-80704-4)
Supplement: Supplementary file 1 — Supplementary Information 1. [file 41598_2020_80704_MOESM1_ESM.docx]

**Elevated temperatures diminish the effects of a highly resistant rice variety on the brown planthopper**

Finbarr G. Horgan, Arriza Arida, Goli Ardestani, Maria Liberty P. Almazan

Supplementary Information

Nymph survival, biomass and development data

| plant | temp (degC) | day of obs'n | Run | Instar 1 | Instar 2 | Instar 3 | Instar 4 | Instar 5 | Adult | nymph biomass (g) | Plant biomass (g) |
| --- | --- | --- | --- | --- | --- | --- | --- | --- | --- | --- | --- |
| IR22 | 15 | 1 | 1 | 0.50 | 4.00 | 5.00 | 0.00 | 0.00 | 0.00 | 0.06 |  |
| IR22 | 15 | 1 | 2 | 0.00 | 3.00 | 6.00 | 0.00 | 0.00 | 0.00 | 0.06 |  |
| IR22 | 15 | 1 | 3 | 2.50 | 4.00 | 0.50 | 0.00 | 0.00 | 0.00 | 0.04 |  |
| IR22 | 15 | 1 | 4 | 8.67 | 0.00 | 0.00 | 0.00 | 0.00 | 0.00 | 0.09 | 0.38 |
| IR22 | 15 | 1 | 5 | 8.67 | 0.00 | 0.00 | 0.00 | 0.00 | 0.00 | 0.07 | 0.30 |
| IR22 | 15 | 2 | 1 | 0.50 | 3.00 | 2.00 | 0.00 | 0.00 | 0.00 | 0.04 |  |
| IR22 | 15 | 2 | 2 | 0.00 | 0.50 | 5.50 | 0.00 | 0.00 | 0.00 | 0.06 |  |
| IR22 | 15 | 2 | 3 | 0.00 | 3.50 | 6.50 | 0.00 | 0.00 | 0.00 | 0.07 |  |
| IR22 | 15 | 2 | 4 | 8.00 | 0.00 | 0.00 | 0.00 | 0.00 | 0.00 | 0.08 | 0.41 |
| IR22 | 15 | 2 | 5 | 7.33 | 0.00 | 0.00 | 0.00 | 0.00 | 0.00 | 0.09 | 0.44 |
| IR22 | 15 | 3 | 1 | 1.00 | 1.50 | 5.50 | 1.00 | 0.00 | 0.00 | 0.08 |  |
| IR22 | 15 | 3 | 2 | 0.00 | 0.00 | 0.50 | 4.50 | 0.00 | 0.00 | 0.06 |  |
| IR22 | 15 | 3 | 3 | 0.00 | 1.50 | 6.50 | 0.00 | 0.00 | 0.00 | 0.07 |  |
| IR22 | 15 | 3 | 4 | 7.67 | 0.00 | 0.00 | 0.00 | 0.00 | 0.00 | 0.12 | 0.40 |
| IR22 | 15 | 3 | 5 | 9.67 | 0.00 | 0.00 | 0.00 | 0.00 | 0.00 | 0.15 | 0.40 |
| IR22 | 15 | 4 | 1 | 0.50 | 1.50 | 3.50 | 2.50 | 0.00 | 0.00 | 0.09 |  |
| IR22 | 15 | 4 | 2 | 0.00 | 0.00 | 1.50 | 5.50 | 0.00 | 0.00 | 0.08 |  |
| IR22 | 15 | 4 | 3 | 0.00 | 0.00 | 1.00 | 6.00 | 0.00 | 0.00 | 0.08 |  |
| IR22 | 15 | 4 | 4 | 8.67 | 0.00 | 0.00 | 0.00 | 0.00 | 0.00 | 0.14 | 0.54 |
| IR22 | 15 | 4 | 5 | 10.00 | 0.00 | 0.00 | 0.00 | 0.00 | 0.00 | 0.14 | 0.60 |
| IR22 | 15 | 5 | 1 | 0.00 | 0.00 | 3.00 | 4.50 | 0.00 | 0.00 | 0.11 |  |
| IR22 | 15 | 5 | 2 | 0.00 | 0.00 | 1.00 | 3.50 | 2.00 | 0.00 | 0.09 |  |
| IR22 | 15 | 5 | 3 | 0.00 | 0.00 | 2.50 | 4.00 | 2.00 | 0.00 | 0.12 |  |
| IR22 | 15 | 5 | 4 | 8.33 | 0.00 | 0.00 | 0.00 | 0.00 | 0.00 | 0.18 | 0.55 |
| IR22 | 15 | 5 | 5 | 9.33 | 0.00 | 0.00 | 0.00 | 0.00 | 0.00 | 0.18 | 0.57 |
| IR22 | 15 | 6 | 1 | 1.00 | 1.50 | 1.00 | 4.00 | 0.00 | 0.00 | 0.10 |  |
| IR22 | 15 | 6 | 2 | 0.00 | 0.00 | 0.00 | 1.00 | 7.00 | 0.00 | 0.14 |  |
| IR22 | 15 | 6 | 3 | 0.00 | 0.00 | 0.00 | 1.00 | 8.00 | 0.00 | 0.14 |  |
| IR22 | 15 | 6 | 4 | 8.00 | 0.00 | 0.00 | 0.00 | 0.00 | 0.00 | 0.17 | 0.68 |
| IR22 | 15 | 6 | 5 | 8.67 | 0.00 | 0.00 | 0.00 | 0.00 | 0.00 | 0.16 | 0.63 |
| IR22 | 15 | 7 | 1 | 0.00 | 0.00 | 0.50 | 3.00 | 4.50 | 0.00 | 0.15 |  |
| IR22 | 15 | 7 | 2 | 0.00 | 0.00 | 0.00 | 0.50 | 8.00 | 0.00 | 0.18 |  |
| IR22 | 15 | 7 | 3 | 0.00 | 0.00 | 0.00 | 0.00 | 8.00 | 0.00 | 0.15 |  |
| IR22 | 15 | 7 | 4 | 5.33 | 3.00 | 0.00 | 0.00 | 0.00 | 0.00 | 0.17 | 0.65 |
| IR22 | 15 | 7 | 5 | 2.33 | 5.67 | 0.00 | 0.00 | 0.00 | 0.00 | 0.18 | 0.53 |
| IR22 | 15 | 8 | 1 | 0.00 | 0.00 | 0.00 | 0.50 | 8.00 | 0.00 | 0.18 |  |
| IR22 | 15 | 8 | 2 | 0.00 | 0.00 | 0.00 | 1.00 | 6.00 | 0.00 | 0.12 |  |
| IR22 | 15 | 8 | 3 | 0.00 | 0.00 | 0.00 | 0.00 | 9.00 | 0.00 | 0.17 |  |
| IR22 | 15 | 8 | 4 | 1.00 | 7.00 | 0.00 | 0.00 | 0.00 | 0.00 | 0.17 | 0.58 |
| IR22 | 15 | 8 | 5 | 1.33 | 7.00 | 0.00 | 0.00 | 0.00 | 0.00 | 0.23 | 0.72 |
| IR22 | 15 | 9 | 1 | 0.00 | 0.00 | 0.00 | 0.00 | 8.50 | 0.00 | 0.18 |  |
| IR22 | 15 | 9 | 2 | 0.00 | 0.00 | 0.00 | 0.50 | 8.00 | 0.00 | 0.17 |  |
| IR22 | 15 | 9 | 3 | 0.00 | 0.00 | 0.00 | 0.00 | 8.00 | 0.00 | 0.18 |  |
| IR22 | 15 | 9 | 4 | 1.67 | 6.67 | 0.00 | 0.00 | 0.00 | 0.00 | 0.24 | 0.57 |
| IR22 | 15 | 9 | 5 | 3.33 | 3.00 | 0.00 | 0.00 | 0.00 | 0.00 | 0.21 | 0.72 |
| IR22 | 15 | 10 | 1 | 0.00 | 0.00 | 0.00 | 0.00 | 9.50 | 0.00 | 0.22 |  |
| IR22 | 15 | 10 | 2 | 0.00 | 0.00 | 0.00 | 0.50 | 7.00 | 0.00 | 0.17 |  |
| IR22 | 15 | 10 | 3 | 0.00 | 0.00 | 0.00 | 0.00 | 7.50 | 0.00 | 0.15 |  |
| IR22 | 15 | 10 | 4 | 0.00 | 7.33 | 0.00 | 0.00 | 0.00 | 0.00 | 0.25 | 0.71 |
| IR22 | 15 | 10 | 5 | 0.00 | 9.33 | 0.00 | 0.00 | 0.00 | 0.00 | 0.39 | 0.72 |
| IR22 | 15 | 11 | 1 | 0.00 | 0.00 | 0.00 | 0.00 | 8.50 | 0.00 | 0.18 |  |
| IR22 | 15 | 11 | 2 | 0.00 | 0.00 | 0.00 | 0.00 | 7.50 | 0.00 | 0.16 |  |
| IR22 | 15 | 11 | 3 | 0.00 | 0.00 | 0.00 | 0.00 | 8.50 | 0.00 | 0.17 |  |
| IR22 | 15 | 11 | 4 | 1.00 | 8.00 | 0.00 | 0.00 | 0.00 | 0.00 | 0.39 | 0.71 |
| IR22 | 15 | 11 | 5 | 0.33 | 5.67 | 0.00 | 0.00 | 0.00 | 0.00 | 0.23 | 0.64 |
| IR22 | 15 | 12 | 1 | 0.00 | 0.00 | 0.00 | 0.50 | 7.00 | 0.00 | 0.16 |  |
| IR22 | 15 | 12 | 2 | 0.00 | 0.00 | 0.00 | 0.00 | 9.00 | 0.00 | 0.19 |  |
| IR22 | 15 | 12 | 3 | 0.00 | 0.00 | 0.00 | 0.00 | 3.50 | 0.00 | 0.07 |  |
| IR22 | 15 | 12 | 4 | 0.00 | 6.67 | 1.33 | 0.00 | 0.00 | 0.00 | 0.36 | 0.58 |
| IR22 | 15 | 12 | 5 | 0.00 | 9.67 | 0.00 | 0.00 | 0.00 | 0.00 | 0.49 | 0.70 |
| IR22 | 15 | 13 | 1 | 0.00 | 0.00 | 0.00 | 1.00 | 7.50 | 0.00 | 0.23 |  |
| IR22 | 15 | 13 | 2 | 0.00 | 0.00 | 0.00 | 0.50 | 8.00 | 0.00 | 0.17 |  |
| IR22 | 15 | 13 | 3 | 0.00 | 0.00 | 0.00 | 0.00 | 9.50 | 0.00 | 0.20 |  |
| IR22 | 15 | 13 | 4 | 0.00 | 7.67 | 1.00 | 0.00 | 0.00 | 0.00 | 0.44 | 0.61 |
| IR22 | 15 | 13 | 5 | 0.00 | 5.33 | 3.67 | 0.00 | 0.00 | 0.00 | 0.46 | 0.71 |
| IR22 | 15 | 14 | 1 | 0.00 | 0.00 | 0.00 | 0.00 | 9.00 | 0.00 | 0.25 |  |
| IR22 | 15 | 14 | 2 | 0.00 | 0.00 | 0.00 | 0.00 | 6.00 | 0.00 | 0.13 |  |
| IR22 | 15 | 14 | 3 | 0.00 | 0.00 | 0.00 | 0.00 | 6.50 | 0.00 | 0.18 |  |
| IR22 | 15 | 14 | 4 | 0.00 | 3.33 | 6.00 | 0.00 | 0.00 | 0.00 | 0.45 | 0.76 |
| IR22 | 15 | 14 | 5 | 0.00 | 1.33 | 8.33 | 0.00 | 0.00 | 0.00 | 0.63 | 0.73 |
| IR22 | 15 | 15 | 1 | 0.00 | 0.00 | 0.00 | 0.00 | 6.50 | 0.00 | 0.28 |  |
| IR22 | 15 | 15 | 2 | 0.00 | 0.00 | 0.00 | 0.00 | 8.50 | 0.00 | 0.18 |  |
| IR22 | 15 | 15 | 3 | 0.00 | 0.00 | 0.00 | 0.00 | 8.00 | 0.00 | 0.25 |  |
| IR22 | 15 | 15 | 4 | 0.00 | 0.67 | 8.00 | 0.00 | 0.00 | 0.00 | 0.58 | 0.81 |
| IR22 | 15 | 15 | 5 | 0.00 | 1.00 | 6.67 | 0.00 | 0.00 | 0.00 | 0.59 | 0.90 |
| IR22 | 15 | 16 | 1 | 0.00 | 0.00 | 0.00 | 0.00 | 9.00 | 0.00 | 0.49 |  |
| IR22 | 15 | 16 | 2 | 0.00 | 0.00 | 0.00 | 0.00 | 9.50 | 0.00 | 0.22 |  |
| IR22 | 15 | 16 | 3 | 0.00 | 0.00 | 0.00 | 0.00 | 6.00 | 0.00 | 0.23 |  |
| IR22 | 15 | 16 | 4 | 0.00 | 0.00 | 0.00 | 0.00 | 8.17 | 0.00 | 0.31 |  |
| IR22 | 15 | 16 | 5 | 0.00 | 0.00 | 0.00 | 0.00 | 7.89 | 0.00 | 0.25 |  |
| IR22 | 15 | 17 | 1 | 0.00 | 0.00 | 0.00 | 0.00 | 9.50 | 0.00 | 0.43 |  |
| IR22 | 15 | 17 | 2 | 0.00 | 0.00 | 0.00 | 0.00 | 6.50 | 0.00 | 0.18 |  |
| IR22 | 15 | 17 | 3 | 0.00 | 0.00 | 0.00 | 0.00 | 5.50 | 0.00 | 0.13 |  |
| IR22 | 15 | 17 | 4 | 0.00 | 0.00 | 0.00 | 0.00 | 7.17 | 0.00 | 0.25 |  |
| IR22 | 15 | 17 | 5 | 0.00 | 0.00 | 0.00 | 0.00 | 6.39 | 0.00 | 0.19 |  |
| IR22 | 15 | 18 | 1 | 0.00 | 0.00 | 0.00 | 0.00 | 7.50 | 0.00 | 0.52 |  |
| IR22 | 15 | 18 | 2 | 0.00 | 0.00 | 0.00 | 0.00 | 7.00 | 0.00 | 0.18 |  |
| IR22 | 15 | 18 | 3 | 0.00 | 0.00 | 0.00 | 0.50 | 7.00 | 0.00 | 0.30 |  |
| IR22 | 15 | 18 | 4 | 0.00 | 0.00 | 0.00 | 0.17 | 7.17 | 0.00 | 0.33 |  |
| IR22 | 15 | 18 | 5 | 0.00 | 0.00 | 0.00 | 0.22 | 7.06 | 0.00 | 0.27 |  |
| IR22 | 15 | 19 | 1 | 0.00 | 0.00 | 0.00 | 0.00 | 7.50 | 0.00 | 0.41 |  |
| IR22 | 15 | 19 | 2 | 0.00 | 0.00 | 0.00 | 0.00 | 7.50 | 0.00 | 0.22 |  |
| IR22 | 15 | 19 | 3 | 0.00 | 0.00 | 0.00 | 0.50 | 8.00 | 0.00 | 0.30 |  |
| IR22 | 15 | 19 | 4 | 0.00 | 0.00 | 0.00 | 0.17 | 7.67 | 0.00 | 0.31 |  |
| IR22 | 15 | 19 | 5 | 0.00 | 0.00 | 0.00 | 0.22 | 7.72 | 0.00 | 0.28 |  |
| IR22 | 15 | 20 | 1 | 0.00 | 0.00 | 0.00 | 0.00 | 9.00 | 0.00 | 0.76 |  |
| IR22 | 15 | 20 | 2 | 0.00 | 0.00 | 0.00 | 0.00 | 4.00 | 0.00 | 0.13 |  |
| IR22 | 15 | 20 | 3 | 0.00 | 0.00 | 0.00 | 0.00 | 5.00 | 0.00 | 0.17 |  |
| IR22 | 15 | 20 | 4 | 0.00 | 0.00 | 0.00 | 0.00 | 6.00 | 0.00 | 0.36 |  |
| IR22 | 15 | 20 | 5 | 0.00 | 0.00 | 0.00 | 0.00 | 5.00 | 0.00 | 0.22 |  |
| IR22 | 15 | 21 | 1 | 0.00 | 0.00 | 0.00 | 0.00 | 6.00 | 0.00 | 0.54 |  |
| IR22 | 15 | 21 | 2 | 0.00 | 0.00 | 0.00 | 0.00 | 7.50 | 0.00 | 0.24 |  |
| IR22 | 15 | 21 | 3 | 0.00 | 0.00 | 0.00 | 0.00 | 7.50 | 0.00 | 0.31 |  |
| IR22 | 15 | 21 | 4 | 0.00 | 0.00 | 0.00 | 0.00 | 7.00 | 0.00 | 0.36 |  |
| IR22 | 15 | 21 | 5 | 0.00 | 0.00 | 0.00 | 0.00 | 7.33 | 0.00 | 0.30 |  |
| IR22 | 15 | 22 | 1 | 0.00 | 0.00 | 0.00 | 0.00 | 8.50 | 0.00 | 0.57 |  |
| IR22 | 15 | 22 | 2 | 0.00 | 0.00 | 0.00 | 0.00 | 6.50 | 0.00 | 0.19 |  |
| IR22 | 15 | 22 | 3 | 0.00 | 0.00 | 0.00 | 0.00 | 4.00 | 0.00 | 0.16 |  |
| IR22 | 15 | 22 | 4 | 0.00 | 0.00 | 0.00 | 0.00 | 6.33 | 0.00 | 0.31 |  |
| IR22 | 15 | 22 | 5 | 0.00 | 0.00 | 0.00 | 0.00 | 5.61 | 0.00 | 0.22 |  |
| IR22 | 15 | 23 | 1 | 0.00 | 0.00 | 0.00 | 0.00 | 9.50 | 0.00 | 0.63 |  |
| IR22 | 15 | 23 | 2 | 0.00 | 0.00 | 0.00 | 0.00 | 7.50 | 0.00 | 0.29 |  |
| IR22 | 15 | 23 | 3 | 0.00 | 0.00 | 0.00 | 0.00 | 6.50 | 0.00 | 0.27 |  |
| IR22 | 15 | 23 | 4 | 0.00 | 0.00 | 0.00 | 0.00 | 7.83 | 0.00 | 0.40 |  |
| IR22 | 15 | 23 | 5 | 0.00 | 0.00 | 0.00 | 0.00 | 7.28 | 0.00 | 0.32 |  |
| IR22 | 15 | 24 | 1 | 0.00 | 0.00 | 0.00 | 0.00 | 7.50 | 0.00 | 0.62 |  |
| IR22 | 15 | 24 | 2 | 0.00 | 0.00 | 0.00 | 0.00 | 8.50 | 0.00 | 0.34 |  |
| IR22 | 15 | 24 | 3 | 0.00 | 0.00 | 0.00 | 0.00 | 7.00 | 0.00 | 0.31 |  |
| IR22 | 15 | 24 | 4 | 0.00 | 0.00 | 0.00 | 0.00 | 7.67 | 0.00 | 0.42 |  |
| IR22 | 15 | 24 | 5 | 0.00 | 0.00 | 0.00 | 0.00 | 7.72 | 0.00 | 0.36 |  |
| IR22 | 15 | 25 | 1 | 0.00 | 0.00 | 0.00 | 0.00 | 9.50 | 0.00 | 0.42 |  |
| IR22 | 15 | 25 | 2 | 0.00 | 0.00 | 0.00 | 0.00 | 8.50 | 0.00 | 0.31 |  |
| IR22 | 15 | 25 | 3 | 0.00 | 0.00 | 0.00 | 0.00 | 7.00 | 0.00 | 0.33 |  |
| IR22 | 15 | 25 | 4 | 0.00 | 0.00 | 0.00 | 0.00 | 8.33 | 0.00 | 0.35 |  |
| IR22 | 15 | 25 | 5 | 0.00 | 0.00 | 0.00 | 0.00 | 7.94 | 0.00 | 0.33 |  |
| IR22 | 15 | 26 | 1 | 0.00 | 0.00 | 0.00 | 0.00 | 9.00 | 0.00 | 0.87 |  |
| IR22 | 15 | 26 | 2 | 0.00 | 0.00 | 0.00 | 0.00 | 7.50 | 0.00 | 0.30 |  |
| IR22 | 15 | 26 | 3 | 0.00 | 0.00 | 0.00 | 0.00 | 7.00 | 0.00 | 0.40 |  |
| IR22 | 15 | 26 | 4 | 0.00 | 0.00 | 0.00 | 0.00 | 7.83 | 0.00 | 0.53 |  |
| IR22 | 15 | 26 | 5 | 0.00 | 0.00 | 0.00 | 0.00 | 7.44 | 0.00 | 0.41 |  |
| IR22 | 15 | 27 | 1 | 0.00 | 0.00 | 0.00 | 0.00 | 1.50 | 0.00 | 0.23 |  |
| IR22 | 15 | 27 | 2 | 0.00 | 0.00 | 0.00 | 0.00 | 4.00 | 0.00 | 0.17 |  |
| IR22 | 15 | 27 | 3 | 0.00 | 0.00 | 0.00 | 0.00 | 7.50 | 0.00 | 0.43 |  |
| IR22 | 15 | 27 | 4 | 0.00 | 0.00 | 0.00 | 0.00 | 4.33 | 0.00 | 0.28 |  |
| IR22 | 15 | 27 | 5 | 0.00 | 0.00 | 0.00 | 0.00 | 5.28 | 0.00 | 0.29 |  |
| IR22 | 15 | 28 | 1 | 0.00 | 0.00 | 0.00 | 0.00 | 8.00 | 0.00 | 0.72 |  |
| IR22 | 15 | 28 | 2 | 0.00 | 0.00 | 0.00 | 0.00 | 7.50 | 0.00 | 0.30 |  |
| IR22 | 15 | 28 | 3 | 0.00 | 0.00 | 0.00 | 0.00 | 8.00 | 0.00 | 0.47 |  |
| IR22 | 15 | 28 | 4 | 0.00 | 0.00 | 0.00 | 0.00 | 7.83 | 0.00 | 0.49 |  |
| IR22 | 15 | 28 | 5 | 0.00 | 0.00 | 0.00 | 0.00 | 7.78 | 0.00 | 0.42 |  |
| IR22 | 15 | 29 | 1 | 0.00 | 0.00 | 0.00 | 0.00 | 9.00 | 0.00 | 1.25 |  |
| IR22 | 15 | 29 | 2 | 0.00 | 0.00 | 0.00 | 0.00 | 3.50 | 0.00 | 0.26 |  |
| IR22 | 15 | 29 | 3 | 0.00 | 0.00 | 0.00 | 0.00 | 7.00 | 0.00 | 0.51 |  |
| IR22 | 15 | 29 | 4 | 0.00 | 0.00 | 0.00 | 0.00 | 6.50 | 0.00 | 0.67 |  |
| IR22 | 15 | 29 | 5 | 0.00 | 0.00 | 0.00 | 0.00 | 5.67 | 0.00 | 0.48 |  |
| IR22 | 15 | 30 | 1 | 0.00 | 0.00 | 0.00 | 0.00 | 8.00 | 0.00 | 0.91 |  |
| IR22 | 15 | 30 | 2 | 0.00 | 0.00 | 0.00 | 0.00 | 7.00 | 0.00 | 0.44 |  |
| IR22 | 15 | 30 | 3 | 0.00 | 0.00 | 0.00 | 0.00 | 8.50 | 0.00 | 0.70 |  |
| IR22 | 15 | 30 | 4 | 0.00 | 0.00 | 0.00 | 0.00 | 7.83 | 0.00 | 0.68 |  |
| IR22 | 15 | 30 | 5 | 0.00 | 0.00 | 0.00 | 0.00 | 7.78 | 0.00 | 0.61 |  |
| IR22 | 20 | 1 | 1 | 7.00 | 0.00 | 0.00 | 0.00 | 0.00 | 0.00 | 0.06 | 0.58 |
| IR22 | 20 | 1 | 2 | 6.00 | 0.00 | 0.00 | 0.00 | 0.00 | 0.00 | 0.05 | 0.41 |
| IR22 | 20 | 1 | 3 | 7.00 | 0.00 | 0.00 | 0.00 | 0.00 | 0.00 | 0.07 | 0.55 |
| IR22 | 20 | 1 | 4 | 9.00 | 0.00 | 0.00 | 0.00 | 0.00 | 0.00 | 0.08 | 0.50 |
| IR22 | 20 | 1 | 5 | 7.00 | 0.00 | 0.00 | 0.00 | 0.00 | 0.00 | 0.07 | 0.37 |
| IR22 | 20 | 2 | 1 | 8.00 | 0.00 | 0.00 | 0.00 | 0.00 | 0.00 | 0.11 | 0.68 |
| IR22 | 20 | 2 | 2 | 10.00 | 0.00 | 0.00 | 0.00 | 0.00 | 0.00 | 0.15 | 0.46 |
| IR22 | 20 | 2 | 3 | 9.00 | 0.00 | 0.00 | 0.00 | 0.00 | 0.00 | 0.13 | 0.56 |
| IR22 | 20 | 2 | 4 | 8.00 | 0.00 | 0.00 | 0.00 | 0.00 | 0.00 | 0.12 | 0.46 |
| IR22 | 20 | 2 | 5 | 8.50 | 0.00 | 0.00 | 0.00 | 0.00 | 0.00 | 0.13 | 0.64 |
| IR22 | 20 | 3 | 1 | 9.00 | 0.00 | 0.00 | 0.00 | 0.00 | 0.00 | 0.21 | 0.54 |
| IR22 | 20 | 3 | 2 | 10.00 | 0.00 | 0.00 | 0.00 | 0.00 | 0.00 | 0.22 | 0.61 |
| IR22 | 20 | 3 | 3 | 10.00 | 0.00 | 0.00 | 0.00 | 0.00 | 0.00 | 0.22 | 0.58 |
| IR22 | 20 | 3 | 4 | 10.00 | 0.00 | 0.00 | 0.00 | 0.00 | 0.00 | 0.21 | 0.72 |
| IR22 | 20 | 3 | 5 | 10.00 | 0.00 | 0.00 | 0.00 | 0.00 | 0.00 | 0.21 | 0.65 |
| IR22 | 20 | 4 | 1 | 0.00 | 10.00 | 0.00 | 0.00 | 0.00 | 0.00 | 0.20 | 0.49 |
| IR22 | 20 | 4 | 2 | 0.00 | 8.00 | 0.00 | 0.00 | 0.00 | 0.00 | 0.18 | 0.45 |
| IR22 | 20 | 4 | 3 | 0.00 | 9.00 | 0.00 | 0.00 | 0.00 | 0.00 | 0.18 | 0.68 |
| IR22 | 20 | 4 | 4 | 1.00 | 9.00 | 0.00 | 0.00 | 0.00 | 0.00 | 0.19 | 0.59 |
| IR22 | 20 | 4 | 5 | 3.00 | 5.50 | 0.00 | 0.00 | 0.00 | 0.00 | 0.15 | 0.52 |
| IR22 | 20 | 5 | 1 | 0.00 | 9.00 | 0.00 | 0.00 | 0.00 | 0.00 | 0.20 | 0.64 |
| IR22 | 20 | 5 | 2 | 2.00 | 7.00 | 0.00 | 0.00 | 0.00 | 0.00 | 0.20 | 0.65 |
| IR22 | 20 | 5 | 3 | 0.00 | 9.00 | 0.00 | 0.00 | 0.00 | 0.00 | 0.19 | 1.02 |
| IR22 | 20 | 5 | 4 | 1.00 | 7.00 | 0.00 | 0.00 | 0.00 | 0.00 | 0.18 | 1.24 |
| IR22 | 20 | 5 | 5 | 1.00 | 7.50 | 0.00 | 0.00 | 0.00 | 0.00 | 0.18 | 0.68 |
| IR22 | 20 | 6 | 1 | 0.00 | 10.00 | 0.00 | 0.00 | 0.00 | 0.00 | 0.34 | 0.66 |
| IR22 | 20 | 6 | 2 | 0.00 | 9.00 | 0.00 | 0.00 | 0.00 | 0.00 | 0.28 | 1.21 |
| IR22 | 20 | 6 | 3 | 0.00 | 8.00 | 0.00 | 0.00 | 0.00 | 0.00 | 0.27 | 0.77 |
| IR22 | 20 | 6 | 4 | 0.00 | 9.00 | 0.00 | 0.00 | 0.00 | 0.00 | 0.23 | 0.59 |
| IR22 | 20 | 6 | 5 | 0.00 | 7.00 | 0.00 | 0.00 | 0.00 | 0.00 | 0.24 | 0.76 |
| IR22 | 20 | 7 | 1 | 0.00 | 10.00 | 0.00 | 0.00 | 0.00 | 0.00 | 0.43 | 0.60 |
| IR22 | 20 | 7 | 2 | 0.00 | 10.00 | 0.00 | 0.00 | 0.00 | 0.00 | 0.48 | 0.94 |
| IR22 | 20 | 7 | 3 | 0.00 | 10.00 | 0.00 | 0.00 | 0.00 | 0.00 | 0.53 | 0.92 |
| IR22 | 20 | 7 | 4 | 0.00 | 10.00 | 0.00 | 0.00 | 0.00 | 0.00 | 0.48 | 0.88 |
| IR22 | 20 | 7 | 5 | 0.00 | 9.50 | 0.00 | 0.00 | 0.00 | 0.00 | 0.43 | 0.70 |
| IR22 | 20 | 8 | 1 | 0.00 | 8.00 | 0.00 | 0.00 | 0.00 | 0.00 | 0.44 | 0.86 |
| IR22 | 20 | 8 | 2 | 0.00 | 8.00 | 0.00 | 0.00 | 0.00 | 0.00 | 0.46 | 0.94 |
| IR22 | 20 | 8 | 3 | 0.00 | 2.00 | 0.00 | 0.00 | 0.00 | 0.00 | 0.37 | 1.08 |
| IR22 | 20 | 8 | 4 | 0.00 | 10.00 | 0.00 | 0.00 | 0.00 | 0.00 | 0.47 | 0.65 |
| IR22 | 20 | 8 | 5 | 0.00 | 9.00 | 0.00 | 0.00 | 0.00 | 0.00 | 0.50 | 0.80 |
| IR22 | 20 | 9 | 1 | 0.00 | 0.00 | 8.00 | 0.00 | 0.00 | 0.00 | 0.92 | 1.07 |
| IR22 | 20 | 9 | 2 | 0.00 | 0.00 | 8.00 | 0.00 | 0.00 | 0.00 | 0.98 | 0.87 |
| IR22 | 20 | 9 | 3 | 0.00 | 0.00 | 8.00 | 0.00 | 0.00 | 0.00 | 0.92 | 0.56 |
| IR22 | 20 | 9 | 4 | 0.00 | 1.00 | 9.00 | 0.00 | 0.00 | 0.00 | 0.53 | 1.13 |
| IR22 | 20 | 9 | 5 | 0.00 | 2.00 | 6.00 | 0.00 | 0.00 | 0.00 | 0.40 | 0.95 |
| IR22 | 20 | 10 | 1 | 0.00 | 0.00 | 9.00 | 1.00 | 0.00 | 0.00 | 1.33 | 0.66 |
| IR22 | 20 | 10 | 2 | 0.00 | 0.00 | 8.00 | 2.00 | 0.00 | 0.00 | 1.14 | 0.83 |
| IR22 | 20 | 10 | 3 | 0.00 | 0.00 | 4.00 | 5.00 | 0.00 | 0.00 | 1.08 | 0.60 |
| IR22 | 20 | 10 | 4 | 0.00 | 0.00 | 8.00 |  | 0.00 | 0.00 | 0.91 | 0.49 |
| IR22 | 20 | 10 | 5 | 0.00 | 0.00 | 5.50 | 3.50 | 0.00 | 0.00 | 1.05 | 0.80 |
| IR22 | 20 | 11 | 1 | 0.00 | 0.00 | 8.00 | 0.00 | 0.00 | 0.00 | 1.01 | 0.83 |
| IR22 | 20 | 11 | 2 | 0.00 | 0.00 | 7.00 | 3.00 | 0.00 | 0.00 | 1.09 | 0.67 |
| IR22 | 20 | 11 | 3 | 0.00 | 0.00 | 6.00 | 4.00 | 0.00 | 0.00 | 1.29 | 0.62 |
| IR22 | 20 | 11 | 4 | 0.00 | 0.00 | 10.00 | 0.00 | 0.00 | 0.00 | 1.19 | 0.98 |
| IR22 | 20 | 11 | 5 | 0.00 | 0.00 | 7.00 | 2.00 | 0.00 | 0.00 | 1.09 | 0.85 |
| IR22 | 20 | 12 | 1 | 0.00 | 0.00 | 2.00 | 6.00 | 0.00 | 0.00 | 0.93 | 0.90 |
| IR22 | 20 | 12 | 2 | 0.00 | 0.00 | 1.00 | 6.00 | 0.00 | 0.00 | 0.87 | 0.76 |
| IR22 | 20 | 12 | 3 | 0.00 | 0.00 | 2.00 | 8.00 | 0.00 | 0.00 | 1.23 | 0.64 |
| IR22 | 20 | 12 | 4 | 0.00 | 0.00 | 2.00 | 6.00 | 0.00 | 0.00 | 1.09 | 0.61 |
| IR22 | 20 | 12 | 5 | 0.00 | 0.00 | 0.50 | 8.50 | 0.00 | 0.00 | 1.18 | 0.63 |
| IR22 | 20 | 13 | 1 | 0.00 | 0.00 | 3.00 | 7.00 | 0.00 | 0.00 | 1.47 | 0.79 |
| IR22 | 20 | 13 | 2 | 0.00 | 0.00 | 0.00 | 10.00 | 0.00 | 0.00 | 1.81 | 0.60 |
| IR22 | 20 | 13 | 3 | 0.00 | 0.00 | 4.00 | 3.00 | 0.00 | 0.00 | 0.90 | 0.82 |
| IR22 | 20 | 13 | 4 | 0.00 | 0.00 | 1.00 | 9.00 | 0.00 | 0.00 | 1.87 | 0.50 |
| IR22 | 20 | 13 | 5 | 0.00 | 0.00 | 0.00 | 9.50 | 0.00 | 0.00 | 2.13 | 0.66 |
| IR22 | 20 | 14 | 1 | 0.00 | 0.00 | 2.00 | 5.00 | 0.00 | 0.00 | 1.46 | 0.88 |
| IR22 | 20 | 14 | 2 | 0.00 | 0.00 | 0.00 | 7.00 | 0.00 | 0.00 | 1.91 | 0.74 |
| IR22 | 20 | 14 | 3 | 0.00 | 0.00 | 0.00 | 9.00 | 1.00 | 0.00 | 3.10 | 1.27 |
| IR22 | 20 | 14 | 4 | 0.00 | 0.00 | 0.00 | 9.00 | 0.00 | 0.00 | 2.17 | 0.88 |
| IR22 | 20 | 14 | 5 | 0.00 | 0.00 | 0.50 | 8.50 | 0.00 | 0.00 | 1.90 | 0.93 |
| IR22 | 20 | 15 | 1 | 0.00 | 0.00 | 0.00 | 6.00 | 4.00 | 0.00 | 2.89 | 1.31 |
| IR22 | 20 | 15 | 2 | 0.00 | 0.00 | 0.00 | 8.00 | 2.00 | 0.00 | 2.98 | 0.84 |
| IR22 | 20 | 15 | 3 | 0.00 | 0.00 | 0.00 | 10.00 | 0.00 | 0.00 | 3.15 | 0.78 |
| IR22 | 20 | 15 | 4 | 0.00 | 0.00 | 0.00 | 9.00 | 1.00 | 0.00 | 2.67 | 0.94 |
| IR22 | 20 | 15 | 5 | 0.00 | 0.00 | 0.00 | 8.50 | 1.50 | 0.00 | 2.59 | 1.05 |
| IR22 | 25 | 1 | 1 | 3.00 | 4.00 | 2.00 | 0.00 | 0.00 | 0.00 | 0.14 |  |
| IR22 | 25 | 1 | 2 | 9.00 | 0.50 | 0.00 | 0.00 | 0.00 | 0.00 | 0.12 |  |
| IR22 | 25 | 1 | 3 | 6.00 | 1.00 | 0.00 | 0.00 | 0.00 | 0.00 | 0.10 |  |
| IR22 | 25 | 1 | 4 | 6.00 | 1.83 | 0.67 | 0.00 | 0.00 | 0.00 | 0.12 |  |
| IR22 | 25 | 1 | 5 | 7.00 | 1.11 | 0.22 | 0.00 | 0.00 | 0.00 | 0.11 |  |
| IR22 | 25 | 2 | 1 | 0.00 | 2.50 | 4.00 | 2.50 | 0.00 | 0.00 | 0.18 |  |
| IR22 | 25 | 2 | 2 | 0.00 | 5.50 | 3.00 | 0.00 | 0.00 | 0.00 | 0.18 |  |
| IR22 | 25 | 2 | 3 | 0.00 | 0.00 | 7.00 | 0.00 | 0.00 | 0.00 | 0.15 |  |
| IR22 | 25 | 2 | 4 | 0.00 | 2.67 | 4.67 | 0.83 | 0.00 | 0.00 | 0.17 |  |
| IR22 | 25 | 2 | 5 | 0.00 | 2.72 | 4.89 | 0.28 | 0.00 | 0.00 | 0.17 |  |
| IR22 | 25 | 3 | 1 | 0.00 | 1.50 | 8.50 | 0.00 | 0.00 | 0.00 | 0.23 |  |
| IR22 | 25 | 3 | 2 | 0.00 | 4.50 | 5.50 | 0.00 | 0.00 | 0.00 | 0.21 |  |
| IR22 | 25 | 3 | 3 | 0.00 | 0.50 | 6.50 | 1.50 | 0.00 | 0.00 | 0.17 |  |
| IR22 | 25 | 3 | 4 | 0.00 | 2.17 | 6.83 | 0.50 | 0.00 | 0.00 | 0.20 |  |
| IR22 | 25 | 3 | 5 | 0.00 | 2.39 | 6.28 | 0.67 | 0.00 | 0.00 | 0.19 |  |
| IR22 | 25 | 4 | 1 | 0.00 | 0.00 | 3.50 | 6.50 | 0.00 | 0.00 | 0.28 |  |
| IR22 | 25 | 4 | 2 | 0.00 | 0.00 | 4.50 | 5.00 | 0.00 | 0.00 | 0.38 |  |
| IR22 | 25 | 4 | 3 | 0.00 | 0.00 | 1.50 | 7.50 | 1.00 | 0.00 | 0.38 |  |
| IR22 | 25 | 4 | 4 | 0.00 | 0.00 | 3.17 | 6.33 | 0.33 | 0.00 | 0.35 |  |
| IR22 | 25 | 4 | 5 | 0.00 | 0.00 | 3.06 | 6.28 | 0.44 | 0.00 | 0.37 |  |
| IR22 | 25 | 5 | 1 | 0.00 | 0.00 | 0.50 | 9.00 | 0.50 | 0.00 | 0.51 |  |
| IR22 | 25 | 5 | 2 | 0.00 | 0.00 | 0.00 | 1.00 | 7.00 | 0.00 | 0.40 |  |
| IR22 | 25 | 5 | 3 | 0.00 | 0.00 | 0.00 | 4.00 | 5.50 | 0.00 | 0.44 |  |
| IR22 | 25 | 5 | 4 | 0.00 | 0.00 | 0.17 | 4.67 | 4.33 | 0.00 | 0.45 |  |
| IR22 | 25 | 5 | 5 | 0.00 | 0.00 | 0.06 | 3.22 | 5.61 | 0.00 | 0.43 |  |
| IR22 | 25 | 6 | 1 | 0.00 | 0.00 | 0.00 | 3.00 | 6.00 | 0.00 | 0.68 |  |
| IR22 | 25 | 6 | 2 | 0.00 | 0.00 | 0.00 | 0.00 | 10.00 | 0.00 | 0.34 |  |
| IR22 | 25 | 6 | 3 | 0.00 | 0.00 | 0.00 | 0.00 | 6.50 | 0.00 | 0.52 |  |
| IR22 | 25 | 6 | 4 | 0.00 | 0.00 | 0.00 | 1.00 | 7.50 | 0.00 | 0.51 |  |
| IR22 | 25 | 6 | 5 | 0.00 | 0.00 | 0.00 | 0.33 | 8.00 | 0.00 | 0.46 |  |
| IR22 | 25 | 7 | 1 | 0.00 | 0.00 | 0.00 | 0.00 | 10.00 | 0.00 | 1.14 |  |
| IR22 | 25 | 7 | 2 | 0.00 | 0.00 | 0.00 | 0.00 | 8.50 | 0.00 | 0.96 |  |
| IR22 | 25 | 7 | 3 | 0.00 | 0.00 | 0.00 | 0.00 | 9.50 | 0.00 | 1.08 |  |
| IR22 | 25 | 7 | 4 | 0.00 | 0.00 | 0.00 | 0.00 | 9.33 | 0.00 | 1.06 |  |
| IR22 | 25 | 7 | 5 | 0.00 | 0.00 | 0.00 | 0.00 | 9.11 | 0.00 | 1.04 |  |
| IR22 | 25 | 8 | 1 | 0.00 | 0.00 | 0.00 | 0.00 | 8.00 | 0.00 | 1.59 |  |
| IR22 | 25 | 8 | 2 | 0.00 | 0.00 | 0.00 | 0.00 | 9.00 | 0.00 | 1.15 |  |
| IR22 | 25 | 8 | 3 | 0.00 | 0.00 | 0.00 | 0.50 | 9.00 | 0.00 | 1.12 |  |
| IR22 | 25 | 8 | 4 | 0.00 | 0.00 | 0.00 | 0.17 | 8.67 | 0.00 | 1.29 |  |
| IR22 | 25 | 8 | 5 | 0.00 | 0.00 | 0.00 | 0.22 | 8.89 | 0.00 | 1.19 |  |
| IR22 | 25 | 9 | 1 | 0.00 | 0.00 | 0.00 | 0.00 | 9.00 | 0.00 | 2.10 |  |
| IR22 | 25 | 9 | 2 | 0.00 | 0.00 | 0.00 | 0.00 | 7.50 | 0.00 | 2.15 |  |
| IR22 | 25 | 9 | 3 | 0.00 | 0.00 | 0.00 | 0.00 | 8.50 | 0.00 | 1.78 |  |
| IR22 | 25 | 9 | 4 | 0.00 | 0.00 | 0.00 | 0.00 | 8.33 | 0.00 | 2.01 |  |
| IR22 | 25 | 9 | 5 | 0.00 | 0.00 | 0.00 | 0.00 | 8.11 | 0.00 | 1.98 |  |
| IR22 | 25 | 10 | 1 | 0.00 | 0.00 | 0.00 | 0.00 | 9.00 | 0.00 | 2.58 |  |
| IR22 | 25 | 10 | 2 | 0.00 | 0.00 | 0.00 | 0.00 | 9.50 | 0.00 | 2.46 |  |
| IR22 | 25 | 10 | 3 | 0.00 | 0.00 | 0.00 | 0.00 | 7.50 | 0.00 | 1.97 |  |
| IR22 | 25 | 10 | 4 | 0.00 | 0.00 | 0.00 | 0.00 | 8.67 | 0.00 | 2.33 |  |
| IR22 | 25 | 10 | 5 | 0.00 | 0.00 | 0.00 | 0.00 | 8.56 | 0.00 | 2.25 |  |
| IR22 | 25 | 11 | 1 | 0.00 | 0.00 | 0.00 | 0.00 | 8.50 | 0.00 | 2.78 |  |
| IR22 | 25 | 11 | 2 | 0.00 | 0.00 | 0.00 | 0.00 | 10.00 | 0.00 | 3.11 |  |
| IR22 | 25 | 11 | 3 | 0.00 | 0.00 | 0.00 | 0.00 | 10.00 | 0.00 | 3.10 |  |
| IR22 | 25 | 11 | 4 | 0.00 | 0.00 | 0.00 | 0.00 | 9.50 | 0.00 | 3.00 |  |
| IR22 | 25 | 11 | 5 | 0.00 | 0.00 | 0.00 | 0.00 | 9.83 | 0.00 | 3.07 |  |
| IR22 | 25 | 12 | 1 | 0.00 | 0.00 | 0.00 | 0.00 | 9.00 | 0.00 | 4.02 |  |
| IR22 | 25 | 12 | 2 | 0.00 | 0.00 | 0.00 | 0.00 | 8.00 | 0.00 | 3.91 |  |
| IR22 | 25 | 12 | 3 | 0.00 | 0.00 | 0.00 | 0.00 | 8.00 | 1.00 | 4.54 |  |
| IR22 | 25 | 12 | 4 | 0.00 | 0.00 | 0.00 | 0.00 | 8.33 | 0.33 | 4.15 |  |
| IR22 | 25 | 12 | 5 | 0.00 | 0.00 | 0.00 | 0.00 | 8.11 | 0.44 | 4.20 |  |
| IR22 | 25 | 13 | 1 | 0.00 | 0.00 | 0.00 | 0.00 | 9.00 | 0.50 | 5.30 |  |
| IR22 | 25 | 13 | 2 | 0.00 | 0.00 | 0.00 | 0.00 | 7.50 | 1.00 | 5.46 |  |
| IR22 | 25 | 13 | 3 | 0.00 | 0.00 | 0.00 | 0.00 | 8.50 | 1.50 | 5.22 |  |
| IR22 | 25 | 13 | 4 | 0.00 | 0.00 | 0.00 | 0.00 | 8.33 | 1.00 | 5.33 |  |
| IR22 | 25 | 13 | 5 | 0.00 | 0.00 | 0.00 | 0.00 | 8.11 | 1.17 | 5.34 |  |
| IR22 | 25 | 14 | 1 | 0.00 | 0.00 | 0.00 | 0.00 | 6.50 | 3.00 | 6.13 |  |
| IR22 | 25 | 14 | 2 | 0.00 | 0.00 | 0.00 | 0.00 | 4.00 | 4.50 | 5.72 |  |
| IR22 | 25 | 14 | 3 | 0.00 | 0.00 | 0.00 | 0.00 | 1.00 | 4.50 | 3.93 |  |
| IR22 | 25 | 14 | 4 | 0.00 | 0.00 | 0.00 | 0.00 | 3.83 | 4.00 | 5.26 |  |
| IR22 | 25 | 14 | 5 | 0.00 | 0.00 | 0.00 | 0.00 | 2.94 | 4.33 | 4.97 |  |
| IR22 | 25 | 15 | 1 | 0.00 | 0.00 | 0.00 | 0.00 | 1.00 | 9.00 | 6.90 |  |
| IR22 | 25 | 15 | 2 | 0.00 | 0.00 | 0.00 | 0.00 | 2.00 | 7.00 | 5.67 |  |
| IR22 | 25 | 15 | 3 | 0.00 | 0.00 | 0.00 | 0.00 | 0.50 | 9.00 | 5.99 |  |
| IR22 | 25 | 15 | 4 | 0.00 | 0.00 | 0.00 | 0.00 | 1.17 | 8.33 | 6.18 |  |
| IR22 | 25 | 15 | 5 | 0.00 | 0.00 | 0.00 | 0.00 | 1.22 | 8.11 | 5.95 |  |
| IR22 | 30 | 1 | 1 | 3.50 | 4.50 | 2.00 | 0.00 | 0.00 | 0.00 | 0.17 |  |
| IR22 | 30 | 1 | 2 | 7.00 | 2.50 | 0.00 | 0.00 | 0.00 | 0.00 | 0.14 |  |
| IR22 | 30 | 1 | 3 | 5.00 | 1.50 | 0.00 | 0.00 | 0.00 | 0.00 | 0.10 |  |
| IR22 | 30 | 1 | 4 | 5.17 | 2.83 | 0.67 | 0.00 | 0.00 | 0.00 | 0.14 |  |
| IR22 | 30 | 1 | 5 | 5.72 | 2.28 | 0.22 | 0.00 | 0.00 | 0.00 | 0.13 |  |
| IR22 | 30 | 2 | 1 | 0.50 | 6.50 | 3.00 | 0.00 | 0.00 | 0.00 | 0.24 |  |
| IR22 | 30 | 2 | 2 | 0.00 | 0.50 | 9.50 | 0.00 | 0.00 | 0.00 | 0.21 |  |
| IR22 | 30 | 2 | 3 | 0.00 | 0.00 | 6.00 | 0.50 | 0.00 | 0.00 | 0.13 |  |
| IR22 | 30 | 2 | 4 | 0.17 | 2.33 | 6.17 | 0.17 | 0.00 | 0.00 | 0.19 |  |
| IR22 | 30 | 2 | 5 | 0.06 | 0.94 | 7.22 | 0.22 | 0.00 | 0.00 | 0.18 |  |
| IR22 | 30 | 3 | 1 | 0.00 | 0.00 | 2.50 | 7.00 | 0.00 | 0.00 | 0.41 |  |
| IR22 | 30 | 3 | 2 | 0.00 | 0.00 | 4.00 | 5.00 | 0.50 | 0.00 | 0.31 |  |
| IR22 | 30 | 3 | 3 | 0.00 | 0.00 | 2.00 | 7.00 | 0.00 | 0.00 | 0.28 |  |
| IR22 | 30 | 3 | 4 | 0.00 | 0.00 | 2.83 | 6.33 | 0.17 | 0.00 | 0.33 |  |
| IR22 | 30 | 3 | 5 | 0.00 | 0.00 | 2.94 | 6.11 | 0.22 | 0.00 | 0.31 |  |
| IR22 | 30 | 4 | 1 | 0.00 | 0.00 | 0.00 | 6.50 | 3.50 | 0.00 | 0.47 |  |
| IR22 | 30 | 4 | 2 | 0.00 | 0.00 | 0.00 | 4.00 | 6.00 | 0.00 | 0.47 |  |
| IR22 | 30 | 4 | 3 | 0.00 | 0.00 | 0.00 | 4.50 | 1.50 | 0.00 | 0.30 |  |
| IR22 | 30 | 4 | 4 | 0.00 | 0.00 | 0.00 | 5.00 | 3.67 | 0.00 | 0.41 |  |
| IR22 | 30 | 4 | 5 | 0.00 | 0.00 | 0.00 | 4.50 | 3.72 | 0.00 | 0.40 |  |
| IR22 | 30 | 5 | 1 | 0.00 | 0.00 | 0.00 | 3.50 | 6.00 | 0.00 | 0.58 |  |
| IR22 | 30 | 5 | 2 | 0.00 | 0.00 | 0.00 | 0.00 | 9.50 | 0.00 | 0.59 |  |
| IR22 | 30 | 5 | 3 | 0.00 | 0.00 | 0.00 | 1.00 | 8.50 | 0.00 | 0.55 |  |
| IR22 | 30 | 5 | 4 | 0.00 | 0.00 | 0.00 | 1.50 | 8.00 | 0.00 | 0.57 |  |
| IR22 | 30 | 5 | 5 | 0.00 | 0.00 | 0.00 | 0.83 | 8.67 | 0.00 | 0.57 |  |
| IR22 | 30 | 6 | 1 | 0.00 | 0.00 | 0.00 | 1.00 | 9.00 | 0.00 | 1.06 |  |
| IR22 | 30 | 6 | 2 | 0.00 | 0.00 | 0.00 | 0.00 | 8.00 | 0.00 | 0.81 |  |
| IR22 | 30 | 6 | 3 | 0.00 | 0.00 | 0.00 | 0.00 | 6.50 | 0.00 | 0.66 |  |
| IR22 | 30 | 6 | 4 | 0.00 | 0.00 | 0.00 | 0.33 | 7.83 | 0.00 | 0.84 |  |
| IR22 | 30 | 6 | 5 | 0.00 | 0.00 | 0.00 | 0.11 | 7.44 | 0.00 | 0.77 |  |
| IR22 | 30 | 7 | 1 | 0.00 | 0.00 | 1.00 | 0.50 | 8.50 | 0.00 | 1.04 |  |
| IR22 | 30 | 7 | 2 | 0.00 | 0.00 | 0.00 | 0.00 | 9.50 | 0.00 | 1.29 |  |
| IR22 | 30 | 7 | 3 | 0.00 | 0.00 | 0.00 | 0.00 | 9.50 | 0.00 | 1.07 |  |
| IR22 | 30 | 7 | 4 | 0.00 | 0.00 | 0.33 | 0.17 | 9.17 | 0.00 | 1.13 |  |
| IR22 | 30 | 7 | 5 | 0.00 | 0.00 | 0.11 | 0.06 | 9.39 | 0.00 | 1.16 |  |
| IR22 | 30 | 8 | 1 | 0.00 | 0.00 | 0.00 | 0.50 | 9.00 | 0.00 | 1.45 |  |
| IR22 | 30 | 8 | 2 | 0.00 | 0.00 | 0.00 | 0.00 | 8.50 | 0.00 | 1.53 |  |
| IR22 | 30 | 8 | 3 | 0.00 | 0.00 | 0.00 | 0.00 | 9.00 | 0.00 | 1.24 |  |
| IR22 | 30 | 8 | 4 | 0.00 | 0.00 | 0.00 | 0.17 | 8.83 | 0.00 | 1.41 |  |
| IR22 | 30 | 8 | 5 | 0.00 | 0.00 | 0.00 | 0.06 | 8.78 | 0.00 | 1.39 |  |
| IR22 | 30 | 9 | 1 | 0.00 | 0.00 | 0.00 | 0.00 | 10.00 | 0.00 | 1.88 |  |
| IR22 | 30 | 9 | 2 | 0.00 | 0.00 | 0.00 | 0.00 | 8.50 | 0.00 | 2.05 |  |
| IR22 | 30 | 9 | 3 | 0.00 | 0.00 | 0.00 | 0.00 | 7.00 | 0.00 | 1.40 |  |
| IR22 | 30 | 9 | 4 | 0.00 | 0.00 | 0.00 | 0.00 | 8.50 | 0.00 | 1.77 |  |
| IR22 | 30 | 9 | 5 | 0.00 | 0.00 | 0.00 | 0.00 | 8.00 | 0.00 | 1.74 |  |
| IR22 | 30 | 10 | 1 | 0.00 | 0.00 | 0.00 | 0.00 | 10.00 | 0.00 | 2.40 |  |
| IR22 | 30 | 10 | 2 | 0.00 | 0.00 | 0.00 | 0.00 | 10.00 | 0.00 | 2.67 |  |
| IR22 | 30 | 10 | 3 | 0.00 | 0.00 | 0.00 | 0.00 | 10.00 | 0.00 | 2.13 |  |
| IR22 | 30 | 10 | 4 | 0.00 | 0.00 | 0.00 | 0.00 | 10.00 | 0.00 | 2.40 |  |
| IR22 | 30 | 10 | 5 | 0.00 | 0.00 | 0.00 | 0.00 | 10.00 | 0.00 | 2.40 |  |
| IR22 | 30 | 11 | 1 | 0.00 | 0.00 | 0.00 | 0.00 | 10.00 | 0.00 | 2.23 |  |
| IR22 | 30 | 11 | 2 | 0.00 | 0.00 | 0.00 | 0.00 | 9.50 | 0.00 | 3.05 |  |
| IR22 | 30 | 11 | 3 | 0.00 | 0.00 | 0.00 | 0.00 | 10.00 | 0.00 | 3.18 |  |
| IR22 | 30 | 11 | 4 | 0.00 | 0.00 | 0.00 | 0.00 | 9.83 | 0.00 | 2.82 |  |
| IR22 | 30 | 11 | 5 | 0.00 | 0.00 | 0.00 | 0.00 | 9.78 | 0.00 | 3.01 |  |
| IR22 | 30 | 12 | 1 | 0.00 | 0.00 | 0.00 | 0.00 | 6.50 | 3.50 | 3.89 |  |
| IR22 | 30 | 12 | 2 | 0.00 | 0.00 | 0.00 | 0.00 | 9.00 | 1.00 | 3.76 |  |
| IR22 | 30 | 12 | 3 | 0.00 | 0.00 | 0.00 | 0.00 | 8.50 | 1.00 | 3.22 |  |
| IR22 | 30 | 12 | 4 | 0.00 | 0.00 | 0.00 | 0.00 | 8.00 | 1.83 | 3.62 |  |
| IR22 | 30 | 12 | 5 | 0.00 | 0.00 | 0.00 | 0.00 | 8.50 | 1.28 | 3.53 |  |
| IR22 | 30 | 13 | 1 | 0.00 | 0.00 | 0.00 | 0.00 | 7.50 | 2.50 | 2.91 |  |
| IR22 | 30 | 13 | 2 | 0.00 | 0.00 | 0.00 | 0.00 | 5.00 | 5.00 | 4.48 |  |
| IR22 | 30 | 13 | 3 | 0.00 | 0.00 | 0.00 | 0.00 | 7.50 | 2.50 | 3.67 |  |
| IR22 | 30 | 13 | 4 | 0.00 | 0.00 | 0.00 | 0.00 | 6.67 | 3.33 | 3.69 |  |
| IR22 | 30 | 13 | 5 | 0.00 | 0.00 | 0.00 | 0.00 | 6.39 | 3.61 | 3.95 |  |
| IR22 | 30 | 14 | 1 | 0.00 | 0.00 | 0.00 | 0.00 | 7.00 | 3.00 | 4.13 |  |
| IR22 | 30 | 14 | 2 | 0.00 | 0.00 | 0.00 | 0.00 | 6.00 | 3.50 | 3.41 |  |
| IR22 | 30 | 14 | 3 | 0.00 | 0.00 | 0.00 | 0.00 | 4.50 | 5.00 | 2.31 |  |
| IR22 | 30 | 14 | 4 | 0.00 | 0.00 | 0.00 | 0.00 | 5.83 | 3.83 | 3.28 |  |
| IR22 | 30 | 14 | 5 | 0.00 | 0.00 | 0.00 | 0.00 | 5.44 | 4.11 | 3.00 |  |
| IR22 | 30 | 15 | 1 | 0.00 | 0.00 | 0.00 | 0.00 | 0.50 | 4.50 | 2.20 |  |
| IR22 | 30 | 15 | 2 | 0.00 | 0.00 | 0.00 | 0.00 | 2.50 | 7.50 | 3.91 |  |
| IR22 | 30 | 15 | 3 | 0.00 | 0.00 | 0.00 | 0.00 | 0.50 | 9.50 | 3.92 |  |
| IR22 | 30 | 15 | 4 | 0.00 | 0.00 | 0.00 | 0.00 | 1.17 | 7.17 | 3.34 |  |
| IR22 | 30 | 15 | 5 | 0.00 | 0.00 | 0.00 | 0.00 | 1.39 | 8.06 | 3.73 |  |
| IR22 | 35 | 1 | 1 | 0.00 | 1.50 | 5.00 | 3.00 | 0.00 | 0.00 | 0.16 |  |
| IR22 | 35 | 1 | 2 | 0.00 | 2.00 | 4.50 | 0.00 | 0.00 | 0.00 | 0.12 |  |
| IR22 | 35 | 1 | 3 | 1.50 | 7.50 | 0.00 | 0.00 | 0.00 | 0.00 | 0.10 |  |
| IR22 | 35 | 1 | 4 | 8.67 | 0.00 | 0.00 | 0.00 | 0.00 | 0.00 | 0.09 |  |
| IR22 | 35 | 1 | 5 | 6.00 | 0.00 | 0.00 | 0.00 | 0.00 | 0.00 | 0.07 |  |
| IR22 | 35 | 2 | 1 | 0.00 | 0.50 | 4.50 | 4.00 | 0.00 | 0.00 | 0.17 |  |
| IR22 | 35 | 2 | 2 | 0.00 | 0.50 | 6.00 | 0.00 | 0.00 | 0.00 | 0.10 |  |
| IR22 | 35 | 2 | 3 | 0.00 | 0.00 | 7.00 | 0.00 | 0.00 | 0.00 | 0.14 |  |
| IR22 | 35 | 2 | 4 | 9.67 | 0.00 | 0.00 | 0.00 | 0.00 | 0.00 | 0.12 |  |
| IR22 | 35 | 2 | 5 | 9.00 | 0.00 | 0.00 | 0.00 | 0.00 | 0.00 | 0.12 |  |
| IR22 | 35 | 3 | 1 | 0.00 | 0.00 | 5.00 | 5.00 | 0.00 | 0.00 | 0.19 |  |
| IR22 | 35 | 3 | 2 | 0.00 | 0.00 | 1.00 | 7.50 | 0.50 | 0.00 | 0.14 |  |
| IR22 | 35 | 3 | 3 | 0.00 | 0.00 | 0.00 | 4.50 | 3.50 | 0.00 | 0.19 |  |
| IR22 | 35 | 3 | 4 | 4.67 | 0.00 | 0.00 | 0.00 | 0.00 | 0.00 | 0.06 |  |
| IR22 | 35 | 3 | 5 | 7.67 | 0.00 | 0.00 | 0.00 | 0.00 | 0.00 | 0.09 |  |
| IR22 | 35 | 4 | 1 | 0.00 | 0.00 | 1.00 | 8.50 | 0.00 | 0.00 | 0.17 |  |
| IR22 | 35 | 4 | 2 | 0.00 | 0.00 | 0.00 | 0.50 | 6.00 | 0.00 | 0.17 |  |
| IR22 | 35 | 4 | 3 | 0.00 | 0.00 | 0.00 | 0.50 | 6.50 | 0.00 | 0.12 |  |
| IR22 | 35 | 4 | 4 | 5.67 | 0.00 | 0.00 | 0.00 | 0.00 | 0.00 | 0.08 |  |
| IR22 | 35 | 4 | 5 | 6.67 | 0.00 | 0.00 | 0.00 | 0.00 | 0.00 | 0.09 |  |
| IR22 | 35 | 5 | 1 | 0.00 | 0.00 | 0.00 | 8.50 | 0.50 | 0.00 | 0.28 |  |
| IR22 | 35 | 5 | 2 | 0.00 | 0.00 | 0.00 | 0.00 | 5.50 | 0.00 | 0.17 |  |
| IR22 | 35 | 5 | 3 | 0.00 | 0.00 | 0.00 | 1.00 | 5.50 | 0.00 | 0.16 |  |
| IR22 | 35 | 5 | 4 | 4.00 | 0.33 | 0.00 | 0.00 | 0.00 | 0.00 | 0.08 |  |
| IR22 | 35 | 5 | 5 | 1.33 | 0.00 | 0.00 | 0.00 | 0.00 | 0.00 | 0.02 |  |
| IR22 | 35 | 6 | 1 | 0.00 | 0.00 | 0.00 | 5.00 | 2.50 | 0.00 | 0.18 |  |
| IR22 | 35 | 6 | 2 | 0.00 | 0.00 | 0.00 | 0.00 | 8.00 | 0.00 | 0.26 |  |
| IR22 | 35 | 6 | 3 | 0.00 | 0.00 | 0.00 | 0.00 | 7.00 | 0.00 | 0.17 |  |
| IR22 | 35 | 6 | 4 | 4.00 | 0.00 | 0.00 | 0.00 | 0.00 | 0.00 | 0.07 | 0.73 |
| IR22 | 35 | 6 | 5 | 3.33 | 0.00 | 0.00 | 0.00 | 0.00 | 0.00 | 0.07 | 0.72 |
| IR22 | 35 | 7 | 1 | 0.00 | 0.00 | 1.00 | 5.50 | 1.50 | 0.00 | 0.17 |  |
| IR22 | 35 | 7 | 2 | 0.00 | 0.00 | 0.00 | 0.00 | 7.00 | 0.00 | 0.22 |  |
| IR22 | 35 | 7 | 3 | 0.00 | 0.00 | 0.00 | 0.00 | 5.50 | 0.00 | 0.09 |  |
| IR22 | 35 | 7 | 4 | 1.67 | 0.00 | 0.00 | 0.00 | 0.00 | 0.00 | 0.03 | 0.75 |
| IR22 | 35 | 7 | 5 | 4.67 | 0.00 | 0.00 | 0.00 | 0.00 | 0.00 | 0.08 | 0.76 |
| IR22 | 35 | 8 | 1 | 0.00 | 0.00 | 0.00 | 0.50 | 6.00 | 0.00 | 0.19 |  |
| IR22 | 35 | 8 | 2 | 0.00 | 0.00 | 0.00 | 0.00 | 6.50 | 0.00 | 0.23 |  |
| IR22 | 35 | 8 | 3 | 0.00 | 0.00 | 0.00 | 0.00 | 3.50 | 0.00 | 0.06 |  |
| IR22 | 35 | 8 | 4 | 0.00 | 0.00 | 0.00 | 0.00 | 0.00 | 0.00 | 0.00 | 0.75 |
| IR22 | 35 | 8 | 5 | 0.00 | 0.00 | 0.00 | 0.00 | 0.00 | 0.00 | 0.00 | 0.81 |
| IR22 | 35 | 9 | 1 | 0.00 | 0.00 | 0.00 | 0.00 | 5.50 | 0.00 | 0.15 |  |
| IR22 | 35 | 9 | 2 | 0.00 | 0.00 | 0.00 | 0.00 | 6.50 | 0.00 | 0.25 |  |
| IR22 | 35 | 9 | 3 | 0.00 | 0.00 | 0.00 | 0.00 | 3.00 | 0.00 | 0.07 |  |
| IR22 | 35 | 9 | 4 | 0.00 | 0.00 | 0.00 | 0.00 | 0.00 | 0.00 | 0.00 | 0.70 |
| IR22 | 35 | 9 | 5 | 0.00 | 0.00 | 0.00 | 0.00 | 0.00 | 0.00 | 0.00 | 0.82 |
| IR22 | 35 | 10 | 1 | 0.00 | 0.00 | 0.00 | 0.00 | 3.50 | 0.00 | 0.10 |  |
| IR22 | 35 | 10 | 2 | 0.00 | 0.00 | 0.00 | 0.00 | 6.00 | 0.00 | 0.16 |  |
| IR22 | 35 | 10 | 3 | 0.00 | 0.00 | 0.00 | 0.00 | 2.50 | 0.00 | 0.05 |  |
| IR22 | 35 | 10 | 4 | 0.00 | 0.00 | 0.00 | 0.00 | 0.00 | 0.00 | 0.00 | 0.71 |
| IR22 | 35 | 10 | 5 | 0.00 | 0.00 | 0.00 | 0.00 | 0.00 | 0.00 | 0.00 | 0.91 |
| IR22 | 35 | 11 | 1 | 0.00 | 0.00 | 0.00 | 0.00 | 2.00 | 0.00 | 0.08 |  |
| IR22 | 35 | 11 | 2 | 0.00 | 0.00 | 0.00 | 0.00 | 7.50 | 0.00 | 0.41 |  |
| IR22 | 35 | 11 | 3 | 0.00 | 0.00 | 0.00 | 0.00 | 0.00 | 0.00 | 0.00 |  |
| IR22 | 35 | 11 | 4 | 0.00 | 0.00 | 0.00 | 0.00 | 0.00 | 0.00 | 0.00 | 1.08 |
| IR22 | 35 | 11 | 5 | 0.00 | 0.00 | 0.00 | 0.00 | 0.00 | 0.00 | 0.00 | 0.95 |
| IR22 | 35 | 12 | 1 | 0.00 | 0.00 | 0.00 | 0.00 | 1.50 | 0.00 | 0.03 |  |
| IR22 | 35 | 12 | 2 | 0.00 | 0.00 | 0.00 | 0.00 | 3.50 | 0.00 | 0.21 |  |
| IR22 | 35 | 12 | 3 | 0.00 | 0.00 | 0.00 | 0.00 | 1.50 | 0.00 | 0.04 |  |
| IR22 | 35 | 12 | 4 | 0.00 | 0.00 | 0.00 | 0.00 | 0.00 | 0.00 | 0.00 | 1.07 |
| IR22 | 35 | 12 | 5 | 0.00 | 0.00 | 0.00 | 0.00 | 0.00 | 0.00 | 0.00 | 0.91 |
| IR22 | 35 | 13 | 1 | 0.00 | 0.00 | 0.00 | 0.00 | 0.00 | 0.00 | 0.00 |  |
| IR22 | 35 | 13 | 2 | 0.00 | 0.00 | 0.00 | 0.00 | 8.00 | 0.00 | 0.42 |  |
| IR22 | 35 | 13 | 3 | 0.00 | 0.00 | 0.00 | 0.00 | 1.50 | 0.00 | 0.03 |  |
| IR22 | 35 | 13 | 4 | 0.00 | 0.00 | 0.00 | 0.00 | 0.00 | 0.00 | 0.00 | 1.06 |
| IR22 | 35 | 13 | 5 | 0.00 | 0.00 | 0.00 | 0.00 | 0.00 | 0.00 | 0.00 | 0.89 |
| IR22 | 35 | 14 | 1 | 0.00 | 0.00 | 0.00 | 0.00 | 0.00 | 0.00 | 0.00 |  |
| IR22 | 35 | 14 | 2 | 0.00 | 0.00 | 0.00 | 0.00 | 5.50 | 0.00 | 0.38 |  |
| IR22 | 35 | 14 | 3 | 0.00 | 0.00 | 0.00 | 0.00 | 0.50 | 0.00 | 0.00 |  |
| IR22 | 35 | 14 | 4 | 0.00 | 0.00 | 0.00 | 0.00 | 0.00 | 0.00 | 0.00 | 1.07 |
| IR22 | 35 | 14 | 5 | 0.00 | 0.00 | 0.00 | 0.00 | 0.00 | 0.00 | 0.00 | 0.98 |
| IR22 | 35 | 15 | 1 | 0.00 | 0.00 | 0.00 | 0.00 | 1.00 | 0.00 | 0.04 |  |
| IR22 | 35 | 15 | 2 | 0.00 | 0.00 | 0.00 | 0.00 | 5.00 | 0.00 | 0.40 |  |
| IR22 | 35 | 15 | 3 | 0.00 | 0.00 | 0.00 | 0.00 | 0.50 | 0.00 | 0.01 |  |
| IR22 | 35 | 15 | 4 | 0.00 | 0.00 | 0.00 | 0.00 | 0.00 | 0.00 | 0.00 | 1.12 |
| IR22 | 35 | 15 | 5 | 0.00 | 0.00 | 0.00 | 0.00 | 0.00 | 0.00 | 0.00 | 1.07 |
| IR22 | 40 | 1 | 1 | 0.00 | 0.00 | 0.00 | 0.00 | 0.00 | 0.00 | 0.00 | 0.35 |
| IR22 | 40 | 1 | 2 | 0.00 | 0.00 | 0.00 | 0.00 | 0.00 | 0.00 | 0.00 | 0.33 |
| IR22 | 40 | 1 | 3 | 0.00 | 0.00 | 0.00 | 0.00 | 0.00 | 0.00 | 0.00 | 0.32 |
| IR22 | 40 | 1 | 4 | 0.00 | 0.00 | 0.00 | 0.00 | 0.00 | 0.00 | 0.00 | 0.37 |
| IR22 | 40 | 1 | 5 | 0.00 | 0.00 | 0.00 | 0.00 | 0.00 | 0.00 | 0.00 | 0.31 |
| IR22 | 40 | 2 | 1 | 0.00 | 0.00 | 0.00 | 0.00 | 0.00 | 0.00 | 0.00 | 0.39 |
| IR22 | 40 | 2 | 2 | 0.00 | 0.00 | 0.00 | 0.00 | 0.00 | 0.00 | 0.00 | 0.31 |
| IR22 | 40 | 2 | 3 | 0.00 | 0.00 | 0.00 | 0.00 | 0.00 | 0.00 | 0.00 | 0.34 |
| IR22 | 40 | 2 | 4 | 0.00 | 0.00 | 0.00 | 0.00 | 0.00 | 0.00 | 0.00 | 0.32 |
| IR22 | 40 | 2 | 5 | 0.00 | 0.00 | 0.00 | 0.00 | 0.00 | 0.00 | 0.00 | 0.38 |
| IR22 | 40 | 3 | 1 | 0.00 | 0.00 | 0.00 | 0.00 | 0.00 | 0.00 | 0.00 | 0.36 |
| IR22 | 40 | 3 | 2 | 0.00 | 0.00 | 0.00 | 0.00 | 0.00 | 0.00 | 0.00 | 0.41 |
| IR22 | 40 | 3 | 3 | 0.00 | 0.00 | 0.00 | 0.00 | 0.00 | 0.00 | 0.00 | 0.41 |
| IR22 | 40 | 3 | 4 | 0.00 | 0.00 | 0.00 | 0.00 | 0.00 | 0.00 | 0.00 | 0.37 |
| IR22 | 40 | 3 | 5 | 0.00 | 0.00 | 0.00 | 0.00 | 0.00 | 0.00 | 0.00 | 0.35 |
| IR22 | 40 | 4 | 1 | 0.00 | 0.00 | 0.00 | 0.00 | 0.00 | 0.00 | 0.00 | 0.40 |
| IR22 | 40 | 4 | 2 | 0.00 | 0.00 | 0.00 | 0.00 | 0.00 | 0.00 | 0.00 | 0.35 |
| IR22 | 40 | 4 | 3 | 0.00 | 0.00 | 0.00 | 0.00 | 0.00 | 0.00 | 0.00 | 0.30 |
| IR22 | 40 | 4 | 4 | 0.00 | 0.00 | 0.00 | 0.00 | 0.00 | 0.00 | 0.00 | 0.39 |
| IR22 | 40 | 4 | 5 | 0.00 | 0.00 | 0.00 | 0.00 | 0.00 | 0.00 | 0.00 | 0.41 |
| IR22 | 40 | 5 | 1 | 0.00 | 0.00 | 0.00 | 0.00 | 0.00 | 0.00 | 0.00 | 0.53 |
| IR22 | 40 | 5 | 2 | 0.00 | 0.00 | 0.00 | 0.00 | 0.00 | 0.00 | 0.00 | 0.49 |
| IR22 | 40 | 5 | 3 | 0.00 | 0.00 | 0.00 | 0.00 | 0.00 | 0.00 | 0.00 | 0.36 |
| IR22 | 40 | 5 | 4 | 0.00 | 0.00 | 0.00 | 0.00 | 0.00 | 0.00 | 0.00 | 0.38 |
| IR22 | 40 | 5 | 5 | 0.00 | 0.00 | 0.00 | 0.00 | 0.00 | 0.00 | 0.00 | 0.44 |
| IR22 | 40 | 6 | 1 | 0.00 | 0.00 | 0.00 | 0.00 | 0.00 | 0.00 | 0.00 | 0.48 |
| IR22 | 40 | 6 | 2 | 0.00 | 0.00 | 0.00 | 0.00 | 0.00 | 0.00 | 0.00 | 0.43 |
| IR22 | 40 | 6 | 3 | 0.00 | 0.00 | 0.00 | 0.00 | 0.00 | 0.00 | 0.00 | 0.43 |
| IR22 | 40 | 6 | 4 | 0.00 | 0.00 | 0.00 | 0.00 | 0.00 | 0.00 | 0.00 | 0.51 |
| IR22 | 40 | 6 | 5 | 0.00 | 0.00 | 0.00 | 0.00 | 0.00 | 0.00 | 0.00 | 0.55 |
| IR22 | 40 | 7 | 1 | 0.00 | 0.00 | 0.00 | 0.00 | 0.00 | 0.00 | 0.00 | 0.35 |
| IR22 | 40 | 7 | 2 | 0.00 | 0.00 | 0.00 | 0.00 | 0.00 | 0.00 | 0.00 | 0.56 |
| IR22 | 40 | 7 | 3 | 0.00 | 0.00 | 0.00 | 0.00 | 0.00 | 0.00 | 0.00 | 0.51 |
| IR22 | 40 | 7 | 4 | 0.00 | 0.00 | 0.00 | 0.00 | 0.00 | 0.00 | 0.00 | 0.55 |
| IR22 | 40 | 7 | 5 | 0.00 | 0.00 | 0.00 | 0.00 | 0.00 | 0.00 | 0.00 | 0.40 |
| IR22 | 40 | 8 | 1 | 0.00 | 0.00 | 0.00 | 0.00 | 0.00 | 0.00 | 0.00 | 0.48 |
| IR22 | 40 | 8 | 2 | 0.00 | 0.00 | 0.00 | 0.00 | 0.00 | 0.00 | 0.00 | 0.52 |
| IR22 | 40 | 8 | 3 | 0.00 | 0.00 | 0.00 | 0.00 | 0.00 | 0.00 | 0.00 | 0.40 |
| IR22 | 40 | 8 | 4 | 0.00 | 0.00 | 0.00 | 0.00 | 0.00 | 0.00 | 0.00 | 0.39 |
| IR22 | 40 | 8 | 5 | 0.00 | 0.00 | 0.00 | 0.00 | 0.00 | 0.00 | 0.00 | 0.55 |
| IR22 | 40 | 9 | 1 | 0.00 | 0.00 | 0.00 | 0.00 | 0.00 | 0.00 | 0.00 | 0.54 |
| IR22 | 40 | 9 | 2 | 0.00 | 0.00 | 0.00 | 0.00 | 0.00 | 0.00 | 0.00 | 0.53 |
| IR22 | 40 | 9 | 3 | 0.00 | 0.00 | 0.00 | 0.00 | 0.00 | 0.00 | 0.00 | 0.47 |
| IR22 | 40 | 9 | 4 | 0.00 | 0.00 | 0.00 | 0.00 | 0.00 | 0.00 | 0.00 | 0.31 |
| IR22 | 40 | 9 | 5 | 0.00 | 0.00 | 0.00 | 0.00 | 0.00 | 0.00 | 0.00 | 0.40 |
| IR22 | 40 | 10 | 1 | 0.00 | 0.00 | 0.00 | 0.00 | 0.00 | 0.00 | 0.00 | 0.51 |
| IR22 | 40 | 10 | 2 | 0.00 | 0.00 | 0.00 | 0.00 | 0.00 | 0.00 | 0.00 | 0.50 |
| IR22 | 40 | 10 | 3 | 0.00 | 0.00 | 0.00 | 0.00 | 0.00 | 0.00 | 0.00 | 0.49 |
| IR22 | 40 | 10 | 4 | 0.00 | 0.00 | 0.00 | 0.00 | 0.00 | 0.00 | 0.00 | 0.56 |
| IR22 | 40 | 10 | 5 | 0.00 | 0.00 | 0.00 | 0.00 | 0.00 | 0.00 | 0.00 | 0.59 |
| IR22 | 40 | 11 | 1 | 0.00 | 0.00 | 0.00 | 0.00 | 0.00 | 0.00 | 0.00 | 0.65 |
| IR22 | 40 | 11 | 2 | 0.00 | 0.00 | 0.00 | 0.00 | 0.00 | 0.00 | 0.00 | 0.56 |
| IR22 | 40 | 11 | 3 | 0.00 | 0.00 | 0.00 | 0.00 | 0.00 | 0.00 | 0.00 | 0.47 |
| IR22 | 40 | 11 | 4 | 0.00 | 0.00 | 0.00 | 0.00 | 0.00 | 0.00 | 0.00 | 0.63 |
| IR22 | 40 | 11 | 5 | 0.00 | 0.00 | 0.00 | 0.00 | 0.00 | 0.00 | 0.00 | 0.60 |
| IR22 | 40 | 12 | 1 | 0.00 | 0.00 | 0.00 | 0.00 | 0.00 | 0.00 | 0.00 | 0.52 |
| IR22 | 40 | 12 | 2 | 0.00 | 0.00 | 0.00 | 0.00 | 0.00 | 0.00 | 0.00 | 0.56 |
| IR22 | 40 | 12 | 3 | 0.00 | 0.00 | 0.00 | 0.00 | 0.00 | 0.00 | 0.00 | 0.46 |
| IR22 | 40 | 12 | 4 | 0.00 | 0.00 | 0.00 | 0.00 | 0.00 | 0.00 | 0.00 | 0.49 |
| IR22 | 40 | 12 | 5 | 0.00 | 0.00 | 0.00 | 0.00 | 0.00 | 0.00 | 0.00 | 0.61 |
| IR22 | 40 | 13 | 1 | 0.00 | 0.00 | 0.00 | 0.00 | 0.00 | 0.00 | 0.00 | 0.55 |
| IR22 | 40 | 13 | 2 | 0.00 | 0.00 | 0.00 | 0.00 | 0.00 | 0.00 | 0.00 | 0.59 |
| IR22 | 40 | 13 | 3 | 0.00 | 0.00 | 0.00 | 0.00 | 0.00 | 0.00 | 0.00 | 0.41 |
| IR22 | 40 | 13 | 4 | 0.00 | 0.00 | 0.00 | 0.00 | 0.00 | 0.00 | 0.00 | 0.55 |
| IR22 | 40 | 13 | 5 | 0.00 | 0.00 | 0.00 | 0.00 | 0.00 | 0.00 | 0.00 | 0.55 |
| IR22 | 40 | 14 | 1 | 0.00 | 0.00 | 0.00 | 0.00 | 0.00 | 0.00 | 0.00 | 0.48 |
| IR22 | 40 | 14 | 2 | 0.00 | 0.00 | 0.00 | 0.00 | 0.00 | 0.00 | 0.00 | 0.59 |
| IR22 | 40 | 14 | 3 | 0.00 | 0.00 | 0.00 | 0.00 | 0.00 | 0.00 | 0.00 | 0.68 |
| IR22 | 40 | 14 | 4 | 0.00 | 0.00 | 0.00 | 0.00 | 0.00 | 0.00 | 0.00 | 0.56 |
| IR22 | 40 | 14 | 5 | 0.00 | 0.00 | 0.00 | 0.00 | 0.00 | 0.00 | 0.00 | 0.65 |
| IR22 | 40 | 15 | 1 | 0.00 | 0.00 | 0.00 | 0.00 | 0.00 | 0.00 | 0.00 | 0.66 |
| IR22 | 40 | 15 | 2 | 0.00 | 0.00 | 0.00 | 0.00 | 0.00 | 0.00 | 0.00 | 0.74 |
| IR22 | 40 | 15 | 3 | 0.00 | 0.00 | 0.00 | 0.00 | 0.00 | 0.00 | 0.00 | 0.56 |
| IR22 | 40 | 15 | 4 | 0.00 | 0.00 | 0.00 | 0.00 | 0.00 | 0.00 | 0.00 | 0.55 |
| IR22 | 40 | 15 | 5 | 0.00 | 0.00 | 0.00 | 0.00 | 0.00 | 0.00 | 0.00 | 0.52 |
| IR62 | 15 | 1 | 1 | 0.00 | 1.50 | 3.50 | 0.00 | 0.00 | 0.00 | 0.05 |  |
| IR62 | 15 | 1 | 2 | 0.00 | 1.00 | 6.50 | 0.00 | 0.00 | 0.00 | 0.05 |  |
| IR62 | 15 | 1 | 3 | 4.50 | 2.00 | 0.50 | 0.00 | 0.00 | 0.00 | 0.05 |  |
| IR62 | 15 | 1 | 4 | 7.67 | 0.00 | 0.00 | 0.00 | 0.00 | 0.00 | 0.06 | 0.33 |
| IR62 | 15 | 1 | 5 | 5.67 | 0.00 | 0.00 | 0.00 | 0.00 | 0.00 | 0.04 | 0.28 |
| IR62 | 15 | 2 | 1 | 2.00 | 5.00 | 0.00 | 0.00 | 0.00 | 0.00 | 0.05 |  |
| IR62 | 15 | 2 | 2 | 0.00 | 0.00 | 4.50 | 0.00 | 0.00 | 0.00 | 0.04 |  |
| IR62 | 15 | 2 | 3 | 0.00 | 2.50 | 4.50 | 0.00 | 0.00 | 0.00 | 0.05 |  |
| IR62 | 15 | 2 | 4 | 7.33 | 0.00 | 0.00 | 0.00 | 0.00 | 0.00 | 0.10 | 0.34 |
| IR62 | 15 | 2 | 5 | 8.00 | 0.00 | 0.00 | 0.00 | 0.00 | 0.00 | 0.12 | 0.41 |
| IR62 | 15 | 3 | 1 | 2.00 | 3.50 | 3.50 | 0.00 | 0.00 | 0.00 | 0.08 |  |
| IR62 | 15 | 3 | 2 | 0.00 | 0.00 | 1.50 | 6.00 | 0.00 | 0.00 | 0.06 |  |
| IR62 | 15 | 3 | 3 | 0.00 | 0.50 | 6.00 | 0.50 | 0.00 | 0.00 | 0.06 |  |
| IR62 | 15 | 3 | 4 | 7.00 | 0.00 | 0.00 | 0.00 | 0.00 | 0.00 | 0.08 | 0.32 |
| IR62 | 15 | 3 | 5 | 8.00 | 0.00 | 0.00 | 0.00 | 0.00 | 0.00 | 0.09 | 0.37 |
| IR62 | 15 | 4 | 1 | 1.00 | 0.50 | 4.00 | 3.00 | 0.00 | 0.00 | 0.11 |  |
| IR62 | 15 | 4 | 2 | 0.00 | 0.00 | 0.50 | 2.50 | 2.50 | 0.00 | 0.06 |  |
| IR62 | 15 | 4 | 3 | 0.00 | 0.00 | 3.00 | 2.50 | 0.00 | 0.00 | 0.06 |  |
| IR62 | 15 | 4 | 4 | 8.33 | 0.00 | 0.00 | 0.00 | 0.00 | 0.00 | 0.12 | 0.43 |
| IR62 | 15 | 4 | 5 | 9.33 | 0.00 | 0.00 | 0.00 | 0.00 | 0.00 | 0.12 | 0.44 |
| IR62 | 15 | 5 | 1 | 0.00 | 0.50 | 2.50 | 4.00 | 0.00 | 0.00 | 0.16 |  |
| IR62 | 15 | 5 | 2 | 0.00 | 0.00 | 0.00 | 1.50 | 3.50 | 0.00 | 0.07 |  |
| IR62 | 15 | 5 | 3 | 0.00 | 0.00 | 2.50 | 4.50 | 0.00 | 0.00 | 0.07 |  |
| IR62 | 15 | 5 | 4 | 8.33 | 0.00 | 0.00 | 0.00 | 0.00 | 0.00 | 0.12 | 0.44 |
| IR62 | 15 | 5 | 5 | 7.33 | 0.00 | 0.00 | 0.00 | 0.00 | 0.00 | 0.13 | 0.39 |
| IR62 | 15 | 6 | 1 | 0.00 | 0.00 | 1.50 | 7.00 | 0.50 | 0.00 | 0.19 |  |
| IR62 | 15 | 6 | 2 | 0.00 | 0.00 | 0.50 | 0.50 | 6.50 | 0.00 | 0.12 |  |
| IR62 | 15 | 6 | 3 | 0.00 | 0.00 | 0.00 | 1.50 | 7.50 | 0.00 | 0.14 |  |
| IR62 | 15 | 6 | 4 | 7.33 | 0.00 | 0.00 | 0.00 | 0.00 | 0.00 | 0.15 | 0.51 |
| IR62 | 15 | 6 | 5 | 8.00 | 0.00 | 0.00 | 0.00 | 0.00 | 0.00 | 0.23 | 0.54 |
| IR62 | 15 | 7 | 1 | 0.00 | 0.00 | 0.00 | 1.00 | 5.00 | 0.00 | 0.11 |  |
| IR62 | 15 | 7 | 2 | 0.00 | 0.00 | 0.50 | 0.00 | 5.50 | 0.00 | 0.10 |  |
| IR62 | 15 | 7 | 3 | 0.00 | 0.00 | 1.00 | 1.00 | 4.00 | 0.00 | 0.06 |  |
| IR62 | 15 | 7 | 4 | 7.67 | 0.00 | 0.00 | 0.00 | 0.00 | 0.00 | 0.16 | 0.53 |
| IR62 | 15 | 7 | 5 | 9.00 | 0.00 | 0.00 | 0.00 | 0.00 | 0.00 | 0.18 | 0.41 |
| IR62 | 15 | 8 | 1 | 0.00 | 0.00 | 0.00 | 1.00 | 5.50 | 0.00 | 0.14 |  |
| IR62 | 15 | 8 | 2 | 0.00 | 0.00 | 0.00 | 0.00 | 5.50 | 0.00 | 0.12 |  |
| IR62 | 15 | 8 | 3 | 0.00 | 0.00 | 0.00 | 0.50 | 7.00 | 0.00 | 0.11 |  |
| IR62 | 15 | 8 | 4 | 7.00 | 0.00 | 0.00 | 0.00 | 0.00 | 0.00 | 0.15 | 0.52 |
| IR62 | 15 | 8 | 5 | 8.33 | 0.00 | 0.00 | 0.00 | 0.00 | 0.00 | 0.17 | 0.52 |
| IR62 | 15 | 9 | 1 | 0.00 | 0.00 | 0.00 | 0.50 | 7.00 | 0.00 | 0.15 |  |
| IR62 | 15 | 9 | 2 | 0.00 | 0.00 | 0.00 | 0.00 | 6.00 | 0.00 | 0.11 |  |
| IR62 | 15 | 9 | 3 | 0.00 | 0.00 | 0.00 | 0.00 | 6.00 | 0.00 | 0.10 |  |
| IR62 | 15 | 9 | 4 | 6.67 | 0.67 | 0.00 | 0.00 | 0.00 | 0.00 | 0.17 | 0.58 |
| IR62 | 15 | 9 | 5 | 6.33 | 1.33 | 0.00 | 0.00 | 0.00 | 0.00 | 0.18 | 0.54 |
| IR62 | 15 | 10 | 1 | 0.00 | 0.00 | 0.00 | 0.50 | 9.00 | 0.00 | 0.18 |  |
| IR62 | 15 | 10 | 2 | 0.00 | 0.00 | 0.00 | 0.00 | 8.50 | 0.00 | 0.16 |  |
| IR62 | 15 | 10 | 3 | 0.00 | 0.00 | 0.00 | 0.50 | 7.00 | 0.00 | 0.12 |  |
| IR62 | 15 | 10 | 4 | 2.00 | 5.00 | 0.00 | 0.00 | 0.00 | 0.00 | 0.15 | 0.73 |
| IR62 | 15 | 10 | 5 | 4.00 | 3.33 | 0.00 | 0.00 | 0.00 | 0.00 | 0.18 | 0.65 |
| IR62 | 15 | 11 | 1 | 0.00 | 0.00 | 0.00 | 0.00 | 8.00 | 0.00 | 0.20 |  |
| IR62 | 15 | 11 | 2 | 0.00 | 0.00 | 0.00 | 0.00 | 7.00 | 0.00 | 0.14 |  |
| IR62 | 15 | 11 | 3 | 0.00 | 0.00 | 0.00 | 0.00 | 6.50 | 0.00 | 0.13 |  |
| IR62 | 15 | 11 | 4 | 1.67 | 7.33 | 0.00 | 0.00 | 0.00 | 0.00 | 0.28 | 0.54 |
| IR62 | 15 | 11 | 5 | 1.00 | 6.33 | 0.00 | 0.00 | 0.00 | 0.00 | 0.23 | 0.50 |
| IR62 | 15 | 12 | 1 | 0.00 | 0.00 | 0.00 | 0.00 | 9.50 | 0.00 | 0.18 |  |
| IR62 | 15 | 12 | 2 | 0.00 | 0.00 | 0.00 | 0.00 | 8.50 | 0.00 | 0.15 |  |
| IR62 | 15 | 12 | 3 | 0.00 | 0.00 | 0.00 | 0.00 | 2.50 | 0.00 | 0.05 |  |
| IR62 | 15 | 12 | 4 | 0.00 | 8.33 | 0.00 | 0.00 | 0.00 | 0.00 | 0.31 | 0.60 |
| IR62 | 15 | 12 | 5 | 0.33 | 8.67 | 0.00 | 0.00 | 0.00 | 0.00 | 0.36 | 0.70 |
| IR62 | 15 | 13 | 1 | 0.00 | 0.00 | 0.00 | 0.00 | 9.50 | 0.00 | 0.26 |  |
| IR62 | 15 | 13 | 2 | 0.00 | 0.00 | 0.00 | 0.00 | 7.00 | 0.00 | 0.12 |  |
| IR62 | 15 | 13 | 3 | 0.00 | 0.00 | 0.00 | 0.00 | 8.00 | 0.00 | 0.14 |  |
| IR62 | 15 | 13 | 4 | 0.00 | 8.33 | 0.00 | 0.00 | 0.00 | 0.00 | 0.45 | 0.67 |
| IR62 | 15 | 13 | 5 | 0.00 | 9.33 | 0.00 | 0.00 | 0.00 | 0.00 | 0.39 | 0.57 |
| IR62 | 15 | 14 | 1 | 0.00 | 0.00 | 0.00 | 0.50 | 7.50 | 0.00 | 0.33 |  |
| IR62 | 15 | 14 | 2 | 0.00 | 0.00 | 0.00 | 0.00 | 4.50 | 0.00 | 0.08 |  |
| IR62 | 15 | 14 | 3 | 0.00 | 0.00 | 0.00 | 0.00 | 8.50 | 0.00 | 0.22 |  |
| IR62 | 15 | 14 | 4 | 0.00 | 6.67 | 2.00 | 0.00 | 0.00 | 0.00 | 0.43 | 0.77 |
| IR62 | 15 | 14 | 5 | 0.00 | 8.33 | 0.33 | 0.00 | 0.00 | 0.00 | 0.43 | 0.61 |
| IR62 | 15 | 15 | 1 | 0.00 | 0.00 | 0.00 | 0.00 | 7.50 | 0.00 | 0.25 |  |
| IR62 | 15 | 15 | 2 | 0.00 | 0.00 | 0.00 | 0.00 | 6.00 | 0.00 | 0.12 |  |
| IR62 | 15 | 15 | 3 | 0.00 | 0.00 | 0.00 | 0.00 | 8.50 | 0.00 | 0.20 |  |
| IR62 | 15 | 15 | 4 | 0.00 | 2.67 | 4.33 | 0.00 | 0.00 | 0.00 | 0.36 | 0.58 |
| IR62 | 15 | 15 | 5 | 0.00 | 3.67 | 5.00 | 0.67 | 0.00 | 0.00 | 0.48 | 0.77 |
| IR62 | 15 | 16 | 1 | 0.00 | 0.00 | 0.00 | 0.00 | 8.50 | 0.00 | 0.36 |  |
| IR62 | 15 | 16 | 2 | 0.00 | 0.00 | 0.00 | 0.00 | 8.50 | 0.00 | 0.21 |  |
| IR62 | 15 | 16 | 3 | 0.00 | 0.00 | 0.00 | 0.00 | 7.50 | 0.00 | 0.22 |  |
| IR62 | 15 | 16 | 4 | 0.00 | 0.00 | 0.00 | 0.00 | 8.17 | 0.00 | 0.26 |  |
| IR62 | 15 | 16 | 5 | 0.00 | 0.00 | 0.00 | 0.00 | 8.06 | 0.00 | 0.23 |  |
| IR62 | 15 | 17 | 1 | 0.00 | 0.00 | 0.00 | 0.00 | 7.50 | 0.00 | 0.43 |  |
| IR62 | 15 | 17 | 2 | 0.00 | 0.00 | 0.00 | 0.00 | 7.00 | 0.00 | 0.16 |  |
| IR62 | 15 | 17 | 3 | 0.00 | 0.00 | 0.00 | 0.50 | 5.50 | 0.00 | 0.14 |  |
| IR62 | 15 | 17 | 4 | 0.00 | 0.00 | 0.00 | 0.17 | 6.67 | 0.00 | 0.24 |  |
| IR62 | 15 | 17 | 5 | 0.00 | 0.00 | 0.00 | 0.22 | 6.39 | 0.00 | 0.18 |  |
| IR62 | 15 | 18 | 1 | 0.00 | 0.00 | 0.00 | 0.00 | 6.50 | 0.00 | 0.27 |  |
| IR62 | 15 | 18 | 2 | 0.00 | 0.00 | 0.00 | 0.00 | 7.00 | 0.00 | 0.17 |  |
| IR62 | 15 | 18 | 3 | 0.00 | 0.00 | 0.00 | 0.50 | 7.00 | 0.00 | 0.21 |  |
| IR62 | 15 | 18 | 4 | 0.00 | 0.00 | 0.00 | 0.17 | 6.83 | 0.00 | 0.22 |  |
| IR62 | 15 | 18 | 5 | 0.00 | 0.00 | 0.00 | 0.22 | 6.94 | 0.00 | 0.20 |  |
| IR62 | 15 | 19 | 1 | 0.00 | 0.00 | 0.00 | 0.00 | 9.00 | 0.00 | 0.23 |  |
| IR62 | 15 | 19 | 2 | 0.00 | 0.00 | 0.00 | 0.00 | 8.50 | 0.00 | 0.24 |  |
| IR62 | 15 | 19 | 3 | 0.00 | 0.00 | 0.00 | 2.00 | 6.00 | 0.00 | 0.21 |  |
| IR62 | 15 | 19 | 4 | 0.00 | 0.00 | 0.00 | 0.67 | 7.83 | 0.00 | 0.23 |  |
| IR62 | 15 | 19 | 5 | 0.00 | 0.00 | 0.00 | 0.89 | 7.44 | 0.00 | 0.23 |  |
| IR62 | 15 | 20 | 1 | 0.00 | 0.00 | 0.00 | 0.00 | 7.50 | 0.00 | 0.38 |  |
| IR62 | 15 | 20 | 2 | 0.00 | 0.00 | 0.00 | 0.00 | 6.00 | 0.00 | 0.17 |  |
| IR62 | 15 | 20 | 3 | 0.00 | 0.00 | 0.00 | 0.00 | 8.00 | 0.00 | 0.29 |  |
| IR62 | 15 | 20 | 4 | 0.00 | 0.00 | 0.00 | 0.00 | 7.17 | 0.00 | 0.28 |  |
| IR62 | 15 | 20 | 5 | 0.00 | 0.00 | 0.00 | 0.00 | 7.06 | 0.00 | 0.25 |  |
| IR62 | 15 | 21 | 1 | 0.00 | 0.00 | 0.00 | 0.00 | 9.00 | 0.00 | 0.58 |  |
| IR62 | 15 | 21 | 2 | 0.00 | 0.00 | 0.00 | 0.00 | 7.00 | 0.00 | 0.21 |  |
| IR62 | 15 | 21 | 3 | 0.00 | 0.00 | 0.00 | 0.00 | 5.00 | 0.00 | 0.19 |  |
| IR62 | 15 | 21 | 4 | 0.00 | 0.00 | 0.00 | 0.00 | 7.00 | 0.00 | 0.33 |  |
| IR62 | 15 | 21 | 5 | 0.00 | 0.00 | 0.00 | 0.00 | 6.33 | 0.00 | 0.24 |  |
| IR62 | 15 | 22 | 1 | 0.00 | 0.00 | 0.00 | 0.00 | 7.00 | 0.00 | 0.31 |  |
| IR62 | 15 | 22 | 2 | 0.00 | 0.00 | 0.00 | 0.00 | 7.00 | 0.00 | 0.31 |  |
| IR62 | 15 | 22 | 3 | 0.00 | 0.00 | 0.00 | 0.00 | 8.00 | 0.00 | 0.34 |  |
| IR62 | 15 | 22 | 4 | 0.00 | 0.00 | 0.00 | 0.00 | 7.33 | 0.00 | 0.32 |  |
| IR62 | 15 | 22 | 5 | 0.00 | 0.00 | 0.00 | 0.00 | 7.44 | 0.00 | 0.32 |  |
| IR62 | 15 | 23 | 1 | 0.00 | 0.00 | 0.00 | 0.00 | 9.50 | 0.00 | 0.42 |  |
| IR62 | 15 | 23 | 2 | 0.00 | 0.00 | 0.00 | 0.00 | 6.50 | 0.00 | 0.24 |  |
| IR62 | 15 | 23 | 3 | 0.00 | 0.00 | 0.00 | 0.00 | 7.00 | 0.00 | 0.23 |  |
| IR62 | 15 | 23 | 4 | 0.00 | 0.00 | 0.00 | 0.00 | 7.67 | 0.00 | 0.30 |  |
| IR62 | 15 | 23 | 5 | 0.00 | 0.00 | 0.00 | 0.00 | 7.06 | 0.00 | 0.25 |  |
| IR62 | 15 | 24 | 1 | 0.00 | 0.00 | 0.00 | 0.00 | 5.00 | 0.00 | 0.42 |  |
| IR62 | 15 | 24 | 2 | 0.00 | 0.00 | 0.00 | 0.00 | 6.50 | 0.00 | 0.21 |  |
| IR62 | 15 | 24 | 3 | 0.00 | 0.00 | 0.00 | 0.00 | 4.00 | 0.00 | 0.14 |  |
| IR62 | 15 | 24 | 4 | 0.00 | 0.00 | 0.00 | 0.00 | 5.17 | 0.00 | 0.26 |  |
| IR62 | 15 | 24 | 5 | 0.00 | 0.00 | 0.00 | 0.00 | 5.22 | 0.00 | 0.20 |  |
| IR62 | 15 | 25 | 1 | 0.00 | 0.00 | 0.00 | 0.00 | 8.00 | 0.00 | 0.54 |  |
| IR62 | 15 | 25 | 2 | 0.00 | 0.00 | 0.00 | 0.00 | 7.50 | 0.00 | 0.31 |  |
| IR62 | 15 | 25 | 3 | 0.00 | 0.00 | 0.00 | 0.00 | 5.50 | 0.00 | 0.20 |  |
| IR62 | 15 | 25 | 4 | 0.00 | 0.00 | 0.00 | 0.00 | 7.00 | 0.00 | 0.35 |  |
| IR62 | 15 | 25 | 5 | 0.00 | 0.00 | 0.00 | 0.00 | 6.67 | 0.00 | 0.29 |  |
| IR62 | 15 | 26 | 1 | 0.00 | 0.00 | 0.00 | 0.00 | 7.50 | 0.00 | 0.65 |  |
| IR62 | 15 | 26 | 2 | 0.00 | 0.00 | 0.00 | 0.00 | 6.50 | 0.00 | 0.22 |  |
| IR62 | 15 | 26 | 3 | 0.00 | 0.00 | 0.00 | 0.00 | 7.00 | 0.00 | 0.33 |  |
| IR62 | 15 | 26 | 4 | 0.00 | 0.00 | 0.00 | 0.00 | 7.00 | 0.00 | 0.40 |  |
| IR62 | 15 | 26 | 5 | 0.00 | 0.00 | 0.00 | 0.00 | 6.83 | 0.00 | 0.32 |  |
| IR62 | 15 | 27 | 1 | 0.00 | 0.00 | 0.00 | 0.00 | 9.00 | 0.00 | 0.84 |  |
| IR62 | 15 | 27 | 2 | 0.00 | 0.00 | 0.00 | 0.00 | 7.50 | 0.00 | 0.29 |  |
| IR62 | 15 | 27 | 3 | 0.00 | 0.00 | 0.00 | 0.00 | 4.00 | 0.00 | 0.19 |  |
| IR62 | 15 | 27 | 4 | 0.00 | 0.00 | 0.00 | 0.00 | 6.83 | 0.00 | 0.44 |  |
| IR62 | 15 | 27 | 5 | 0.00 | 0.00 | 0.00 | 0.00 | 6.11 | 0.00 | 0.31 |  |
| IR62 | 15 | 28 | 1 | 0.00 | 0.00 | 0.00 | 0.00 | 8.50 | 0.00 | 0.88 |  |
| IR62 | 15 | 28 | 2 | 0.00 | 0.00 | 0.00 | 0.00 | 8.00 | 0.00 | 0.37 |  |
| IR62 | 15 | 28 | 3 | 0.00 | 0.00 | 0.00 | 0.00 | 6.00 | 0.00 | 0.27 |  |
| IR62 | 15 | 28 | 4 | 0.00 | 0.00 | 0.00 | 0.00 | 7.50 | 0.00 | 0.51 |  |
| IR62 | 15 | 28 | 5 | 0.00 | 0.00 | 0.00 | 0.00 | 7.17 | 0.00 | 0.38 |  |
| IR62 | 15 | 29 | 1 | 0.00 | 0.00 | 0.00 | 0.00 | 7.00 | 0.00 | 0.72 |  |
| IR62 | 15 | 29 | 2 | 0.00 | 0.00 | 0.00 | 0.00 | 9.00 | 0.00 | 0.49 |  |
| IR62 | 15 | 29 | 3 | 0.00 | 0.00 | 0.00 | 0.00 | 6.50 | 0.00 | 0.37 |  |
| IR62 | 15 | 29 | 4 | 0.00 | 0.00 | 0.00 | 0.00 | 7.50 | 0.00 | 0.53 |  |
| IR62 | 15 | 29 | 5 | 0.00 | 0.00 | 0.00 | 0.00 | 7.67 | 0.00 | 0.46 |  |
| IR62 | 15 | 30 | 1 | 0.00 | 0.00 | 0.00 | 0.00 | 6.50 | 0.00 | 0.76 |  |
| IR62 | 15 | 30 | 2 | 0.00 | 0.00 | 0.00 | 0.00 | 8.00 | 0.00 | 0.43 |  |
| IR62 | 15 | 30 | 3 | 0.00 | 0.00 | 0.00 | 0.00 | 8.00 | 0.00 | 0.40 |  |
| IR62 | 15 | 30 | 4 | 0.00 | 0.00 | 0.00 | 0.00 | 7.50 | 0.00 | 0.53 |  |
| IR62 | 15 | 30 | 5 | 0.00 | 0.00 | 0.00 | 0.00 | 7.83 | 0.00 | 0.45 |  |
| IR62 | 20 | 1 | 1 | 8.00 | 0.00 | 0.00 | 0.00 | 0.00 | 0.00 | 0.08 | 0.34 |
| IR62 | 20 | 1 | 2 | 5.00 | 0.00 | 0.00 | 0.00 | 0.00 | 0.00 | 0.04 | 0.38 |
| IR62 | 20 | 1 | 3 | 7.00 | 0.00 | 0.00 | 0.00 | 0.00 | 0.00 | 0.06 | 0.28 |
| IR62 | 20 | 1 | 4 | 10.00 | 0.00 | 0.00 | 0.00 | 0.00 | 0.00 | 0.10 | 0.48 |
| IR62 | 20 | 1 | 5 | 6.00 | 0.00 | 0.00 | 0.00 | 0.00 | 0.00 | 0.05 | 0.41 |
| IR62 | 20 | 2 | 1 | 7.00 | 0.00 | 0.00 | 0.00 | 0.00 | 0.00 | 0.09 | 0.45 |
| IR62 | 20 | 2 | 2 | 8.00 | 0.00 | 0.00 | 0.00 | 0.00 | 0.00 | 0.09 | 0.33 |
| IR62 | 20 | 2 | 3 | 8.00 | 0.00 | 0.00 | 0.00 | 0.00 | 0.00 | 0.11 | 0.36 |
| IR62 | 20 | 2 | 4 | 8.00 | 0.00 | 0.00 | 0.00 | 0.00 | 0.00 | 0.10 | 0.45 |
| IR62 | 20 | 2 | 5 | 8.00 | 0.00 | 0.00 | 0.00 | 0.00 | 0.00 | 0.10 | 0.39 |
| IR62 | 20 | 3 | 1 | 8.00 | 0.00 | 0.00 | 0.00 | 0.00 | 0.00 | 0.16 | 0.55 |
| IR62 | 20 | 3 | 2 | 8.00 | 0.00 | 0.00 | 0.00 | 0.00 | 0.00 | 0.15 | 0.31 |
| IR62 | 20 | 3 | 3 | 8.00 | 0.00 | 0.00 | 0.00 | 0.00 | 0.00 | 0.16 | 0.42 |
| IR62 | 20 | 3 | 4 | 7.00 | 0.00 | 0.00 | 0.00 | 0.00 | 0.00 | 0.12 | 0.64 |
| IR62 | 20 | 3 | 5 | 7.00 | 0.00 | 0.00 | 0.00 | 0.00 | 0.00 | 0.12 | 0.34 |
| IR62 | 20 | 4 | 1 | 4.00 | 4.00 | 0.00 | 0.00 | 0.00 | 0.00 | 0.15 | 0.55 |
| IR62 | 20 | 4 | 2 | 5.00 | 3.00 | 0.00 | 0.00 | 0.00 | 0.00 | 0.10 | 0.49 |
| IR62 | 20 | 4 | 3 | 6.00 | 2.00 | 0.00 | 0.00 | 0.00 | 0.00 | 0.14 | 0.64 |
| IR62 | 20 | 4 | 4 | 3.00 | 4.00 | 0.00 | 0.00 | 0.00 | 0.00 | 0.13 | 0.62 |
| IR62 | 20 | 4 | 5 | 6.00 | 2.00 | 0.00 | 0.00 | 0.00 | 0.00 | 0.15 | 0.56 |
| IR62 | 20 | 5 | 1 | 6.00 | 4.00 | 0.00 | 0.00 | 0.00 | 0.00 | 0.22 | 0.49 |
| IR62 | 20 | 5 | 2 | 3.00 | 5.00 | 0.00 | 0.00 | 0.00 | 0.00 | 0.16 | 0.83 |
| IR62 | 20 | 5 | 3 | 5.00 | 4.00 | 0.00 | 0.00 | 0.00 | 0.00 | 0.18 | 0.70 |
| IR62 | 20 | 5 | 4 | 5.00 | 5.00 | 0.00 | 0.00 | 0.00 | 0.00 | 0.20 | 0.74 |
| IR62 | 20 | 5 | 5 | 3.50 | 4.50 | 0.00 | 0.00 | 0.00 | 0.00 | 0.16 | 0.61 |
| IR62 | 20 | 6 | 1 | 0.00 | 8.00 | 0.00 | 0.00 | 0.00 | 0.00 | 0.30 | 0.76 |
| IR62 | 20 | 6 | 2 | 0.00 | 9.00 | 0.00 | 0.00 | 0.00 | 0.00 | 0.20 | 0.60 |
| IR62 | 20 | 6 | 3 | 0.00 | 10.00 | 0.00 | 0.00 | 0.00 | 0.00 | 0.24 | 0.73 |
| IR62 | 20 | 6 | 4 | 0.00 | 8.00 | 0.00 | 0.00 | 0.00 | 0.00 | 0.22 | 0.75 |
| IR62 | 20 | 6 | 5 | 0.00 | 8.00 | 0.00 | 0.00 | 0.00 | 0.00 | 0.17 | 0.68 |
| IR62 | 20 | 7 | 1 | 0.00 | 8.00 | 0.00 | 0.00 | 0.00 | 0.00 | 0.28 | 0.57 |
| IR62 | 20 | 7 | 2 | 0.00 | 10.00 | 0.00 | 0.00 | 0.00 | 0.00 | 0.21 | 0.52 |
| IR62 | 20 | 7 | 3 | 0.00 | 7.00 | 0.00 | 0.00 | 0.00 | 0.00 | 0.22 | 0.48 |
| IR62 | 20 | 7 | 4 | 0.00 | 8.00 | 0.00 | 0.00 | 0.00 | 0.00 | 0.31 | 0.41 |
| IR62 | 20 | 7 | 5 | 0.00 | 10.00 | 0.00 | 0.00 | 0.00 | 0.00 | 0.31 | 0.56 |
| IR62 | 20 | 8 | 1 | 0.00 | 10.00 | 0.00 | 0.00 | 0.00 | 0.00 | 0.51 | 0.48 |
| IR62 | 20 | 8 | 2 | 0.00 | 9.00 | 1.00 | 0.00 | 0.00 | 0.00 | 0.48 | 0.89 |
| IR62 | 20 | 8 | 3 | 0.00 | 8.00 | 1.00 | 0.00 | 0.00 | 0.00 | 0.32 | 0.74 |
| IR62 | 20 | 8 | 4 | 0.00 | 10.00 | 0.00 | 0.00 | 0.00 | 0.00 | 0.47 | 0.57 |
| IR62 | 20 | 8 | 5 | 0.00 | 9.50 | 0.00 | 0.00 | 0.00 | 0.00 | 0.43 | 0.47 |
| IR62 | 20 | 9 | 1 | 0.00 | 0.00 | 9.00 | 0.00 | 0.00 | 0.00 | 0.99 | 0.79 |
| IR62 | 20 | 9 | 2 | 0.00 | 0.00 | 10.00 | 0.00 | 0.00 | 0.00 | 0.69 | 0.88 |
| IR62 | 20 | 9 | 3 | 0.00 | 1.00 | 8.00 | 0.00 | 0.00 | 0.00 | 0.69 | 0.74 |
| IR62 | 20 | 9 | 4 | 0.00 | 3.00 | 4.00 | 0.00 | 0.00 | 0.00 | 0.34 | 0.70 |
| IR62 | 20 | 9 | 5 | 0.00 | 6.00 | 2.50 | 0.00 | 0.00 | 0.00 | 0.43 | 0.69 |
| IR62 | 20 | 10 | 1 | 0.00 | 0.00 | 8.00 | 0.00 | 0.00 | 0.00 | 0.64 | 0.48 |
| IR62 | 20 | 10 | 2 | 0.00 | 0.00 | 9.00 | 0.00 | 0.00 | 0.00 | 0.75 | 0.55 |
| IR62 | 20 | 10 | 3 | 0.00 | 0.00 | 5.00 | 0.00 | 0.00 | 0.00 | 0.28 | 0.76 |
| IR62 | 20 | 10 | 4 | 0.00 | 0.00 | 9.00 | 0.00 | 0.00 | 0.00 | 0.80 | 0.59 |
| IR62 | 20 | 10 | 5 | 0.00 | 0.50 | 8.50 | 0.50 | 0.00 | 0.00 | 0.90 | 0.62 |
| IR62 | 20 | 11 | 1 | 0.00 | 0.00 | 10.00 | 0.00 | 0.00 | 0.00 | 1.23 | 0.43 |
| IR62 | 20 | 11 | 2 | 0.00 | 0.00 | 5.00 | 4.00 | 0.00 | 0.00 | 1.18 | 0.80 |
| IR62 | 20 | 11 | 3 | 0.00 | 0.00 | 7.00 | 1.00 | 0.00 | 0.00 | 0.89 | 0.85 |
| IR62 | 20 | 11 | 4 | 0.00 | 0.00 | 9.00 | 0.00 | 0.00 | 0.00 | 1.19 | 0.58 |
| IR62 | 20 | 11 | 5 | 0.00 | 0.00 | 6.00 | 1.00 | 0.00 | 0.00 | 0.76 | 0.66 |
| IR62 | 20 | 12 | 1 | 0.00 | 0.00 | 7.00 | 1.00 | 0.00 | 0.00 | 0.73 | 0.67 |
| IR62 | 20 | 12 | 2 | 0.00 | 0.00 | 6.00 | 4.00 | 0.00 | 0.00 | 0.85 | 0.58 |
| IR62 | 20 | 12 | 3 | 0.00 | 0.00 | 3.00 | 7.00 | 0.00 | 0.00 | 0.98 | 0.51 |
| IR62 | 20 | 12 | 4 | 0.00 | 0.00 | 2.00 | 6.00 | 0.00 | 0.00 | 1.03 | 0.87 |
| IR62 | 20 | 12 | 5 | 0.00 | 0.00 | 3.50 | 4.00 | 0.00 | 0.00 | 0.90 | 0.69 |
| IR62 | 20 | 13 | 1 | 0.00 | 0.00 | 5.00 | 4.00 | 0.00 | 0.00 | 1.03 | 0.73 |
| IR62 | 20 | 13 | 2 | 0.00 | 0.00 | 1.00 | 8.00 | 0.00 | 0.00 | 1.84 | 0.57 |
| IR62 | 20 | 13 | 3 | 0.00 | 0.00 | 2.00 | 7.00 | 0.00 | 0.00 | 1.15 | 0.66 |
| IR62 | 20 | 13 | 4 | 0.00 | 0.00 | 1.00 | 8.00 | 0.00 | 0.00 | 1.49 | 0.73 |
| IR62 | 20 | 13 | 5 | 0.00 | 0.00 | 1.50 | 6.50 | 0.00 | 0.00 | 1.03 | 1.27 |
| IR62 | 20 | 14 | 1 | 0.00 | 0.00 | 2.00 | 6.00 | 0.00 | 0.00 | 1.50 | 0.76 |
| IR62 | 20 | 14 | 2 | 0.00 | 0.00 | 0.00 | 8.00 | 0.00 | 0.00 | 1.91 | 0.51 |
| IR62 | 20 | 14 | 3 | 0.00 | 0.00 | 1.00 | 6.00 | 0.00 | 0.00 | 0.83 | 0.61 |
| IR62 | 20 | 14 | 4 | 0.00 | 0.00 | 3.00 | 5.00 | 0.00 | 0.00 | 1.37 | 0.61 |
| IR62 | 20 | 14 | 5 | 0.00 | 0.00 | 3.00 | 6.50 | 0.00 | 0.00 | 1.77 | 0.65 |
| IR62 | 20 | 15 | 1 | 0.00 | 0.00 | 0.00 | 4.00 | 6.00 | 0.00 | 3.71 | 0.98 |
| IR62 | 20 | 15 | 2 | 0.00 | 0.00 | 1.00 | 6.00 | 1.00 | 0.00 | 1.52 | 0.70 |
| IR62 | 20 | 15 | 3 | 0.00 | 0.00 | 0.00 | 6.00 | 2.00 | 0.00 | 2.15 | 0.69 |
| IR62 | 20 | 15 | 4 | 0.00 | 0.00 | 0.00 | 9.00 | 1.00 | 0.00 | 2.75 | 0.96 |
| IR62 | 20 | 15 | 5 | 0.00 | 0.00 | 2.00 | 7.00 | 0.00 | 0.00 | 1.27 | 0.74 |
| IR62 | 25 | 1 | 1 | 6.00 | 3.50 | 0.00 | 0.00 | 0.00 | 0.00 | 0.12 |  |
| IR62 | 25 | 1 | 2 | 8.00 | 0.00 | 0.00 | 0.00 | 0.00 | 0.00 | 0.08 |  |
| IR62 | 25 | 1 | 3 | 6.00 | 0.50 | 0.00 | 0.00 | 0.00 | 0.00 | 0.07 |  |
| IR62 | 25 | 1 | 4 | 6.67 | 1.33 | 0.00 | 0.00 | 0.00 | 0.00 | 0.09 |  |
| IR62 | 25 | 1 | 5 | 6.89 | 0.61 | 0.00 | 0.00 | 0.00 | 0.00 | 0.08 |  |
| IR62 | 25 | 2 | 1 | 2.50 | 4.00 | 2.00 | 0.00 | 0.00 | 0.00 | 0.14 |  |
| IR62 | 25 | 2 | 2 | 0.50 | 5.50 | 3.50 | 0.00 | 0.00 | 0.00 | 0.17 |  |
| IR62 | 25 | 2 | 3 | 1.00 | 1.00 | 6.50 | 0.00 | 0.00 | 0.00 | 0.14 |  |
| IR62 | 25 | 2 | 4 | 1.33 | 3.50 | 4.00 | 0.00 | 0.00 | 0.00 | 0.15 |  |
| IR62 | 25 | 2 | 5 | 0.94 | 3.33 | 4.67 | 0.00 | 0.00 | 0.00 | 0.15 |  |
| IR62 | 25 | 3 | 1 | 0.00 | 2.50 | 5.00 | 2.50 | 0.00 | 0.00 | 0.30 |  |
| IR62 | 25 | 3 | 2 | 0.00 | 0.50 | 6.00 | 2.00 | 0.00 | 0.00 | 0.18 |  |
| IR62 | 25 | 3 | 3 | 0.00 | 0.00 | 3.00 | 4.00 | 0.00 | 0.00 | 0.15 |  |
| IR62 | 25 | 3 | 4 | 0.00 | 1.00 | 4.67 | 2.83 | 0.00 | 0.00 | 0.21 |  |
| IR62 | 25 | 3 | 5 | 0.00 | 0.50 | 4.56 | 2.94 | 0.00 | 0.00 | 0.18 |  |
| IR62 | 25 | 4 | 1 | 0.00 | 0.50 | 3.00 | 6.00 | 0.00 | 0.00 | 0.29 |  |
| IR62 | 25 | 4 | 2 | 0.00 | 0.00 | 3.00 | 3.50 | 1.50 | 0.00 | 0.23 |  |
| IR62 | 25 | 4 | 3 | 0.00 | 0.00 | 4.50 | 3.00 | 0.00 | 0.00 | 0.17 |  |
| IR62 | 25 | 4 | 4 | 0.00 | 0.17 | 3.50 | 4.17 | 0.50 | 0.00 | 0.23 |  |
| IR62 | 25 | 4 | 5 | 0.00 | 0.06 | 3.67 | 3.56 | 0.67 | 0.00 | 0.21 |  |
| IR62 | 25 | 5 | 1 | 0.00 | 0.00 | 0.50 | 4.50 | 3.50 | 0.00 | 0.45 |  |
| IR62 | 25 | 5 | 2 | 0.00 | 0.00 | 0.00 | 1.50 | 8.00 | 0.00 | 0.39 |  |
| IR62 | 25 | 5 | 3 | 0.00 | 0.00 | 0.50 | 4.50 | 4.50 | 0.00 | 0.32 |  |
| IR62 | 25 | 5 | 4 | 0.00 | 0.00 | 0.33 | 3.50 | 5.33 | 0.00 | 0.39 |  |
| IR62 | 25 | 5 | 5 | 0.00 | 0.00 | 0.28 | 3.17 | 5.94 | 0.00 | 0.37 |  |
| IR62 | 25 | 6 | 1 | 0.00 | 0.00 | 1.00 | 4.00 | 3.50 | 0.00 | 0.62 |  |
| IR62 | 25 | 6 | 2 | 0.00 | 0.00 | 0.00 | 0.50 | 7.00 | 0.00 | 0.36 |  |
| IR62 | 25 | 6 | 3 | 0.00 | 0.00 | 0.00 | 1.00 | 8.00 | 0.00 | 0.51 |  |
| IR62 | 25 | 6 | 4 | 0.00 | 0.00 | 0.33 | 1.83 | 6.17 | 0.00 | 0.50 |  |
| IR62 | 25 | 6 | 5 | 0.00 | 0.00 | 0.11 | 1.11 | 7.06 | 0.00 | 0.46 |  |
| IR62 | 25 | 7 | 1 | 0.00 | 0.00 | 0.00 | 2.00 | 8.00 | 0.00 | 0.83 |  |
| IR62 | 25 | 7 | 2 | 0.00 | 0.00 | 0.00 | 0.50 | 9.50 | 0.00 | 0.64 |  |
| IR62 | 25 | 7 | 3 | 0.00 | 0.00 | 0.00 | 0.00 | 7.50 | 0.00 | 0.39 |  |
| IR62 | 25 | 7 | 4 | 0.00 | 0.00 | 0.00 | 0.83 | 8.33 | 0.00 | 0.62 |  |
| IR62 | 25 | 7 | 5 | 0.00 | 0.00 | 0.00 | 0.44 | 8.44 | 0.00 | 0.55 |  |
| IR62 | 25 | 8 | 1 | 0.00 | 0.00 | 0.00 | 1.00 | 7.00 | 0.00 | 0.84 |  |
| IR62 | 25 | 8 | 2 | 0.00 | 0.00 | 0.00 | 0.00 | 10.00 | 0.00 | 0.92 |  |
| IR62 | 25 | 8 | 3 | 0.00 | 0.00 | 0.00 | 0.00 | 9.00 | 0.00 | 0.91 |  |
| IR62 | 25 | 8 | 4 | 0.00 | 0.00 | 0.00 | 0.33 | 8.67 | 0.00 | 0.89 |  |
| IR62 | 25 | 8 | 5 | 0.00 | 0.00 | 0.00 | 0.11 | 9.22 | 0.00 | 0.91 |  |
| IR62 | 25 | 9 | 1 | 0.00 | 0.00 | 0.00 | 0.50 | 9.00 | 0.00 | 1.61 |  |
| IR62 | 25 | 9 | 2 | 0.00 | 0.00 | 0.00 | 0.00 | 9.50 | 0.00 | 0.85 |  |
| IR62 | 25 | 9 | 3 | 0.00 | 0.00 | 0.00 | 0.00 | 6.50 | 0.00 | 0.52 |  |
| IR62 | 25 | 9 | 4 | 0.00 | 0.00 | 0.00 | 0.17 | 8.33 | 0.00 | 0.99 |  |
| IR62 | 25 | 9 | 5 | 0.00 | 0.00 | 0.00 | 0.06 | 8.11 | 0.00 | 0.78 |  |
| IR62 | 25 | 10 | 1 | 0.00 | 0.00 | 0.00 | 0.50 | 9.50 | 0.00 | 1.57 |  |
| IR62 | 25 | 10 | 2 | 0.00 | 0.00 | 0.00 | 0.00 | 10.00 | 0.00 | 1.43 |  |
| IR62 | 25 | 10 | 3 | 0.00 | 0.00 | 0.00 | 0.00 | 9.50 | 0.00 | 1.53 |  |
| IR62 | 25 | 10 | 4 | 0.00 | 0.00 | 0.00 | 0.17 | 9.67 | 0.00 | 1.51 |  |
| IR62 | 25 | 10 | 5 | 0.00 | 0.00 | 0.00 | 0.06 | 9.72 | 0.00 | 1.49 |  |
| IR62 | 25 | 11 | 1 | 0.00 | 0.00 | 0.00 | 0.50 | 9.00 | 0.00 | 1.21 |  |
| IR62 | 25 | 11 | 2 | 0.00 | 0.00 | 0.00 | 0.00 | 9.00 | 0.00 | 1.97 |  |
| IR62 | 25 | 11 | 3 | 0.00 | 0.00 | 0.00 | 0.00 | 7.50 | 0.00 | 1.04 |  |
| IR62 | 25 | 11 | 4 | 0.00 | 0.00 | 0.00 | 0.17 | 8.50 | 0.00 | 1.41 |  |
| IR62 | 25 | 11 | 5 | 0.00 | 0.00 | 0.00 | 0.06 | 8.33 | 0.00 | 1.47 |  |
| IR62 | 25 | 12 | 1 | 0.00 | 0.00 | 0.00 | 0.00 | 8.00 | 0.00 | 1.30 |  |
| IR62 | 25 | 12 | 2 | 0.00 | 0.00 | 0.00 | 0.00 | 9.00 | 0.00 | 1.21 |  |
| IR62 | 25 | 12 | 3 | 0.00 | 0.00 | 0.00 | 0.00 | 7.50 | 0.00 | 1.73 |  |
| IR62 | 25 | 12 | 4 | 0.00 | 0.00 | 0.00 | 0.00 | 8.17 | 0.00 | 1.41 |  |
| IR62 | 25 | 12 | 5 | 0.00 | 0.00 | 0.00 | 0.00 | 8.22 | 0.00 | 1.45 |  |
| IR62 | 25 | 13 | 1 | 0.00 | 0.00 | 0.00 | 0.00 | 10.00 | 0.00 | 1.85 |  |
| IR62 | 25 | 13 | 2 | 0.00 | 0.00 | 0.00 | 0.00 | 8.00 | 0.00 | 1.35 |  |
| IR62 | 25 | 13 | 3 | 0.00 | 0.00 | 0.00 | 0.00 | 10.00 | 0.00 | 2.10 |  |
| IR62 | 25 | 13 | 4 | 0.00 | 0.00 | 0.00 | 0.00 | 9.33 | 0.00 | 1.77 |  |
| IR62 | 25 | 13 | 5 | 0.00 | 0.00 | 0.00 | 0.00 | 9.11 | 0.00 | 1.74 |  |
| IR62 | 25 | 14 | 1 | 0.00 | 0.00 | 0.00 | 0.00 | 9.50 | 0.00 | 1.50 |  |
| IR62 | 25 | 14 | 2 | 0.00 | 0.00 | 0.00 | 0.00 | 9.00 | 0.00 | 1.98 |  |
| IR62 | 25 | 14 | 3 | 0.00 | 0.00 | 0.00 | 0.00 | 8.00 | 0.00 | 0.84 |  |
| IR62 | 25 | 14 | 4 | 0.00 | 0.00 | 0.00 | 0.00 | 8.83 | 0.00 | 1.44 |  |
| IR62 | 25 | 14 | 5 | 0.00 | 0.00 | 0.00 | 0.00 | 8.61 | 0.00 | 1.42 |  |
| IR62 | 25 | 15 | 1 | 0.00 | 0.00 | 0.00 | 0.00 | 5.00 | 2.00 | 2.65 |  |
| IR62 | 25 | 15 | 2 | 0.00 | 0.00 | 0.00 | 0.00 | 7.50 | 0.50 | 1.80 |  |
| IR62 | 25 | 15 | 3 | 0.00 | 0.00 | 0.00 | 0.00 | 7.50 | 0.00 | 1.54 |  |
| IR62 | 25 | 15 | 4 | 0.00 | 0.00 | 0.00 | 0.00 | 6.67 | 0.83 | 2.00 |  |
| IR62 | 25 | 15 | 5 | 0.00 | 0.00 | 0.00 | 0.00 | 7.22 | 0.44 | 1.78 |  |
| IR62 | 30 | 1 | 1 | 1.50 | 5.00 | 2.00 | 0.00 | 0.00 | 0.00 | 0.16 |  |
| IR62 | 30 | 1 | 2 | 6.50 | 3.00 | 0.00 | 0.00 | 0.00 | 0.00 | 0.12 |  |
| IR62 | 30 | 1 | 3 | 5.50 | 1.50 | 0.00 | 0.00 | 0.00 | 0.00 | 0.13 |  |
| IR62 | 30 | 1 | 4 | 4.50 | 3.17 | 0.67 | 0.00 | 0.00 | 0.00 | 0.13 |  |
| IR62 | 30 | 1 | 5 | 5.50 | 2.56 | 0.22 | 0.00 | 0.00 | 0.00 | 0.13 |  |
| IR62 | 30 | 2 | 1 | 0.50 | 1.50 | 3.50 | 2.50 | 0.00 | 0.00 | 0.17 |  |
| IR62 | 30 | 2 | 2 | 0.00 | 0.50 | 8.50 | 0.00 | 0.00 | 0.00 | 0.19 |  |
| IR62 | 30 | 2 | 3 | 0.00 | 0.50 | 6.50 | 0.00 | 0.00 | 0.00 | 0.15 |  |
| IR62 | 30 | 2 | 4 | 0.17 | 0.83 | 6.17 | 0.83 | 0.00 | 0.00 | 0.17 |  |
| IR62 | 30 | 2 | 5 | 0.06 | 0.61 | 7.06 | 0.28 | 0.00 | 0.00 | 0.17 |  |
| IR62 | 30 | 3 | 1 | 0.00 | 1.00 | 2.00 | 7.00 | 0.00 | 0.00 | 0.40 |  |
| IR62 | 30 | 3 | 2 | 0.00 | 0.00 | 2.50 | 7.00 | 0.00 | 0.00 | 0.22 |  |
| IR62 | 30 | 3 | 3 | 0.00 | 0.00 | 3.00 | 7.00 | 0.00 | 0.00 | 0.26 |  |
| IR62 | 30 | 3 | 4 | 0.00 | 0.33 | 2.50 | 7.00 | 0.00 | 0.00 | 0.29 |  |
| IR62 | 30 | 3 | 5 | 0.00 | 0.11 | 2.67 | 7.00 | 0.00 | 0.00 | 0.26 |  |
| IR62 | 30 | 4 | 1 | 0.00 | 0.00 | 2.50 | 3.50 | 3.50 | 0.00 | 0.31 |  |
| IR62 | 30 | 4 | 2 | 0.00 | 0.00 | 0.00 | 8.50 | 0.50 | 0.00 | 0.38 |  |
| IR62 | 30 | 4 | 3 | 0.00 | 0.00 | 0.00 | 5.00 | 0.00 | 0.00 | 0.20 |  |
| IR62 | 30 | 4 | 4 | 0.00 | 0.00 | 0.83 | 5.67 | 1.33 | 0.00 | 0.30 |  |
| IR62 | 30 | 4 | 5 | 0.00 | 0.00 | 0.28 | 6.39 | 0.61 | 0.00 | 0.30 |  |
| IR62 | 30 | 5 | 1 | 0.00 | 0.00 | 0.00 | 4.50 | 4.00 | 0.00 | 0.45 |  |
| IR62 | 30 | 5 | 2 | 0.00 | 0.00 | 0.00 | 4.50 | 4.50 | 0.00 | 0.45 |  |
| IR62 | 30 | 5 | 3 | 0.00 | 0.00 | 0.00 | 0.50 | 7.00 | 0.00 | 0.31 |  |
| IR62 | 30 | 5 | 4 | 0.00 | 0.00 | 0.00 | 3.17 | 5.17 | 0.00 | 0.41 |  |
| IR62 | 30 | 5 | 5 | 0.00 | 0.00 | 0.00 | 2.72 | 5.56 | 0.00 | 0.39 |  |
| IR62 | 30 | 6 | 1 | 0.00 | 0.00 | 0.00 | 1.50 | 7.50 | 0.00 | 0.56 |  |
| IR62 | 30 | 6 | 2 | 0.00 | 0.00 | 0.00 | 0.50 | 9.00 | 0.00 | 0.47 |  |
| IR62 | 30 | 6 | 3 | 0.00 | 0.00 | 0.00 | 0.50 | 9.00 | 0.00 | 0.78 |  |
| IR62 | 30 | 6 | 4 | 0.00 | 0.00 | 0.00 | 0.83 | 8.50 | 0.00 | 0.60 |  |
| IR62 | 30 | 6 | 5 | 0.00 | 0.00 | 0.00 | 0.61 | 8.83 | 0.00 | 0.62 |  |
| IR62 | 30 | 7 | 1 | 0.00 | 0.00 | 0.00 | 1.50 | 8.00 | 0.00 | 1.06 |  |
| IR62 | 30 | 7 | 2 | 0.00 | 0.00 | 0.00 | 0.00 | 9.50 | 0.00 | 0.83 |  |
| IR62 | 30 | 7 | 3 | 0.00 | 0.00 | 0.00 | 0.00 | 6.50 | 0.00 | 0.62 |  |
| IR62 | 30 | 7 | 4 | 0.00 | 0.00 | 0.00 | 0.50 | 8.00 | 0.00 | 0.84 |  |
| IR62 | 30 | 7 | 5 | 0.00 | 0.00 | 0.00 | 0.17 | 8.00 | 0.00 | 0.76 |  |
| IR62 | 30 | 8 | 1 | 0.00 | 0.00 | 0.00 | 0.00 | 9.00 | 0.00 | 0.99 |  |
| IR62 | 30 | 8 | 2 | 0.00 | 0.00 | 0.00 | 0.00 | 10.00 | 0.00 | 1.18 |  |
| IR62 | 30 | 8 | 3 | 0.00 | 0.00 | 0.00 | 0.00 | 8.50 | 0.00 | 0.87 |  |
| IR62 | 30 | 8 | 4 | 0.00 | 0.00 | 0.00 | 0.00 | 9.17 | 0.00 | 1.01 |  |
| IR62 | 30 | 8 | 5 | 0.00 | 0.00 | 0.00 | 0.00 | 9.22 | 0.00 | 1.02 |  |
| IR62 | 30 | 9 | 1 | 0.00 | 0.00 | 0.00 | 0.00 | 10.00 | 0.00 | 1.37 |  |
| IR62 | 30 | 9 | 2 | 0.00 | 0.00 | 0.00 | 0.00 | 9.50 | 0.00 | 0.97 |  |
| IR62 | 30 | 9 | 3 | 0.00 | 0.00 | 0.00 | 0.00 | 9.00 | 0.00 | 1.15 |  |
| IR62 | 30 | 9 | 4 | 0.00 | 0.00 | 0.00 | 0.00 | 9.50 | 0.00 | 1.16 |  |
| IR62 | 30 | 9 | 5 | 0.00 | 0.00 | 0.00 | 0.00 | 9.33 | 0.00 | 1.09 |  |
| IR62 | 30 | 10 | 1 | 0.00 | 0.00 | 0.00 | 0.00 | 8.50 | 0.00 | 1.51 |  |
| IR62 | 30 | 10 | 2 | 0.00 | 0.00 | 0.00 | 0.50 | 8.00 | 0.00 | 1.00 |  |
| IR62 | 30 | 10 | 3 | 0.00 | 0.00 | 0.00 | 0.00 | 8.50 | 0.00 | 1.41 |  |
| IR62 | 30 | 10 | 4 | 0.00 | 0.00 | 0.00 | 0.17 | 8.33 | 0.00 | 1.30 |  |
| IR62 | 30 | 10 | 5 | 0.00 | 0.00 | 0.00 | 0.22 | 8.28 | 0.00 | 1.24 |  |
| IR62 | 30 | 11 | 1 | 0.00 | 0.00 | 0.00 | 0.00 | 9.50 | 0.00 | 2.21 |  |
| IR62 | 30 | 11 | 2 | 0.00 | 0.00 | 0.00 | 0.00 | 8.00 | 0.00 | 1.85 |  |
| IR62 | 30 | 11 | 3 | 0.00 | 0.00 | 0.00 | 0.00 | 9.50 | 0.00 | 1.60 |  |
| IR62 | 30 | 11 | 4 | 0.00 | 0.00 | 0.00 | 0.00 | 9.00 | 0.00 | 1.88 |  |
| IR62 | 30 | 11 | 5 | 0.00 | 0.00 | 0.00 | 0.00 | 8.83 | 0.00 | 1.77 |  |
| IR62 | 30 | 12 | 1 | 0.00 | 0.00 | 0.00 | 0.00 | 10.00 | 0.00 | 2.41 |  |
| IR62 | 30 | 12 | 2 | 0.00 | 0.00 | 0.00 | 0.00 | 8.00 | 0.00 | 1.86 |  |
| IR62 | 30 | 12 | 3 | 0.00 | 0.00 | 0.00 | 0.00 | 8.50 | 0.00 | 1.67 |  |
| IR62 | 30 | 12 | 4 | 0.00 | 0.00 | 0.00 | 0.00 | 8.83 | 0.00 | 1.98 |  |
| IR62 | 30 | 12 | 5 | 0.00 | 0.00 | 0.00 | 0.00 | 8.44 | 0.00 | 1.84 |  |
| IR62 | 30 | 13 | 1 | 0.00 | 0.00 | 0.00 | 0.00 | 10.00 | 0.00 | 1.75 |  |
| IR62 | 30 | 13 | 2 | 0.00 | 0.00 | 0.00 | 0.00 | 9.50 | 0.00 | 1.58 |  |
| IR62 | 30 | 13 | 3 | 0.00 | 0.00 | 0.00 | 0.00 | 7.50 | 0.00 | 1.57 |  |
| IR62 | 30 | 13 | 4 | 0.00 | 0.00 | 0.00 | 0.00 | 9.00 | 0.00 | 1.63 |  |
| IR62 | 30 | 13 | 5 | 0.00 | 0.00 | 0.00 | 0.00 | 8.67 | 0.00 | 1.60 |  |
| IR62 | 30 | 14 | 1 | 0.00 | 0.00 | 0.00 | 0.00 | 6.00 | 3.50 | 2.88 |  |
| IR62 | 30 | 14 | 2 | 0.00 | 0.00 | 0.00 | 0.00 | 9.50 | 0.00 | 1.42 |  |
| IR62 | 30 | 14 | 3 | 0.00 | 0.00 | 0.00 | 0.00 | 8.00 | 0.00 | 1.38 |  |
| IR62 | 30 | 14 | 4 | 0.00 | 0.00 | 0.00 | 0.00 | 7.83 | 1.17 | 1.89 |  |
| IR62 | 30 | 14 | 5 | 0.00 | 0.00 | 0.00 | 0.00 | 8.44 | 0.39 | 1.56 |  |
| IR62 | 30 | 15 | 1 | 0.00 | 0.00 | 0.00 | 0.00 | 7.50 | 1.50 | 2.49 |  |
| IR62 | 30 | 15 | 2 | 0.00 | 0.00 | 0.00 | 0.00 | 7.50 | 0.00 | 1.34 |  |
| IR62 | 30 | 15 | 3 | 0.00 | 0.00 | 0.00 | 0.00 | 8.50 | 0.00 | 2.11 |  |
| IR62 | 30 | 15 | 4 | 0.00 | 0.00 | 0.00 | 0.00 | 7.83 | 0.50 | 1.98 |  |
| IR62 | 30 | 15 | 5 | 0.00 | 0.00 | 0.00 | 0.00 | 7.94 | 0.17 | 1.81 |  |
| IR62 | 35 | 1 | 1 | 1.00 | 4.00 | 5.00 | 0.00 | 0.00 | 0.00 | 0.14 |  |
| IR62 | 35 | 1 | 2 | 0.50 | 3.50 | 5.00 | 0.00 | 0.00 | 0.00 | 0.10 |  |
| IR62 | 35 | 1 | 3 | 0.50 | 3.50 | 2.00 | 0.00 | 0.00 | 0.00 | 0.06 |  |
| IR62 | 35 | 1 | 4 | 4.67 | 0.00 | 0.00 | 0.00 | 0.00 | 0.00 | 0.04 | 0.46 |
| IR62 | 35 | 1 | 5 | 4.67 | 0.00 | 0.00 | 0.00 | 0.00 | 0.00 | 0.06 | 0.46 |
| IR62 | 35 | 2 | 1 | 0.00 | 2.00 | 3.50 | 4.50 | 0.00 | 0.00 | 0.16 |  |
| IR62 | 35 | 2 | 2 | 0.00 | 1.00 | 8.50 | 0.00 | 0.00 | 0.00 | 0.14 |  |
| IR62 | 35 | 2 | 3 | 0.00 | 1.50 | 6.50 | 0.00 | 0.00 | 0.00 | 0.14 |  |
| IR62 | 35 | 2 | 4 | 6.00 | 0.00 | 0.00 | 0.00 | 0.00 | 0.00 | 0.07 | 0.48 |
| IR62 | 35 | 2 | 5 | 6.00 | 0.00 | 0.00 | 0.00 | 0.00 | 0.00 | 0.07 | 0.48 |
| IR62 | 35 | 3 | 1 | 0.00 | 0.50 | 7.00 | 1.00 | 0.00 | 0.00 | 0.16 |  |
| IR62 | 35 | 3 | 2 | 0.00 | 0.00 | 0.50 | 4.50 | 2.50 | 0.00 | 0.14 |  |
| IR62 | 35 | 3 | 3 | 0.00 | 0.00 | 2.00 | 3.50 | 3.50 | 0.00 | 0.12 |  |
| IR62 | 35 | 3 | 4 | 5.33 | 0.00 | 0.00 | 0.00 | 0.00 | 0.00 | 0.09 | 0.55 |
| IR62 | 35 | 3 | 5 | 5.00 | 0.00 | 0.00 | 0.00 | 0.00 | 0.00 | 0.07 | 0.48 |
| IR62 | 35 | 4 | 1 | 0.00 | 1.50 | 3.00 | 4.00 | 1.00 | 0.00 | 0.17 |  |
| IR62 | 35 | 4 | 2 | 0.00 | 0.00 | 0.00 | 2.50 | 6.00 | 0.00 | 0.14 |  |
| IR62 | 35 | 4 | 3 | 0.00 | 0.00 | 0.00 | 1.00 | 5.50 | 0.00 | 0.09 |  |
| IR62 | 35 | 4 | 4 | 4.33 | 0.00 | 0.00 | 0.00 | 0.00 | 0.00 | 0.06 | 0.55 |
| IR62 | 35 | 4 | 5 | 4.67 | 0.00 | 0.00 | 0.00 | 0.00 | 0.00 | 0.06 | 0.50 |
| IR62 | 35 | 5 | 1 | 0.00 | 0.00 | 0.50 | 7.00 | 2.00 | 0.00 | 0.21 |  |
| IR62 | 35 | 5 | 2 | 0.00 | 0.00 | 0.00 | 0.50 | 6.50 | 0.00 | 0.15 |  |
| IR62 | 35 | 5 | 3 | 0.00 | 0.00 | 0.00 | 1.50 | 6.50 | 0.00 | 0.16 |  |
| IR62 | 35 | 5 | 4 | 3.33 | 0.00 | 0.00 | 0.00 | 0.00 | 0.00 | 0.05 | 0.60 |
| IR62 | 35 | 5 | 5 | 4.33 | 0.00 | 0.00 | 0.00 | 0.00 | 0.00 | 0.06 | 0.61 |
| IR62 | 35 | 6 | 1 | 0.00 | 0.00 | 0.50 | 5.50 | 1.50 | 0.00 | 0.15 |  |
| IR62 | 35 | 6 | 2 | 0.00 | 0.00 | 0.00 | 0.50 | 8.00 | 0.00 | 0.23 |  |
| IR62 | 35 | 6 | 3 | 0.00 | 0.00 | 0.00 | 0.00 | 7.50 | 0.00 | 0.13 |  |
| IR62 | 35 | 6 | 4 | 0.00 | 0.00 | 0.00 | 0.00 | 0.00 | 0.00 | 0.00 | 0.64 |
| IR62 | 35 | 6 | 5 | 0.00 | 0.00 | 0.00 | 0.00 | 0.00 | 0.00 | 0.00 | 0.71 |
| IR62 | 35 | 7 | 1 | 0.00 | 0.00 | 0.00 | 4.00 | 5.50 | 0.00 | 0.22 |  |
| IR62 | 35 | 7 | 2 | 0.00 | 0.00 | 0.00 | 0.50 | 7.00 | 0.00 | 0.14 |  |
| IR62 | 35 | 7 | 3 | 0.00 | 0.00 | 0.00 | 0.00 | 4.00 | 0.00 | 0.05 |  |
| IR62 | 35 | 7 | 4 | 0.00 | 0.00 | 0.00 | 0.00 | 0.00 | 0.00 | 0.00 | 0.66 |
| IR62 | 35 | 7 | 5 | 0.00 | 0.00 | 0.00 | 0.00 | 0.00 | 0.00 | 0.00 | 0.72 |
| IR62 | 35 | 8 | 1 | 0.00 | 0.00 | 0.00 | 0.50 | 5.50 | 0.00 | 0.14 |  |
| IR62 | 35 | 8 | 2 | 0.00 | 0.00 | 0.00 | 0.00 | 8.00 | 0.00 | 0.20 |  |
| IR62 | 35 | 8 | 3 | 0.00 | 0.00 | 0.00 | 0.00 | 4.00 | 0.00 | 0.05 |  |
| IR62 | 35 | 8 | 4 | 0.00 | 0.00 | 0.00 | 0.00 | 0.00 | 0.00 | 0.00 | 0.64 |
| IR62 | 35 | 8 | 5 | 0.00 | 0.00 | 0.00 | 0.00 | 0.00 | 0.00 | 0.00 | 0.74 |
| IR62 | 35 | 9 | 1 | 0.00 | 0.00 | 0.00 | 0.00 | 6.50 | 0.00 | 0.14 |  |
| IR62 | 35 | 9 | 2 | 0.00 | 0.00 | 0.00 | 0.00 | 7.00 | 0.00 | 0.16 |  |
| IR62 | 35 | 9 | 3 | 0.00 | 0.00 | 0.00 | 0.00 | 4.50 | 0.00 | 0.06 |  |
| IR62 | 35 | 9 | 4 | 0.00 | 0.00 | 0.00 | 0.00 | 0.00 | 0.00 | 0.00 | 0.65 |
| IR62 | 35 | 9 | 5 | 0.00 | 0.00 | 0.00 | 0.00 | 0.00 | 0.00 | 0.00 | 0.68 |
| IR62 | 35 | 10 | 1 | 0.00 | 0.00 | 0.00 | 0.00 | 3.00 | 0.00 | 0.08 |  |
| IR62 | 35 | 10 | 2 | 0.00 | 0.00 | 0.00 | 0.00 | 4.50 | 0.00 | 0.12 |  |
| IR62 | 35 | 10 | 3 | 0.00 | 0.00 | 0.00 | 0.00 | 2.50 | 0.00 | 0.04 |  |
| IR62 | 35 | 10 | 4 | 0.00 | 0.00 | 0.00 | 0.00 | 0.00 | 0.00 | 0.00 | 0.69 |
| IR62 | 35 | 10 | 5 | 0.00 | 0.00 | 0.00 | 0.00 | 0.00 | 0.00 | 0.00 | 0.68 |
| IR62 | 35 | 11 | 1 | 0.00 | 0.00 | 0.00 | 0.00 | 3.50 | 0.00 | 0.11 |  |
| IR62 | 35 | 11 | 2 | 0.00 | 0.00 | 0.00 | 0.00 | 3.50 | 0.00 | 0.09 |  |
| IR62 | 35 | 11 | 3 | 0.00 | 0.00 | 0.00 | 0.00 | 1.50 | 0.00 | 0.04 |  |
| IR62 | 35 | 11 | 4 | 0.00 | 0.00 | 0.00 | 0.00 | 0.00 | 0.00 | 0.00 | 0.84 |
| IR62 | 35 | 11 | 5 | 0.00 | 0.00 | 0.00 | 0.00 | 0.00 | 0.00 | 0.00 | 0.55 |
| IR62 | 35 | 12 | 1 | 0.00 | 0.00 | 0.00 | 0.00 | 2.50 | 0.00 | 0.09 |  |
| IR62 | 35 | 12 | 2 | 0.00 | 0.00 | 0.00 | 0.00 | 8.00 | 0.00 | 0.29 |  |
| IR62 | 35 | 12 | 3 | 0.00 | 0.00 | 0.00 | 0.00 | 0.50 | 0.00 | 0.01 |  |
| IR62 | 35 | 12 | 4 | 0.00 | 0.00 | 0.00 | 0.00 | 0.00 | 0.00 | 0.00 | 0.92 |
| IR62 | 35 | 12 | 5 | 0.00 | 0.00 | 0.00 | 0.00 | 0.00 | 0.00 | 0.00 | 0.90 |
| IR62 | 35 | 13 | 1 | 0.00 | 0.00 | 0.00 | 0.00 | 0.00 | 0.00 | 0.00 |  |
| IR62 | 35 | 13 | 2 | 0.00 | 0.00 | 0.00 | 0.00 | 1.50 | 0.00 | 0.07 |  |
| IR62 | 35 | 13 | 3 | 0.00 | 0.00 | 0.00 | 0.00 | 2.00 | 0.00 | 0.04 |  |
| IR62 | 35 | 13 | 4 | 0.00 | 0.00 | 0.00 | 0.00 | 0.00 | 0.00 | 0.00 | 0.89 |
| IR62 | 35 | 13 | 5 | 0.00 | 0.00 | 0.00 | 0.00 | 0.00 | 0.00 | 0.00 | 0.89 |
| IR62 | 35 | 14 | 1 | 0.00 | 0.00 | 0.00 | 0.00 | 2.50 | 0.00 | 0.04 |  |
| IR62 | 35 | 14 | 2 | 0.00 | 0.00 | 0.00 | 0.00 | 2.00 | 0.00 | 0.08 |  |
| IR62 | 35 | 14 | 3 | 0.00 | 0.00 | 0.00 | 0.00 | 0.50 | 0.00 | 0.00 |  |
| IR62 | 35 | 14 | 4 | 0.00 | 0.00 | 0.00 | 0.00 | 0.00 | 0.00 | 0.00 | 0.87 |
| IR62 | 35 | 14 | 5 | 0.00 | 0.00 | 0.00 | 0.00 | 0.00 | 0.00 | 0.00 | 0.89 |
| IR62 | 35 | 15 | 1 | 0.00 | 0.00 | 0.00 | 0.00 | 0.50 | 0.00 | 0.01 |  |
| IR62 | 35 | 15 | 2 | 0.00 | 0.00 | 0.00 | 0.00 | 0.00 | 0.00 | 0.00 |  |
| IR62 | 35 | 15 | 3 | 0.00 | 0.00 | 0.00 | 0.00 | 0.00 | 0.00 | 0.00 |  |
| IR62 | 35 | 15 | 4 | 0.00 | 0.00 | 0.00 | 0.00 | 0.00 | 0.00 | 0.00 | 0.99 |
| IR62 | 35 | 15 | 5 | 0.00 | 0.00 | 0.00 | 0.00 | 0.00 | 0.00 | 0.00 | 0.91 |
| IR62 | 40 | 1 | 1 | 0.00 | 0.00 | 0.00 | 0.00 | 0.00 | 0.00 | 0.00 | 0.24 |
| IR62 | 40 | 1 | 2 | 0.00 | 0.00 | 0.00 | 0.00 | 0.00 | 0.00 | 0.00 | 0.27 |
| IR62 | 40 | 1 | 3 | 0.00 | 0.00 | 0.00 | 0.00 | 0.00 | 0.00 | 0.00 | 0.31 |
| IR62 | 40 | 1 | 4 | 0.00 | 0.00 | 0.00 | 0.00 | 0.00 | 0.00 | 0.00 | 0.27 |
| IR62 | 40 | 1 | 5 | 0.00 | 0.00 | 0.00 | 0.00 | 0.00 | 0.00 | 0.00 | 0.31 |
| IR62 | 40 | 2 | 1 | 0.00 | 0.00 | 0.00 | 0.00 | 0.00 | 0.00 | 0.00 | 0.39 |
| IR62 | 40 | 2 | 2 | 0.00 | 0.00 | 0.00 | 0.00 | 0.00 | 0.00 | 0.00 | 0.29 |
| IR62 | 40 | 2 | 3 | 0.00 | 0.00 | 0.00 | 0.00 | 0.00 | 0.00 | 0.00 | 0.36 |
| IR62 | 40 | 2 | 4 | 0.00 | 0.00 | 0.00 | 0.00 | 0.00 | 0.00 | 0.00 | 0.35 |
| IR62 | 40 | 2 | 5 | 0.00 | 0.00 | 0.00 | 0.00 | 0.00 | 0.00 | 0.00 | 0.17 |
| IR62 | 40 | 3 | 1 | 0.00 | 0.00 | 0.00 | 0.00 | 0.00 | 0.00 | 0.00 | 0.24 |
| IR62 | 40 | 3 | 2 | 0.00 | 0.00 | 0.00 | 0.00 | 0.00 | 0.00 | 0.00 | 0.29 |
| IR62 | 40 | 3 | 3 | 0.00 | 0.00 | 0.00 | 0.00 | 0.00 | 0.00 | 0.00 | 0.35 |
| IR62 | 40 | 3 | 4 | 0.00 | 0.00 | 0.00 | 0.00 | 0.00 | 0.00 | 0.00 | 0.32 |
| IR62 | 40 | 3 | 5 | 0.00 | 0.00 | 0.00 | 0.00 | 0.00 | 0.00 | 0.00 | 0.34 |
| IR62 | 40 | 4 | 1 | 0.00 | 0.00 | 0.00 | 0.00 | 0.00 | 0.00 | 0.00 | 0.44 |
| IR62 | 40 | 4 | 2 | 0.00 | 0.00 | 0.00 | 0.00 | 0.00 | 0.00 | 0.00 | 0.33 |
| IR62 | 40 | 4 | 3 | 0.00 | 0.00 | 0.00 | 0.00 | 0.00 | 0.00 | 0.00 | 0.40 |
| IR62 | 40 | 4 | 4 | 0.00 | 0.00 | 0.00 | 0.00 | 0.00 | 0.00 | 0.00 | 0.40 |
| IR62 | 40 | 4 | 5 | 0.00 | 0.00 | 0.00 | 0.00 | 0.00 | 0.00 | 0.00 | 0.36 |
| IR62 | 40 | 5 | 1 | 0.00 | 0.00 | 0.00 | 0.00 | 0.00 | 0.00 | 0.00 | 0.33 |
| IR62 | 40 | 5 | 2 | 0.00 | 0.00 | 0.00 | 0.00 | 0.00 | 0.00 | 0.00 | 0.36 |
| IR62 | 40 | 5 | 3 | 0.00 | 0.00 | 0.00 | 0.00 | 0.00 | 0.00 | 0.00 | 0.35 |
| IR62 | 40 | 5 | 4 | 0.00 | 0.00 | 0.00 | 0.00 | 0.00 | 0.00 | 0.00 | 0.39 |
| IR62 | 40 | 5 | 5 | 0.00 | 0.00 | 0.00 | 0.00 | 0.00 | 0.00 | 0.00 | 0.36 |
| IR62 | 40 | 6 | 1 | 0.00 | 0.00 | 0.00 | 0.00 | 0.00 | 0.00 | 0.00 | 0.40 |
| IR62 | 40 | 6 | 2 | 0.00 | 0.00 | 0.00 | 0.00 | 0.00 | 0.00 | 0.00 | 0.16 |
| IR62 | 40 | 6 | 3 | 0.00 | 0.00 | 0.00 | 0.00 | 0.00 | 0.00 | 0.00 | 0.38 |
| IR62 | 40 | 6 | 4 | 0.00 | 0.00 | 0.00 | 0.00 | 0.00 | 0.00 | 0.00 | 0.27 |
| IR62 | 40 | 6 | 5 | 0.00 | 0.00 | 0.00 | 0.00 | 0.00 | 0.00 | 0.00 | 0.43 |
| IR62 | 40 | 7 | 1 | 0.00 | 0.00 | 0.00 | 0.00 | 0.00 | 0.00 | 0.00 | 0.48 |
| IR62 | 40 | 7 | 2 | 0.00 | 0.00 | 0.00 | 0.00 | 0.00 | 0.00 | 0.00 | 0.56 |
| IR62 | 40 | 7 | 3 | 0.00 | 0.00 | 0.00 | 0.00 | 0.00 | 0.00 | 0.00 | 0.49 |
| IR62 | 40 | 7 | 4 | 0.00 | 0.00 | 0.00 | 0.00 | 0.00 | 0.00 | 0.00 | 0.35 |
| IR62 | 40 | 7 | 5 | 0.00 | 0.00 | 0.00 | 0.00 | 0.00 | 0.00 | 0.00 | 0.33 |
| IR62 | 40 | 8 | 1 | 0.00 | 0.00 | 0.00 | 0.00 | 0.00 | 0.00 | 0.00 | 0.58 |
| IR62 | 40 | 8 | 2 | 0.00 | 0.00 | 0.00 | 0.00 | 0.00 | 0.00 | 0.00 | 0.45 |
| IR62 | 40 | 8 | 3 | 0.00 | 0.00 | 0.00 | 0.00 | 0.00 | 0.00 | 0.00 | 0.43 |
| IR62 | 40 | 8 | 4 | 0.00 | 0.00 | 0.00 | 0.00 | 0.00 | 0.00 | 0.00 | 0.44 |
| IR62 | 40 | 8 | 5 | 0.00 | 0.00 | 0.00 | 0.00 | 0.00 | 0.00 | 0.00 | 0.49 |
| IR62 | 40 | 9 | 1 | 0.00 | 0.00 | 0.00 | 0.00 | 0.00 | 0.00 | 0.00 | 0.44 |
| IR62 | 40 | 9 | 2 | 0.00 | 0.00 | 0.00 | 0.00 | 0.00 | 0.00 | 0.00 | 0.39 |
| IR62 | 40 | 9 | 3 | 0.00 | 0.00 | 0.00 | 0.00 | 0.00 | 0.00 | 0.00 | 0.53 |
| IR62 | 40 | 9 | 4 | 0.00 | 0.00 | 0.00 | 0.00 | 0.00 | 0.00 | 0.00 | 0.47 |
| IR62 | 40 | 9 | 5 | 0.00 | 0.00 | 0.00 | 0.00 | 0.00 | 0.00 | 0.00 | 0.44 |
| IR62 | 40 | 10 | 1 | 0.00 | 0.00 | 0.00 | 0.00 | 0.00 | 0.00 | 0.00 | 0.48 |
| IR62 | 40 | 10 | 2 | 0.00 | 0.00 | 0.00 | 0.00 | 0.00 | 0.00 | 0.00 | 0.35 |
| IR62 | 40 | 10 | 3 | 0.00 | 0.00 | 0.00 | 0.00 | 0.00 | 0.00 | 0.00 | 0.53 |
| IR62 | 40 | 10 | 4 | 0.00 | 0.00 | 0.00 | 0.00 | 0.00 | 0.00 | 0.00 | 0.56 |
| IR62 | 40 | 10 | 5 | 0.00 | 0.00 | 0.00 | 0.00 | 0.00 | 0.00 | 0.00 | 0.58 |
| IR62 | 40 | 11 | 1 | 0.00 | 0.00 | 0.00 | 0.00 | 0.00 | 0.00 | 0.00 | 0.40 |
| IR62 | 40 | 11 | 2 | 0.00 | 0.00 | 0.00 | 0.00 | 0.00 | 0.00 | 0.00 | 0.58 |
| IR62 | 40 | 11 | 3 | 0.00 | 0.00 | 0.00 | 0.00 | 0.00 | 0.00 | 0.00 | 0.41 |
| IR62 | 40 | 11 | 4 | 0.00 | 0.00 | 0.00 | 0.00 | 0.00 | 0.00 | 0.00 | 0.58 |
| IR62 | 40 | 11 | 5 | 0.00 | 0.00 | 0.00 | 0.00 | 0.00 | 0.00 | 0.00 | 0.49 |
| IR62 | 40 | 12 | 1 | 0.00 | 0.00 | 0.00 | 0.00 | 0.00 | 0.00 | 0.00 | 0.50 |
| IR62 | 40 | 12 | 2 | 0.00 | 0.00 | 0.00 | 0.00 | 0.00 | 0.00 | 0.00 | 0.64 |
| IR62 | 40 | 12 | 3 | 0.00 | 0.00 | 0.00 | 0.00 | 0.00 | 0.00 | 0.00 | 0.47 |
| IR62 | 40 | 12 | 4 | 0.00 | 0.00 | 0.00 | 0.00 | 0.00 | 0.00 | 0.00 | 0.53 |
| IR62 | 40 | 12 | 5 | 0.00 | 0.00 | 0.00 | 0.00 | 0.00 | 0.00 | 0.00 | 0.40 |
| IR62 | 40 | 13 | 1 | 0.00 | 0.00 | 0.00 | 0.00 | 0.00 | 0.00 | 0.00 | 0.54 |
| IR62 | 40 | 13 | 2 | 0.00 | 0.00 | 0.00 | 0.00 | 0.00 | 0.00 | 0.00 | 0.45 |
| IR62 | 40 | 13 | 3 | 0.00 | 0.00 | 0.00 | 0.00 | 0.00 | 0.00 | 0.00 | 0.47 |
| IR62 | 40 | 13 | 4 | 0.00 | 0.00 | 0.00 | 0.00 | 0.00 | 0.00 | 0.00 | 0.35 |
| IR62 | 40 | 13 | 5 | 0.00 | 0.00 | 0.00 | 0.00 | 0.00 | 0.00 | 0.00 | 0.56 |
| IR62 | 40 | 14 | 1 | 0.00 | 0.00 | 0.00 | 0.00 | 0.00 | 0.00 | 0.00 | 0.48 |
| IR62 | 40 | 14 | 2 | 0.00 | 0.00 | 0.00 | 0.00 | 0.00 | 0.00 | 0.00 | 0.43 |
| IR62 | 40 | 14 | 3 | 0.00 | 0.00 | 0.00 | 0.00 | 0.00 | 0.00 | 0.00 | 0.32 |
| IR62 | 40 | 14 | 4 | 0.00 | 0.00 | 0.00 | 0.00 | 0.00 | 0.00 | 0.00 | 0.49 |
| IR62 | 40 | 14 | 5 | 0.00 | 0.00 | 0.00 | 0.00 | 0.00 | 0.00 | 0.00 | 0.49 |
| IR62 | 40 | 15 | 1 | 0.00 | 0.00 | 0.00 | 0.00 | 0.00 | 0.00 | 0.00 | 0.54 |
| IR62 | 40 | 15 | 2 | 0.00 | 0.00 | 0.00 | 0.00 | 0.00 | 0.00 | 0.00 | 0.53 |
| IR62 | 40 | 15 | 3 | 0.00 | 0.00 | 0.00 | 0.00 | 0.00 | 0.00 | 0.00 | 0.30 |
| IR62 | 40 | 15 | 4 | 0.00 | 0.00 | 0.00 | 0.00 | 0.00 | 0.00 | 0.00 | 0.43 |
| IR62 | 40 | 15 | 5 | 0.00 | 0.00 | 0.00 | 0.00 | 0.00 | 0.00 | 0.00 | 0.49 |
