# Supplementary material for: Elevated temperatures diminish the effects of a highly resistant rice variety on the brown planthopper
Source: Sci Rep. 2021 Jan 8;11:262. doi: 10.1038/s41598-020-80704-4 (PMC7794346; doi:10.1038/s41598-020-80704-4)
Supplement: Supplementary file 2 — Supplementary Information 2. [file 41598_2020_80704_MOESM2_ESM.docx]

**Elevated temperatures deminish the effects of a highly resistant rice variety on the brown planthopper**

Finbarr G. Horgan, Arriza Arida, Goli Ardestani, Maria Liberty P. Almazan

Supplementary Information

Adult survival and oviposition data

| plant | temp (degC) | day of obs'n | run | Subreps | % survival | Number of egg batches | Total number of eggs | batches per hopper | eggs per hopper | batch size | Plant weight (g) |
| --- | --- | --- | --- | --- | --- | --- | --- | --- | --- | --- | --- |
| IR22 | 15 | 1 | 1 | 5 | 100.00 | 0.40 | 2.40 | 0.13 | 0.80 | 6.00 | 0.12 |
| IR22 | 15 | 1 | 2 | 3 | 100.00 | 4.00 | 27.00 | 1.33 | 9.00 | 6.75 | 0.45 |
| IR22 | 15 | 1 | 3 | 3 | 100.00 | 9.00 | 26.00 | 3.00 | 8.67 | 2.89 | 0.27 |
| IR22 | 15 | 1 | 4 | 3 | 100.00 | 4.67 | 26.33 | 1.56 | 8.78 | 5.64 | 0.50 |
| IR22 | 15 | 2 | 1 | 5 | 100.00 | 0.40 | 0.80 | 0.13 | 0.27 | 2.00 | 0.17 |
| IR22 | 15 | 2 | 2 | 3 | 100.00 | 2.33 | 10.67 | 0.78 | 3.56 | 4.57 | 0.40 |
| IR22 | 15 | 2 | 3 | 3 | 100.00 | 2.67 | 8.00 | 0.89 | 2.67 | 3.00 | 0.24 |
| IR22 | 15 | 2 | 4 | 3 | 100.00 | 1.00 | 9.33 | 0.33 | 3.11 | 9.33 | 0.63 |
| IR22 | 15 | 3 | 1 | 5 | 100.00 | 0.20 | 0.20 | 0.07 | 0.07 | 1.00 | 0.18 |
| IR22 | 15 | 3 | 2 | 3 | 100.00 | 1.67 | 6.00 | 0.56 | 2.00 | 3.60 | 0.42 |
| IR22 | 15 | 3 | 3 | 3 | 100.00 | 4.67 | 15.00 | 1.56 | 5.00 | 3.21 | 0.23 |
| IR22 | 15 | 3 | 4 | 3 | 100.00 | 2.33 | 11.33 | 0.78 | 3.78 | 4.86 | 0.66 |
| IR22 | 15 | 4 | 1 | 5 | 100.00 | 1.00 | 2.00 | 0.33 | 0.67 | 2.00 | 0.16 |
| IR22 | 15 | 4 | 2 | 3 | 66.67 | 1.33 | 8.33 | 0.67 | 4.17 | 6.25 | 0.25 |
| IR22 | 15 | 4 | 3 | 3 | 100.00 | 1.00 | 2.00 | 0.33 | 0.67 | 2.00 | 0.27 |
| IR22 | 15 | 4 | 4 | 3 | 100.00 | 1.00 | 0.33 | 0.33 | 0.11 | 0.33 | 0.66 |
| IR22 | 15 | 5 | 1 | 5 | 100.00 | 2.00 | 7.40 | 0.67 | 2.47 | 3.70 | 0.18 |
| IR22 | 15 | 5 | 2 | 3 | 66.67 | 1.33 | 4.67 | 0.67 | 2.33 | 3.50 | 0.35 |
| IR22 | 15 | 5 | 3 | 3 | 100.00 | 1.33 | 2.00 | 0.44 | 0.67 | 1.50 | 0.26 |
| IR22 | 15 | 5 | 4 | 3 | 100.00 | 1.33 | 5.67 | 0.44 | 1.89 | 4.25 | 0.78 |
| IR22 | 15 | 6 | 1 | 5 | 100.00 | 1.80 | 4.60 | 0.60 | 1.53 | 2.56 | 0.21 |
| IR22 | 15 | 6 | 2 | 3 | 66.67 | 2.00 | 11.33 | 1.00 | 5.67 | 5.67 | 0.28 |
| IR22 | 15 | 6 | 3 | 3 | 66.67 | 0.67 | 0.67 | 0.33 | 0.33 | 1.00 | 0.21 |
| IR22 | 15 | 6 | 4 | 3 | 100.00 | 1.67 | 6.67 | 0.56 | 2.22 | 4.00 | 0.57 |
| IR22 | 15 | 7 | 1 | 5 | 80.00 | 0.60 | 1.20 | 0.25 | 0.50 | 2.00 | 0.15 |
| IR22 | 15 | 7 | 2 | 3 | 66.67 | 1.00 | 1.33 | 0.50 | 0.67 | 1.33 | 0.27 |
| IR22 | 15 | 7 | 3 | 3 | 33.33 | 0.33 | 0.00 | 0.33 | 0.00 | 0.00 | 0.11 |
| IR22 | 15 | 7 | 4 | 3 | 100.00 | 2.00 | 9.67 | 0.67 | 3.22 | 4.83 | 0.62 |
| IR22 | 15 | 8 | 1 | 3 | 66.67 | 0.67 | 0.44 | 0.33 | 0.15 | 0.44 | 0.34 |
| IR22 | 15 | 8 | 2 | 3 | 66.67 | 0.67 | 0.00 | 0.33 | 0.00 | 0.00 | 0.22 |
| IR22 | 15 | 8 | 3 | 3 | 33.33 | 0.33 | 0.00 | 0.33 | 0.00 | 0.00 | 0.83 |
| IR22 | 15 | 8 | 4 | 3 | 100.00 | 1.00 | 1.33 | 0.33 | 0.44 | 1.33 | 0.72 |
| IR22 | 15 | 9 | 1 | 3 | 55.56 | 0.89 | 4.89 | 0.52 | 3.56 | 5.53 | 0.34 |
| IR22 | 15 | 9 | 2 | 3 | 33.33 | 0.67 | 8.67 | 0.67 | 8.67 | 13.00 | 0.11 |
| IR22 | 15 | 9 | 3 | 3 | 33.33 | 0.33 | 0.00 | 0.33 | 0.00 | 0.00 | 0.12 |
| IR22 | 15 | 9 | 4 | 3 | 100.00 | 1.67 | 6.00 | 0.56 | 2.00 | 3.60 | 0.79 |
| IR22 | 15 | 10 | 1 | 3 | 55.56 | 0.56 | 0.56 | 0.33 | 0.19 | 0.56 | 0.50 |
| IR22 | 15 | 10 | 2 | 3 | 33.33 | 0.33 | 0.00 | 0.33 | 0.00 | 0.00 | 0.29 |
| IR22 | 15 | 10 | 3 | 3 | 33.33 | 0.33 | 0.00 | 0.33 | 0.00 | 0.00 | 0.12 |
| IR22 | 15 | 10 | 4 | 3 | 100.00 | 1.00 | 1.67 | 0.33 | 0.56 | 1.67 | 0.17 |
| IR22 | 15 | 11 | 1 | 3 | 44.44 | 0.44 | 0.67 | 0.33 | 0.33 | 1.00 | 0.29 |
| IR22 | 15 | 11 | 2 | 3 | 33.33 | 0.33 | 0.00 | 0.33 | 0.00 | 0.00 | 0.12 |
| IR22 | 15 | 11 | 3 | 3 | 0.00 | 0.00 | 0.00 | 0.33 | 0.33 | 1.00 |  |
| IR22 | 15 | 11 | 4 | 3 | 100.00 | 1.00 | 2.00 | 0.33 | 0.67 | 2.00 | 0.74 |
| IR22 | 15 | 12 | 1 | 3 | 22.22 | 0.22 | 0.78 | 0.33 | 1.17 | 3.50 | 0.20 |
| IR22 | 15 | 12 | 2 | 3 | 33.33 | 0.33 | 0.00 | 0.33 | 0.00 | 0.00 | 0.20 |
| IR22 | 15 | 12 | 3 | 3 | 0.00 | 0.00 | 0.00 | 0.33 | 1.17 | 3.50 |  |
| IR22 | 15 | 12 | 4 | 3 | 33.33 | 0.33 | 2.33 | 0.33 | 2.33 | 7.00 | 0.40 |
| IR22 | 15 | 13 | 1 | 3 | 22.22 | 0.22 | 0.00 | 0.33 | 0.00 | 0.00 | 0.21 |
| IR22 | 15 | 13 | 2 | 3 | 33.33 | 0.33 | 0.00 | 0.33 | 0.00 | 0.00 | 0.26 |
| IR22 | 15 | 13 | 3 | 3 | 0.00 | 0.00 | 0.00 | 0.33 | 0.00 | 0.00 |  |
| IR22 | 15 | 13 | 4 | 3 | 33.33 | 0.33 | 0.00 | 0.33 | 0.00 | 0.00 | 0.34 |
| IR22 | 15 | 14 | 1 | 3 | 22.22 | 0.22 | 1.11 | 0.33 | 1.67 | 5.00 | 0.26 |
| IR22 | 15 | 14 | 2 | 3 | 33.33 | 0.33 | 0.00 | 0.33 | 0.00 | 0.00 | 0.34 |
| IR22 | 15 | 14 | 3 | 3 | 0.00 | 0.00 | 0.00 | 0.33 | 1.67 | 5.00 |  |
| IR22 | 15 | 14 | 4 | 3 | 33.33 | 0.33 | 3.33 | 0.33 | 3.33 | 10.00 | 0.44 |
| IR22 | 15 | 15 | 1 | 3 | 22.22 | 0.22 | 0.00 | 0.33 | 0.00 | 0.00 | 0.22 |
| IR22 | 15 | 15 | 2 | 3 | 33.33 | 0.33 | 0.00 | 0.33 | 0.00 | 0.00 | 0.37 |
| IR22 | 15 | 15 | 3 | 3 | 0.00 | 0.00 | 0.00 | 0.33 | 0.00 | 0.00 |  |
| IR22 | 15 | 15 | 4 | 3 | 33.33 | 0.33 | 0.00 | 0.33 | 0.00 | 0.00 | 0.37 |
| IR22 | 15 | 16 | 1 | 3 | 11.11 | 0.11 | 0.00 | 0.33 | 0.00 | 0.00 | 0.91 |
| IR22 | 15 | 16 | 2 | 3 | 0.00 | 0.00 | 0.00 | 0.33 | 0.00 | 0.00 |  |
| IR22 | 15 | 16 | 3 | 3 | 0.00 | 0.00 | 0.00 | 0.33 | 0.00 | 0.00 |  |
| IR22 | 15 | 16 | 4 | 3 | 33.33 | 0.33 | 0.00 | 0.33 | 0.00 | 0.00 | 0.27 |
| IR22 | 15 | 17 | 1 | 3 | 11.11 | 0.11 | 0.00 | 0.33 | 0.00 | 0.00 | 0.18 |
| IR22 | 15 | 17 | 2 | 3 | 0.00 | 0.00 | 0.00 | 0.33 | 0.00 | 0.00 |  |
| IR22 | 15 | 17 | 3 | 3 | 0.00 | 0.00 | 0.00 | 0.33 | 0.00 | 0.00 |  |
| IR22 | 15 | 17 | 4 | 3 | 33.33 | 0.33 | 0.00 | 0.33 | 0.00 | 0.00 | 0.53 |
| IR22 | 15 | 18 | 1 | 3 | 11.11 | 0.11 | 0.00 | 0.33 | 0.00 | 0.00 | 0.14 |
| IR22 | 15 | 18 | 2 | 3 | 0.00 | 0.00 | 0.00 | 0.33 | 0.00 | 0.00 |  |
| IR22 | 15 | 18 | 3 | 3 | 0.00 | 0.00 | 0.00 | 0.33 | 0.00 | 0.00 |  |
| IR22 | 15 | 18 | 4 | 3 | 33.33 | 0.33 | 0.00 | 0.33 | 0.00 | 0.00 | 0.43 |
| IR22 | 15 | 19 | 1 | 3 | 0.00 | 0.00 | 0.00 |  |  |  |  |
| IR22 | 15 | 19 | 2 | 3 | 0.00 | 0.00 | 0.00 |  |  |  |  |
| IR22 | 15 | 19 | 3 | 3 | 0.00 | 0.00 | 0.00 |  |  |  |  |
| IR22 | 15 | 19 | 4 | 3 | 0.00 | 0.00 | 0.00 |  |  |  |  |
| IR22 | 15 | 20 | 1 | 3 | 0.00 | 0.00 | 0.00 |  |  |  |  |
| IR22 | 15 | 20 | 2 | 3 | 0.00 | 0.00 | 0.00 |  |  |  |  |
| IR22 | 15 | 20 | 3 | 3 | 0.00 | 0.00 | 0.00 |  |  |  |  |
| IR22 | 15 | 20 | 4 | 3 | 0.00 | 0.00 | 0.00 |  |  |  |  |
| IR22 | 20 | 1 | 1 | 5 | 100.00 | 0.80 | 3.40 | 0.27 | 1.13 | 4.25 | 0.15 |
| IR22 | 20 | 1 | 2 | 5 | 100.00 | 1.40 | 9.20 | 0.47 | 3.07 | 6.57 | 0.14 |
| IR22 | 20 | 1 | 3 | 3 | 100.00 | 10.33 | 51.00 | 3.44 | 17.00 | 4.94 | 0.41 |
| IR22 | 20 | 1 | 4 | 3 | 100.00 | 6.67 | 26.00 | 2.22 | 8.67 | 3.90 | 0.55 |
| IR22 | 20 | 2 | 1 | 5 | 100.00 | 1.00 | 1.60 | 0.33 | 0.53 | 1.60 | 0.16 |
| IR22 | 20 | 2 | 2 | 5 | 100.00 | 4.20 | 15.80 | 1.40 | 5.27 | 3.76 | 0.35 |
| IR22 | 20 | 2 | 3 | 3 | 100.00 | 5.33 | 24.00 | 1.78 | 8.00 | 4.50 | 0.38 |
| IR22 | 20 | 2 | 4 | 3 | 100.00 | 1.00 | 0.00 | 0.33 | 0.00 | 0.00 | 0.56 |
| IR22 | 20 | 3 | 1 | 5 | 100.00 | 3.00 | 10.20 | 1.00 | 3.40 | 3.40 | 0.15 |
| IR22 | 20 | 3 | 2 | 5 | 100.00 | 4.60 | 25.20 | 1.53 | 8.40 | 5.48 | 0.52 |
| IR22 | 20 | 3 | 3 | 3 | 100.00 | 4.33 | 16.67 | 1.44 | 5.56 | 3.85 | 0.38 |
| IR22 | 20 | 3 | 4 | 3 | 66.67 | 1.00 | 6.00 | 0.50 | 3.00 | 6.00 | 0.33 |
| IR22 | 20 | 4 | 1 | 5 | 100.00 | 4.00 | 12.00 | 1.33 | 4.00 | 3.00 | 0.17 |
| IR22 | 20 | 4 | 2 | 5 | 100.00 | 0.60 | 2.20 | 0.20 | 0.73 | 3.67 | 0.54 |
| IR22 | 20 | 4 | 3 | 3 | 100.00 | 3.00 | 9.67 | 1.00 | 3.22 | 3.22 | 0.54 |
| IR22 | 20 | 4 | 4 | 3 | 33.33 | 0.33 | 0.00 | 0.33 | 0.00 | 0.00 | 0.18 |
| IR22 | 20 | 5 | 1 | 5 | 100.00 | 5.60 | 16.40 | 1.87 | 5.47 | 2.93 | 0.17 |
| IR22 | 20 | 5 | 2 | 5 | 80.00 | 2.60 | 11.20 | 1.08 | 4.67 | 4.31 | 0.35 |
| IR22 | 20 | 5 | 3 | 3 | 100.00 | 3.33 | 10.67 | 1.11 | 3.56 | 3.20 | 0.46 |
| IR22 | 20 | 5 | 4 | 3 | 33.33 | 0.33 | 2.00 | 0.33 | 2.00 | 6.00 | 0.18 |
| IR22 | 20 | 6 | 1 | 5 | 100.00 | 3.80 | 8.60 | 1.27 | 2.87 | 2.26 | 0.18 |
| IR22 | 20 | 6 | 2 | 5 | 60.00 | 2.20 | 8.40 | 1.22 | 4.67 | 3.82 | 0.27 |
| IR22 | 20 | 6 | 3 | 3 | 100.00 | 2.33 | 6.00 | 0.78 | 2.00 | 2.57 | 0.56 |
| IR22 | 20 | 6 | 4 | 3 | 33.33 | 0.33 | 0.00 | 0.33 | 0.00 | 0.00 | 0.35 |
| IR22 | 20 | 7 | 1 | 5 | 80.00 | 3.00 | 7.80 | 1.25 | 3.25 | 2.60 | 0.18 |
| IR22 | 20 | 7 | 2 | 5 | 60.00 | 2.80 | 9.80 | 1.56 | 5.44 | 3.50 | 0.27 |
| IR22 | 20 | 7 | 3 | 3 | 66.67 | 2.67 | 6.67 | 1.33 | 3.33 | 2.50 | 0.40 |
| IR22 | 20 | 7 | 4 | 3 | 0.00 | 0.00 | 0.00 | 1.38 | 4.01 | 2.87 |  |
| IR22 | 20 | 8 | 1 | 5 | 42.22 | 1.29 | 4.67 | 1.00 | 3.61 | 3.54 | 0.24 |
| IR22 | 20 | 8 | 2 | 5 | 60.00 | 1.20 | 4.00 | 0.67 | 2.22 | 3.33 | 0.41 |
| IR22 | 20 | 8 | 3 | 3 | 66.67 | 2.67 | 10.00 | 1.33 | 5.00 | 3.75 | 0.32 |
| IR22 | 20 | 8 | 4 | 3 | 0.00 | 0.00 | 0.00 | 1.00 | 3.61 | 3.54 |  |
| IR22 | 20 | 9 | 1 | 5 | 42.22 | 1.27 | 5.04 | 1.00 | 4.00 | 4.00 | 0.26 |
| IR22 | 20 | 9 | 2 | 5 | 60.00 | 1.80 | 7.80 | 1.00 | 4.33 | 4.33 | 0.34 |
| IR22 | 20 | 9 | 3 | 3 | 66.67 | 2.00 | 7.33 | 1.00 | 3.67 | 3.67 | 0.43 |
| IR22 | 20 | 9 | 4 | 3 | 0.00 | 0.00 | 0.00 | 1.00 | 4.00 | 4.00 |  |
| IR22 | 20 | 10 | 1 | 5 | 42.22 | 0.56 | 1.33 | 0.44 | 1.11 | 2.00 | 0.26 |
| IR22 | 20 | 10 | 2 | 5 | 60.00 | 1.00 | 4.00 | 0.56 | 2.22 | 4.00 | 0.35 |
| IR22 | 20 | 10 | 3 | 3 | 66.67 | 0.67 | 0.00 | 0.33 | 0.00 | 0.00 | 0.42 |
| IR22 | 20 | 10 | 4 | 3 | 0.00 | 0.00 | 0.00 | 0.44 | 1.11 | 2.00 |  |
| IR22 | 20 | 11 | 1 | 5 | 42.22 | 1.09 | 1.56 | 0.83 | 1.19 | 1.52 | 0.24 |
| IR22 | 20 | 11 | 2 | 5 | 60.00 | 0.60 | 1.00 | 0.33 | 0.56 | 1.67 | 0.39 |
| IR22 | 20 | 11 | 3 | 3 | 66.67 | 2.67 | 3.67 | 1.33 | 1.83 | 1.38 | 0.34 |
| IR22 | 20 | 11 | 4 | 3 | 0.00 | 0.00 | 0.00 | 0.83 | 1.19 | 1.52 |  |
| IR22 | 20 | 12 | 1 | 5 | 42.22 | 0.82 | 3.09 | 0.67 | 2.50 | 3.83 | 0.24 |
| IR22 | 20 | 12 | 2 | 5 | 60.00 | 1.80 | 6.60 | 1.00 | 3.67 | 3.67 | 0.35 |
| IR22 | 20 | 12 | 3 | 3 | 66.67 | 0.67 | 2.67 | 0.33 | 1.33 | 4.00 | 0.39 |
| IR22 | 20 | 12 | 4 | 3 | 0.00 | 0.00 | 0.00 | 0.67 | 2.50 | 3.83 |  |
| IR22 | 20 | 13 | 1 | 5 | 31.11 | 0.31 | 1.84 | 0.33 | 2.50 | 7.50 | 0.24 |
| IR22 | 20 | 13 | 2 | 5 | 60.00 | 0.60 | 1.20 | 0.33 | 0.67 | 2.00 | 0.36 |
| IR22 | 20 | 13 | 3 | 3 | 33.33 | 0.33 | 4.33 | 0.33 | 4.33 | 13.00 | 0.37 |
| IR22 | 20 | 13 | 4 | 3 | 0.00 | 0.00 | 0.00 | 0.33 | 2.50 | 7.50 |  |
| IR22 | 20 | 14 | 1 | 5 | 31.11 | 0.31 | 0.67 | 0.33 | 0.56 | 1.67 | 0.26 |
| IR22 | 20 | 14 | 2 | 5 | 60.00 | 0.60 | 2.00 | 0.33 | 1.11 | 3.33 | 0.45 |
| IR22 | 20 | 14 | 3 | 3 | 33.33 | 0.33 | 0.00 | 0.33 | 0.00 | 0.00 | 0.32 |
| IR22 | 20 | 14 | 4 | 3 | 0.00 | 0.00 | 0.00 | 0.33 | 0.56 | 1.67 |  |
| IR22 | 20 | 15 | 1 | 5 | 24.44 | 0.47 | 2.62 | 0.67 | 3.83 | 4.83 | 0.19 |
| IR22 | 20 | 15 | 2 | 5 | 40.00 | 0.40 | 1.20 | 0.33 | 1.00 | 3.00 | 0.25 |
| IR22 | 20 | 15 | 3 | 3 | 33.33 | 1.00 | 6.67 | 1.00 | 6.67 | 6.67 | 0.31 |
| IR22 | 20 | 15 | 4 | 3 | 0.00 | 0.00 | 0.00 | 0.67 | 3.83 | 4.83 |  |
| IR22 | 20 | 16 | 1 | 5 | 17.78 | 0.22 | 1.00 | 0.33 | 1.50 | 4.50 | 0.14 |
| IR22 | 20 | 16 | 2 | 5 | 20.00 | 0.00 | 0.00 | 0.00 | 0.00 | 4.50 | 0.15 |
| IR22 | 20 | 16 | 3 | 3 | 33.33 | 0.67 | 3.00 | 0.67 | 3.00 | 4.50 | 0.28 |
| IR22 | 20 | 16 | 4 | 3 | 0.00 | 0.00 | 0.00 | 0.33 | 1.50 | 4.50 |  |
| IR22 | 20 | 17 | 1 | 5 | 17.78 | 0.24 | 0.40 | 0.50 | 1.00 | 1.50 | 0.16 |
| IR22 | 20 | 17 | 2 | 5 | 20.00 | 0.40 | 1.20 | 0.67 | 2.00 | 3.00 | 0.16 |
| IR22 | 20 | 17 | 3 | 3 | 33.33 | 0.33 | 0.00 | 0.33 | 0.00 | 0.00 | 0.33 |
| IR22 | 20 | 17 | 4 | 3 | 0.00 | 0.00 | 0.00 | 0.50 | 1.00 | 1.50 |  |
| IR22 | 20 | 18 | 1 | 5 | 17.78 | 0.11 | 0.00 |  |  |  | 0.18 |
| IR22 | 20 | 18 | 2 | 5 | 20.00 | 0.00 | 0.00 |  |  |  | 0.14 |
| IR22 | 20 | 18 | 3 | 3 | 33.33 | 0.33 | 0.00 |  |  |  | 0.41 |
| IR22 | 20 | 18 | 4 | 3 | 0.00 | 0.00 | 0.00 |  |  |  |  |
| IR22 | 20 | 19 | 1 | 5 | 17.78 | 0.11 | 0.00 |  |  |  | 0.28 |
| IR22 | 20 | 19 | 2 | 5 | 20.00 | 0.00 | 0.00 |  |  |  | 0.26 |
| IR22 | 20 | 19 | 3 | 3 | 33.33 | 0.33 | 0.00 |  |  |  | 0.57 |
| IR22 | 20 | 19 | 4 | 3 | 0.00 | 0.00 | 0.00 |  |  |  |  |
| IR22 | 20 | 20 | 1 | 5 | 6.67 | 0.00 | 0.00 |  |  |  | 0.65 |
| IR22 | 20 | 20 | 2 | 5 | 20.00 | 0.00 | 0.00 |  |  |  | 0.19 |
| IR22 | 20 | 20 | 3 | 3 | 0.00 | 0.00 | 0.00 |  |  |  |  |
| IR22 | 20 | 20 | 4 | 3 | 0.00 | 0.00 | 0.00 |  |  |  |  |
| IR22 | 25 | 1 | 1 | 5 | 100.00 | 2.20 | 7.00 | 0.73 | 2.33 | 3.18 | 0.15 |
| IR22 | 25 | 1 | 2 | 5 | 100.00 | 3.60 | 26.20 | 1.20 | 8.73 | 7.28 | 0.83 |
| IR22 | 25 | 1 | 3 | 3 | 100.00 | 7.00 | 19.00 | 2.33 | 6.33 | 2.71 | 0.23 |
| IR22 | 25 | 1 | 4 | 3 | 100.00 | 12.00 | 42.67 | 4.00 | 14.22 | 3.56 | 0.18 |
| IR22 | 25 | 2 | 1 | 5 | 100.00 | 5.60 | 17.00 | 1.87 | 5.67 | 3.04 | 0.16 |
| IR22 | 25 | 2 | 2 | 5 | 80.00 | 8.20 | 37.80 | 3.42 | 15.75 | 4.61 | 0.32 |
| IR22 | 25 | 2 | 3 | 3 | 100.00 | 1.67 | 4.67 | 0.56 | 1.56 | 2.80 | 0.29 |
| IR22 | 25 | 2 | 4 | 3 | 100.00 | 2.33 | 13.00 | 0.78 | 4.33 | 5.57 | 0.47 |
| IR22 | 25 | 3 | 1 | 5 | 100.00 | 6.00 | 15.00 | 2.00 | 5.00 | 2.50 | 0.12 |
| IR22 | 25 | 3 | 2 | 5 | 80.00 | 3.20 | 13.40 | 1.33 | 5.58 | 4.19 | 0.44 |
| IR22 | 25 | 3 | 3 | 3 | 100.00 | 2.00 | 4.67 | 0.67 | 1.56 | 2.33 | 0.21 |
| IR22 | 25 | 3 | 4 | 3 | 100.00 | 4.00 | 23.33 | 1.33 | 7.78 | 5.83 | 0.37 |
| IR22 | 25 | 4 | 1 | 5 | 100.00 | 5.60 | 13.40 | 1.87 | 4.47 | 2.39 | 0.14 |
| IR22 | 25 | 4 | 2 | 5 | 80.00 | 4.60 | 27.40 | 1.92 | 11.42 | 5.96 | 0.45 |
| IR22 | 25 | 4 | 3 | 3 | 100.00 | 1.00 | 0.00 | 0.33 | 0.00 | 0.00 | 0.29 |
| IR22 | 25 | 4 | 4 | 3 | 100.00 | 6.67 | 34.33 | 2.22 | 11.44 | 5.15 | 0.28 |
| IR22 | 25 | 5 | 1 | 5 | 100.00 | 5.60 | 17.80 | 1.87 | 5.93 | 3.18 | 0.20 |
| IR22 | 25 | 5 | 2 | 5 | 80.00 | 4.60 | 25.80 | 1.92 | 10.75 | 5.61 | 0.44 |
| IR22 | 25 | 5 | 3 | 3 | 66.67 | 0.67 | 0.00 | 0.33 | 0.00 | 0.00 | 0.27 |
| IR22 | 25 | 5 | 4 | 3 | 66.67 | 0.67 | 0.00 | 0.33 | 0.00 | 0.00 | 0.32 |
| IR22 | 25 | 6 | 1 | 5 | 100.00 | 7.40 | 16.80 | 2.47 | 5.60 | 2.27 | 0.19 |
| IR22 | 25 | 6 | 2 | 5 | 80.00 | 4.00 | 19.00 | 1.67 | 7.92 | 4.75 | 0.37 |
| IR22 | 25 | 6 | 3 | 3 | 0.00 | 0.00 | 0.00 | 1.49 | 4.51 | 2.34 |  |
| IR22 | 25 | 6 | 4 | 3 | 33.33 | 0.33 | 0.00 | 0.33 | 0.00 | 0.00 | 0.19 |
| IR22 | 25 | 7 | 1 | 5 | 100.00 | 5.00 | 14.80 | 1.67 | 4.93 | 2.96 | 0.28 |
| IR22 | 25 | 7 | 2 | 5 | 80.00 | 3.60 | 16.40 | 1.50 | 6.83 | 4.56 | 0.47 |
| IR22 | 25 | 7 | 3 | 3 | 0.00 | 0.00 | 0.00 | 1.58 | 5.88 | 3.76 |  |
| IR22 | 25 | 7 | 4 | 3 | 0.00 | 0.00 | 0.00 | 1.58 | 5.88 | 3.76 |  |
| IR22 | 25 | 8 | 1 | 5 | 20.00 | 0.73 | 4.07 | 1.22 | 6.78 | 5.55 | 0.16 |
| IR22 | 25 | 8 | 2 | 5 | 60.00 | 2.20 | 12.20 | 1.22 | 6.78 | 5.55 | 0.47 |
| IR22 | 25 | 8 | 3 | 3 | 0.00 | 0.00 | 0.00 | 1.22 | 6.78 | 5.55 |  |
| IR22 | 25 | 8 | 4 | 3 | 0.00 | 0.00 | 0.00 | 1.22 | 6.78 | 5.55 |  |
| IR22 | 25 | 9 | 1 | 5 | 13.33 | 0.87 | 4.27 | 2.17 | 10.67 | 4.92 | 0.69 |
| IR22 | 25 | 9 | 2 | 5 | 40.00 | 2.60 | 12.80 | 2.17 | 10.67 | 4.92 | 0.26 |
| IR22 | 25 | 9 | 3 | 3 | 0.00 | 0.00 | 0.00 | 2.17 | 10.67 | 4.92 |  |
| IR22 | 25 | 9 | 4 | 3 | 0.00 | 0.00 | 0.00 | 2.17 | 10.67 | 4.92 |  |
| IR22 | 25 | 10 | 1 | 5 | 13.33 | 0.73 | 4.87 | 1.83 | 12.17 | 6.64 | 0.19 |
| IR22 | 25 | 10 | 2 | 5 | 40.00 | 2.20 | 14.60 | 1.83 | 12.17 | 6.64 | 0.33 |
| IR22 | 25 | 10 | 3 | 3 | 0.00 | 0.00 | 0.00 | 1.83 | 12.17 | 6.64 |  |
| IR22 | 25 | 10 | 4 | 3 | 0.00 | 0.00 | 0.00 | 1.83 | 12.17 | 6.64 |  |
| IR22 | 25 | 11 | 1 | 5 | 13.33 | 0.67 | 2.60 | 1.67 | 6.50 | 3.90 | 0.78 |
| IR22 | 25 | 11 | 2 | 5 | 40.00 | 2.00 | 7.80 | 1.67 | 6.50 | 3.90 | 0.23 |
| IR22 | 25 | 11 | 3 | 3 | 0.00 | 0.00 | 0.00 | 1.67 | 6.50 | 3.90 |  |
| IR22 | 25 | 11 | 4 | 3 | 0.00 | 0.00 | 0.00 | 1.67 | 6.50 | 3.90 |  |
| IR22 | 25 | 12 | 1 | 5 | 13.33 | 0.13 | 0.47 | 0.33 | 1.17 | 3.50 | 0.87 |
| IR22 | 25 | 12 | 2 | 5 | 40.00 | 0.40 | 1.40 | 0.33 | 1.17 | 3.50 | 0.26 |
| IR22 | 25 | 12 | 3 | 3 | 0.00 | 0.00 | 0.00 | 0.33 | 1.17 | 3.50 |  |
| IR22 | 25 | 12 | 4 | 3 | 0.00 | 0.00 | 0.00 | 0.33 | 1.17 | 3.50 |  |
| IR22 | 25 | 13 | 1 | 5 | 13.33 | 0.20 | 0.67 | 0.50 | 1.67 | 3.33 | 0.80 |
| IR22 | 25 | 13 | 2 | 5 | 40.00 | 0.60 | 2.00 | 0.50 | 1.67 | 3.33 | 0.24 |
| IR22 | 25 | 13 | 3 | 3 | 0.00 | 0.00 | 0.00 | 0.50 | 1.67 | 3.33 |  |
| IR22 | 25 | 13 | 4 | 3 | 0.00 | 0.00 | 0.00 | 0.50 | 1.67 | 3.33 |  |
| IR22 | 25 | 14 | 1 | 5 | 13.33 | 0.40 | 2.00 | 1.00 | 5.00 | 5.00 | 0.91 |
| IR22 | 25 | 14 | 2 | 5 | 40.00 | 1.20 | 6.00 | 1.00 | 5.00 | 5.00 | 0.27 |
| IR22 | 25 | 14 | 3 | 3 | 0.00 | 0.00 | 0.00 | 1.00 | 5.00 | 5.00 |  |
| IR22 | 25 | 14 | 4 | 3 | 0.00 | 0.00 | 0.00 | 1.00 | 5.00 | 5.00 |  |
| IR22 | 25 | 15 | 1 | 5 | 13.33 | 0.27 | 1.40 | 0.67 | 3.50 | 5.25 | 0.98 |
| IR22 | 25 | 15 | 2 | 5 | 40.00 | 0.80 | 4.20 | 0.67 | 3.50 | 5.25 | 0.29 |
| IR22 | 25 | 15 | 3 | 3 | 0.00 | 0.00 | 0.00 | 0.67 | 3.50 | 5.25 |  |
| IR22 | 25 | 15 | 4 | 3 | 0.00 | 0.00 | 0.00 | 0.67 | 3.50 | 5.25 |  |
| IR22 | 25 | 16 | 1 | 5 | 6.67 | 0.27 | 0.87 | 1.33 | 4.33 | 3.25 | 0.33 |
| IR22 | 25 | 16 | 2 | 5 | 20.00 | 0.80 | 2.60 | 1.33 | 4.33 | 3.25 | 0.98 |
| IR22 | 25 | 16 | 3 | 3 | 0.00 | 0.00 | 0.00 | 1.33 | 4.33 | 3.25 |  |
| IR22 | 25 | 16 | 4 | 3 | 0.00 | 0.00 | 0.00 | 1.33 | 4.33 | 3.25 |  |
| IR22 | 25 | 17 | 1 | 5 | 0.00 | 0.00 | 0.00 | 0.67 | 0.67 | 1.00 |  |
| IR22 | 25 | 17 | 2 | 5 | 20.00 | 0.40 | 0.40 | 0.67 | 0.67 | 1.00 | 0.13 |
| IR22 | 25 | 17 | 3 | 5 | 0.00 | 0.00 | 0.00 | 0.67 | 0.67 | 1.00 |  |
| IR22 | 25 | 17 | 4 | 5 | 0.00 | 0.00 | 0.00 | 0.67 | 0.67 | 1.00 |  |
| IR22 | 25 | 18 | 1 | 5 | 0.00 | 0.00 | 0.00 |  |  |  |  |
| IR22 | 25 | 18 | 2 | 5 | 20.00 | 0.00 | 0.00 |  |  |  | 0.16 |
| IR22 | 25 | 18 | 3 | 5 | 0.00 | 0.00 | 0.00 |  |  |  |  |
| IR22 | 25 | 18 | 4 | 5 | 0.00 | 0.00 | 0.00 |  |  |  |  |
| IR22 | 25 | 19 | 1 | 5 | 0.00 | 0.00 | 0.00 |  |  |  | 0.23 |
| IR22 | 25 | 19 | 2 | 5 | 20.00 | 0.00 | 0.00 |  |  |  | 0.23 |
| IR22 | 25 | 19 | 3 | 5 | 0.00 | 0.00 | 0.00 |  |  |  | 0.23 |
| IR22 | 25 | 19 | 4 | 5 | 0.00 | 0.00 | 0.00 |  |  |  | 0.23 |
| IR22 | 25 | 20 | 1 | 5 | 0.00 | 0.00 | 0.00 |  |  |  | 0.16 |
| IR22 | 25 | 20 | 2 | 5 | 20.00 | 0.00 | 0.00 |  |  |  | 0.16 |
| IR22 | 25 | 20 | 3 | 5 | 0.00 | 0.00 | 0.00 |  |  |  | 0.16 |
| IR22 | 25 | 20 | 4 | 5 | 0.00 | 0.00 | 0.00 |  |  |  | 0.16 |
| IR22 | 30 | 1 | 1 | 5 | 100.00 | 0.80 | 1.20 | 0.27 | 0.40 | 1.50 | 0.12 |
| IR22 | 30 | 1 | 2 | 5 | 100.00 | 5.40 | 28.80 | 1.80 | 9.60 | 5.33 | 0.79 |
| IR22 | 30 | 1 | 3 | 3 | 100.00 | 9.00 | 34.00 | 3.00 | 11.33 | 3.78 | 0.26 |
| IR22 | 30 | 1 | 4 | 3 | 100.00 | 19.33 | 71.33 | 6.44 | 23.78 | 3.69 | 0.33 |
| IR22 | 30 | 2 | 1 | 5 | 100.00 | 6.20 | 22.20 | 2.07 | 7.40 | 3.58 | 0.15 |
| IR22 | 30 | 2 | 2 | 5 | 80.00 | 12.80 | 47.80 | 5.33 | 19.92 | 3.73 | 0.29 |
| IR22 | 30 | 2 | 3 | 3 | 100.00 | 2.67 | 6.67 | 0.89 | 2.22 | 2.50 | 0.28 |
| IR22 | 30 | 2 | 4 | 3 | 100.00 | 11.00 | 48.33 | 3.67 | 16.11 | 4.39 | 0.37 |
| IR22 | 30 | 3 | 1 | 5 | 100.00 | 7.60 | 24.00 | 2.53 | 8.00 | 3.16 | 0.15 |
| IR22 | 30 | 3 | 2 | 5 | 80.00 | 6.20 | 25.80 | 2.58 | 10.75 | 4.16 | 0.56 |
| IR22 | 30 | 3 | 3 | 3 | 66.67 | 1.00 | 3.00 | 0.50 | 1.50 | 3.00 | 0.19 |
| IR22 | 30 | 3 | 4 | 3 | 100.00 | 11.00 | 44.67 | 3.67 | 14.89 | 4.06 | 0.36 |
| IR22 | 30 | 4 | 1 | 5 | 100.00 | 6.00 | 19.60 | 2.00 | 6.53 | 3.27 | 0.15 |
| IR22 | 30 | 4 | 2 | 5 | 60.00 | 2.60 | 12.00 | 1.44 | 6.67 | 4.62 | 0.32 |
| IR22 | 30 | 4 | 3 | 3 | 33.33 | 0.67 | 1.00 | 0.67 | 1.00 | 1.50 | 0.12 |
| IR22 | 30 | 4 | 4 | 3 | 66.67 | 4.00 | 12.33 | 2.00 | 6.17 | 3.08 | 0.24 |
| IR22 | 30 | 5 | 1 | 5 | 100.00 | 5.40 | 19.60 | 1.80 | 6.53 | 3.63 | 0.23 |
| IR22 | 30 | 5 | 2 | 5 | 60.00 | 4.60 | 17.00 | 2.56 | 9.44 | 3.70 | 0.24 |
| IR22 | 30 | 5 | 3 | 3 | 33.33 | 0.67 | 3.33 | 0.67 | 3.33 | 5.00 | 0.16 |
| IR22 | 30 | 5 | 4 | 3 | 33.33 | 2.67 | 16.33 | 2.67 | 16.33 | 6.13 | 0.18 |
| IR22 | 30 | 6 | 1 | 5 | 80.00 | 4.40 | 13.20 | 1.83 | 5.50 | 3.00 | 0.20 |
| IR22 | 30 | 6 | 2 | 5 | 60.00 | 3.40 | 16.00 | 1.89 | 8.89 | 4.71 | 0.29 |
| IR22 | 30 | 6 | 3 | 3 | 33.33 | 0.33 | 1.67 | 0.33 | 1.67 | 5.00 | 0.14 |
| IR22 | 30 | 6 | 4 | 3 | 33.33 | 0.33 | 0.00 | 0.33 | 0.00 | 0.00 | 0.17 |
| IR22 | 30 | 7 | 1 | 5 | 80.00 | 3.20 | 11.00 | 1.33 | 4.58 | 3.44 | 0.19 |
| IR22 | 30 | 7 | 2 | 5 | 60.00 | 1.60 | 5.80 | 0.89 | 3.22 | 3.63 | 0.27 |
| IR22 | 30 | 7 | 3 | 3 | 33.33 | 0.33 | 0.00 | 0.33 | 0.00 | 0.00 | 0.14 |
| IR22 | 30 | 7 | 4 | 3 | 0.00 | 0.00 | 0.00 | 0.85 | 2.60 | 2.35 |  |
| IR22 | 30 | 8 | 1 | 5 | 13.33 | 0.40 | 1.80 | 1.00 | 4.50 | 4.50 | 0.14 |
| IR22 | 30 | 8 | 2 | 5 | 40.00 | 1.20 | 5.40 | 1.00 | 4.50 | 4.50 | 0.31 |
| IR22 | 30 | 8 | 3 | 3 | 0.00 | 0.00 | 0.00 | 1.00 | 4.50 | 4.50 |  |
| IR22 | 30 | 8 | 4 | 3 | 0.00 | 0.00 | 0.00 | 1.00 | 4.50 | 4.50 |  |
| IR22 | 30 | 9 | 1 | 5 | 13.33 | 0.80 | 3.60 | 2.00 | 9.00 | 4.50 | 0.71 |
| IR22 | 30 | 9 | 2 | 5 | 40.00 | 2.40 | 10.80 | 2.00 | 9.00 | 4.50 | 0.21 |
| IR22 | 30 | 9 | 3 | 3 | 0.00 | 0.00 | 0.00 | 2.00 | 9.00 | 4.50 |  |
| IR22 | 30 | 9 | 4 | 3 | 0.00 | 0.00 | 0.00 | 2.00 | 9.00 | 4.50 |  |
| IR22 | 30 | 10 | 1 | 5 | 13.33 | 0.73 | 4.07 | 1.83 | 10.17 | 5.55 | 0.73 |
| IR22 | 30 | 10 | 2 | 5 | 40.00 | 2.20 | 12.20 | 1.83 | 10.17 | 5.55 | 0.22 |
| IR22 | 30 | 10 | 3 | 3 | 0.00 | 0.00 | 0.00 | 1.83 | 10.17 | 5.55 |  |
| IR22 | 30 | 10 | 4 | 3 | 0.00 | 0.00 | 0.00 | 1.83 | 10.17 | 5.55 |  |
| IR22 | 30 | 11 | 1 | 5 | 13.33 | 1.07 | 3.80 | 2.67 | 9.50 | 3.56 | 0.75 |
| IR22 | 30 | 11 | 2 | 5 | 40.00 | 3.20 | 11.40 | 2.67 | 9.50 | 3.56 | 0.23 |
| IR22 | 30 | 11 | 3 | 3 | 0.00 | 0.00 | 0.00 | 2.67 | 9.50 | 3.56 |  |
| IR22 | 30 | 11 | 4 | 3 | 0.00 | 0.00 | 0.00 | 2.67 | 9.50 | 3.56 |  |
| IR22 | 30 | 12 | 1 | 5 | 13.33 | 0.20 | 2.07 | 0.50 | 5.17 | 10.33 | 0.85 |
| IR22 | 30 | 12 | 2 | 5 | 40.00 | 0.60 | 6.20 | 0.50 | 5.17 | 10.33 | 0.25 |
| IR22 | 30 | 12 | 3 | 3 | 0.00 | 0.00 | 0.00 | 0.50 | 5.17 | 10.33 |  |
| IR22 | 30 | 12 | 4 | 3 | 0.00 | 0.00 | 0.00 | 0.50 | 5.17 | 10.33 |  |
| IR22 | 30 | 13 | 1 | 5 | 13.33 | 0.73 | 3.27 | 1.83 | 8.17 | 4.45 | 0.72 |
| IR22 | 30 | 13 | 2 | 5 | 40.00 | 2.20 | 9.80 | 1.83 | 8.17 | 4.45 | 0.22 |
| IR22 | 30 | 13 | 3 | 3 | 0.00 | 0.00 | 0.00 | 1.83 | 8.17 | 4.45 |  |
| IR22 | 30 | 13 | 4 | 3 | 0.00 | 0.00 | 0.00 | 1.83 | 8.17 | 4.45 |  |
| IR22 | 30 | 14 | 1 | 5 | 0.00 | 0.00 | 0.00 | 0.00 | 0.00 |  |  |
| IR22 | 30 | 14 | 2 | 5 | 40.00 | 0.00 | 0.00 | 0.00 | 0.00 |  | 0.26 |
| IR22 | 30 | 14 | 3 | 3 | 0.00 | 0.00 | 0.00 | 0.00 | 0.00 |  |  |
| IR22 | 30 | 14 | 4 | 3 | 0.00 | 0.00 | 0.00 | 0.00 | 0.00 |  |  |
| IR22 | 30 | 15 | 1 | 5 | 0.00 | 0.00 | 0.00 | 1.33 | 5.67 | 4.25 |  |
| IR22 | 30 | 15 | 2 | 5 | 20.00 | 0.80 | 3.40 | 1.33 | 5.67 | 4.25 | 0.16 |
| IR22 | 30 | 15 | 3 | 3 | 0.00 | 0.00 | 0.00 | 1.33 | 5.67 | 4.25 |  |
| IR22 | 30 | 15 | 4 | 3 | 0.00 | 0.00 | 0.00 | 1.33 | 5.67 | 4.25 |  |
| IR22 | 30 | 16 | 1 | 5 | 0.00 | 0.00 | 0.00 | 0.33 | 2.33 | 7.00 |  |
| IR22 | 30 | 16 | 2 | 5 | 20.00 | 0.20 | 1.40 | 0.33 | 2.33 | 7.00 | 0.15 |
| IR22 | 30 | 16 | 3 | 3 | 0.00 | 0.00 | 0.00 | 0.33 | 2.33 | 7.00 |  |
| IR22 | 30 | 16 | 4 | 3 | 0.00 | 0.00 | 0.00 | 0.33 | 2.33 | 7.00 |  |
| IR22 | 30 | 17 | 1 | 5 | 0.00 | 0.00 | 0.00 | 1.33 | 12.00 | 9.00 |  |
| IR22 | 30 | 17 | 2 | 5 | 20.00 | 0.80 | 7.20 | 1.33 | 12.00 | 9.00 | 0.16 |
| IR22 | 30 | 17 | 3 | 5 | 0.00 | 0.00 | 0.00 | 1.33 | 12.00 | 9.00 |  |
| IR22 | 30 | 17 | 4 | 5 | 0.00 | 0.00 | 0.00 | 1.33 | 12.00 | 9.00 |  |
| IR22 | 30 | 18 | 1 | 5 | 0.00 | 0.00 | 0.00 |  |  |  |  |
| IR22 | 30 | 18 | 2 | 5 | 20.00 | 0.00 | 0.00 |  |  |  | 0.15 |
| IR22 | 30 | 18 | 3 | 5 | 0.00 | 0.00 | 0.00 |  |  |  |  |
| IR22 | 30 | 18 | 4 | 5 | 0.00 | 0.00 | 0.00 |  |  |  |  |
| IR22 | 30 | 19 | 1 | 5 | 0.00 | 0.00 | 0.00 |  |  |  |  |
| IR22 | 30 | 19 | 2 | 5 | 0.00 | 0.00 | 0.00 |  |  |  |  |
| IR22 | 30 | 19 | 3 | 5 | 0.00 | 0.00 | 0.00 |  |  |  |  |
| IR22 | 30 | 19 | 4 | 5 | 0.00 | 0.00 | 0.00 |  |  |  |  |
| IR22 | 30 | 20 | 1 | 5 | 0.00 | 0.00 | 0.00 |  |  |  |  |
| IR22 | 30 | 20 | 2 | 5 | 0.00 | 0.00 | 0.00 |  |  |  |  |
| IR22 | 30 | 20 | 3 | 5 | 0.00 | 0.00 | 0.00 |  |  |  |  |
| IR22 | 30 | 20 | 4 | 5 | 0.00 | 0.00 | 0.00 |  |  |  |  |
| IR22 | 35 | 1 | 1 | 5 | 100.00 | 9.40 | 40.80 | 3.13 | 13.60 | 4.34 | 0.49 |
| IR22 | 35 | 1 | 2 | 3 | 100.00 | 9.00 | 30.00 | 3.00 | 10.00 | 3.33 | 0.19 |
| IR22 | 35 | 1 | 3 | 3 | 100.00 | 5.00 | 15.67 | 1.67 | 5.22 | 3.13 | 0.45 |
| IR22 | 35 | 1 | 4 | 3 | 100.00 | 3.00 | 10.33 | 1.00 | 3.44 | 3.44 | 0.50 |
| IR22 | 35 | 2 | 1 | 5 | 80.00 | 9.60 | 35.80 | 4.00 | 14.92 | 3.73 | 0.30 |
| IR22 | 35 | 2 | 2 | 3 | 33.33 | 0.33 | 1.00 | 0.33 | 1.00 | 3.00 | 0.67 |
| IR22 | 35 | 2 | 3 | 3 | 100.00 | 2.67 | 10.67 | 0.89 | 3.56 | 4.00 | 0.41 |
| IR22 | 35 | 2 | 4 | 3 | 66.67 | 2.67 | 19.00 | 1.33 | 9.50 | 7.13 | 0.27 |
| IR22 | 35 | 3 | 1 | 5 | 80.00 | 5.20 | 23.60 | 2.17 | 9.83 | 4.54 | 0.48 |
| IR22 | 35 | 3 | 2 | 3 | 33.33 | 0.33 | 0.00 | 0.33 | 0.00 | 0.00 | 0.77 |
| IR22 | 35 | 3 | 3 | 3 | 100.00 | 1.33 | 3.00 | 0.44 | 1.00 | 2.25 | 0.55 |
| IR22 | 35 | 3 | 4 | 3 | 66.67 | 0.67 | 0.00 | 0.33 | 0.00 | 0.00 | 0.42 |
| IR22 | 35 | 4 | 1 | 5 | 80.00 | 4.00 | 13.00 | 1.67 | 5.42 | 3.25 | 0.41 |
| IR22 | 35 | 4 | 2 | 3 | 33.33 | 0.33 | 0.00 | 0.33 | 0.00 | 0.00 | 0.90 |
| IR22 | 35 | 4 | 3 | 3 | 100.00 | 3.33 | 11.33 | 1.11 | 3.78 | 3.40 | 0.48 |
| IR22 | 35 | 4 | 4 | 3 | 66.67 | 1.33 | 2.67 | 0.67 | 1.33 | 2.00 | 0.28 |
| IR22 | 35 | 5 | 1 | 5 | 60.00 | 1.60 | 5.20 | 0.89 | 2.89 | 3.25 | 0.24 |
| IR22 | 35 | 5 | 2 | 3 | 33.33 | 0.33 | 0.00 | 0.33 | 0.00 | 0.00 | 0.80 |
| IR22 | 35 | 5 | 3 | 3 | 66.67 | 0.67 | 2.67 | 0.33 | 1.33 | 4.00 | 0.33 |
| IR22 | 35 | 5 | 4 | 3 | 66.67 | 0.67 | 7.33 | 0.33 | 3.67 | 11.00 | 0.28 |
| IR22 | 35 | 6 | 1 | 5 | 60.00 | 0.80 | 1.40 | 0.44 | 0.78 | 1.75 | 0.35 |
| IR22 | 35 | 6 | 2 | 3 | 33.33 | 0.33 | 0.00 | 0.33 | 0.00 | 0.00 | 0.13 |
| IR22 | 35 | 6 | 3 | 3 | 66.67 | 0.67 | 1.00 | 0.33 | 0.50 | 1.50 | 0.31 |
| IR22 | 35 | 6 | 4 | 3 | 0.00 | 0.00 | 0.00 | 0.37 | 0.43 | 1.08 |  |
| IR22 | 35 | 7 | 1 | 5 | 20.00 | 0.20 | 0.40 | 0.33 | 0.67 | 2.00 | 0.90 |
| IR22 | 35 | 7 | 2 | 3 | 0.00 | 0.00 | 0.00 | 0.33 | 0.67 | 2.00 |  |
| IR22 | 35 | 7 | 3 | 3 | 0.00 | 0.00 | 0.00 | 0.33 | 0.67 | 2.00 |  |
| IR22 | 35 | 7 | 4 | 3 | 0.00 | 0.00 | 0.00 | 0.33 | 0.67 | 2.00 |  |
| IR22 | 35 | 8 | 1 | 5 | 0.00 | 0.00 | 0.00 |  |  |  |  |
| IR22 | 35 | 8 | 2 | 3 | 0.00 | 0.00 | 0.00 |  |  |  |  |
| IR22 | 35 | 8 | 3 | 3 | 0.00 | 0.00 | 0.00 |  |  |  |  |
| IR22 | 35 | 8 | 4 | 3 | 0.00 | 0.00 | 0.00 |  |  |  |  |
| IR22 | 35 | 9 | 1 | 5 | 0.00 | 0.00 | 0.00 |  |  |  |  |
| IR22 | 35 | 9 | 2 | 3 | 0.00 | 0.00 | 0.00 |  |  |  |  |
| IR22 | 35 | 9 | 3 | 3 | 0.00 | 0.00 | 0.00 |  |  |  |  |
| IR22 | 35 | 9 | 4 | 3 | 0.00 | 0.00 | 0.00 |  |  |  |  |
| IR22 | 35 | 10 | 1 | 5 | 0.00 | 0.00 | 0.00 |  |  |  |  |
| IR22 | 35 | 10 | 2 | 3 | 0.00 | 0.00 | 0.00 |  |  |  |  |
| IR22 | 35 | 10 | 3 | 3 | 0.00 | 0.00 | 0.00 |  |  |  |  |
| IR22 | 35 | 10 | 4 | 3 | 0.00 | 0.00 | 0.00 |  |  |  |  |
| IR22 | 35 | 11 | 1 | 5 | 0.00 | 0.00 | 0.00 |  |  |  |  |
| IR22 | 35 | 11 | 2 | 3 | 0.00 | 0.00 | 0.00 |  |  |  |  |
| IR22 | 35 | 11 | 3 | 3 | 0.00 | 0.00 | 0.00 |  |  |  |  |
| IR22 | 35 | 11 | 4 | 3 | 0.00 | 0.00 | 0.00 |  |  |  |  |
| IR22 | 35 | 12 | 1 | 5 | 0.00 | 0.00 | 0.00 |  |  |  |  |
| IR22 | 35 | 12 | 2 | 3 | 0.00 | 0.00 | 0.00 |  |  |  |  |
| IR22 | 35 | 12 | 3 | 3 | 0.00 | 0.00 | 0.00 |  |  |  |  |
| IR22 | 35 | 12 | 4 | 3 | 0.00 | 0.00 | 0.00 |  |  |  |  |
| IR22 | 35 | 13 | 1 | 5 | 0.00 | 0.00 | 0.00 |  |  |  |  |
| IR22 | 35 | 13 | 2 | 3 | 0.00 | 0.00 | 0.00 |  |  |  |  |
| IR22 | 35 | 13 | 3 | 3 | 0.00 | 0.00 | 0.00 |  |  |  |  |
| IR22 | 35 | 13 | 4 | 3 | 0.00 | 0.00 | 0.00 |  |  |  |  |
| IR22 | 35 | 14 | 1 | 5 | 0.00 | 0.00 | 0.00 |  |  |  |  |
| IR22 | 35 | 14 | 2 | 3 | 0.00 | 0.00 | 0.00 |  |  |  |  |
| IR22 | 35 | 14 | 3 | 3 | 0.00 | 0.00 | 0.00 |  |  |  |  |
| IR22 | 35 | 14 | 4 | 3 | 0.00 | 0.00 | 0.00 |  |  |  |  |
| IR22 | 35 | 15 | 1 | 5 | 0.00 | 0.00 | 0.00 |  |  |  |  |
| IR22 | 35 | 15 | 2 | 3 | 0.00 | 0.00 | 0.00 |  |  |  |  |
| IR22 | 35 | 15 | 3 | 3 | 0.00 | 0.00 | 0.00 |  |  |  |  |
| IR22 | 35 | 15 | 4 | 3 | 0.00 | 0.00 | 0.00 |  |  |  |  |
| IR22 | 35 | 16 | 1 | 5 | 0.00 | 0.00 | 0.00 |  |  |  |  |
| IR22 | 35 | 16 | 2 | 3 | 0.00 | 0.00 | 0.00 |  |  |  |  |
| IR22 | 35 | 16 | 3 | 3 | 0.00 | 0.00 | 0.00 |  |  |  |  |
| IR22 | 35 | 16 | 4 | 3 | 0.00 | 0.00 | 0.00 |  |  |  |  |
| IR22 | 35 | 17 | 1 | 5 | 0.00 | 0.00 | 0.00 |  |  |  |  |
| IR22 | 35 | 17 | 2 | 3 | 0.00 | 0.00 | 0.00 |  |  |  |  |
| IR22 | 35 | 17 | 3 | 3 | 0.00 | 0.00 | 0.00 |  |  |  |  |
| IR22 | 35 | 17 | 4 | 3 | 0.00 | 0.00 | 0.00 |  |  |  |  |
| IR22 | 35 | 18 | 1 | 5 | 0.00 | 0.00 | 0.00 |  |  |  |  |
| IR22 | 35 | 18 | 2 | 3 | 0.00 | 0.00 | 0.00 |  |  |  |  |
| IR22 | 35 | 18 | 3 | 3 | 0.00 | 0.00 | 0.00 |  |  |  |  |
| IR22 | 35 | 18 | 4 | 3 | 0.00 | 0.00 | 0.00 |  |  |  |  |
| IR22 | 35 | 19 | 1 | 5 | 0.00 | 0.00 | 0.00 |  |  |  |  |
| IR22 | 35 | 19 | 2 | 3 | 0.00 | 0.00 | 0.00 |  |  |  |  |
| IR22 | 35 | 19 | 3 | 3 | 0.00 | 0.00 | 0.00 |  |  |  |  |
| IR22 | 35 | 19 | 4 | 3 | 0.00 | 0.00 | 0.00 |  |  |  |  |
| IR22 | 35 | 20 | 1 | 5 | 0.00 | 0.00 | 0.00 |  |  |  |  |
| IR22 | 35 | 20 | 2 | 3 | 0.00 | 0.00 | 0.00 |  |  |  |  |
| IR22 | 35 | 20 | 3 | 3 | 0.00 | 0.00 | 0.00 |  |  |  |  |
| IR22 | 35 | 20 | 4 | 3 | 0.00 | 0.00 | 0.00 |  |  |  |  |
| IR62 | 15 | 1 | 1 | 3 | 100.00 | 9.00 | 39.67 | 3.00 | 13.22 | 4.41 | 0.29 |
| IR62 | 15 | 1 | 2 | 3 | 100.00 | 5.33 | 14.33 | 1.78 | 4.78 | 2.69 | 0.15 |
| IR62 | 15 | 1 | 3 | 3 | 100.00 | 6.33 | 27.11 | 2.11 | 9.04 | 4.32 | 0.27 |
| IR62 | 15 | 1 | 4 | 3 | 100.00 | 4.67 | 27.33 | 1.56 | 9.11 | 5.86 | 0.38 |
| IR62 | 15 | 2 | 1 | 3 | 100.00 | 3.33 | 14.00 | 1.11 | 4.67 | 4.20 | 0.28 |
| IR62 | 15 | 2 | 2 | 3 | 100.00 | 5.00 | 14.00 | 1.67 | 4.67 | 2.80 | 0.19 |
| IR62 | 15 | 2 | 3 | 3 | 100.00 | 3.56 | 12.78 | 1.19 | 4.26 | 3.81 | 0.31 |
| IR62 | 15 | 2 | 4 | 3 | 100.00 | 2.33 | 10.33 | 0.78 | 3.44 | 4.43 | 0.47 |
| IR62 | 15 | 3 | 1 | 3 | 100.00 | 2.00 | 6.67 | 0.67 | 2.22 | 3.33 | 0.35 |
| IR62 | 15 | 3 | 2 | 3 | 100.00 | 4.00 | 10.67 | 1.33 | 3.56 | 2.67 | 0.23 |
| IR62 | 15 | 3 | 3 | 3 | 100.00 | 2.56 | 7.11 | 0.85 | 2.37 | 2.80 | 0.35 |
| IR62 | 15 | 3 | 4 | 3 | 100.00 | 1.67 | 4.00 | 0.56 | 1.33 | 2.40 | 0.47 |
| IR62 | 15 | 4 | 1 | 3 | 100.00 | 2.00 | 6.33 | 0.67 | 2.11 | 3.17 | 0.32 |
| IR62 | 15 | 4 | 2 | 3 | 100.00 | 3.67 | 13.33 | 1.22 | 4.44 | 3.64 | 0.15 |
| IR62 | 15 | 4 | 3 | 3 | 100.00 | 2.22 | 6.56 | 0.74 | 2.19 | 2.27 | 0.33 |
| IR62 | 15 | 4 | 4 | 3 | 100.00 | 1.00 | 0.00 | 0.33 | 0.00 | 0.00 | 0.53 |
| IR62 | 15 | 5 | 1 | 3 | 100.00 | 2.67 | 7.67 | 0.89 | 2.56 | 2.88 | 0.60 |
| IR62 | 15 | 5 | 2 | 3 | 100.00 | 1.67 | 2.67 | 0.56 | 0.89 | 1.60 | 0.21 |
| IR62 | 15 | 5 | 3 | 3 | 100.00 | 1.78 | 3.44 | 0.59 | 1.15 | 1.49 | 0.53 |
| IR62 | 15 | 5 | 4 | 3 | 100.00 | 1.00 | 0.00 | 0.33 | 0.00 | 0.00 | 0.77 |
| IR62 | 15 | 6 | 1 | 3 | 100.00 | 2.33 | 10.00 | 0.78 | 3.33 | 4.29 | 0.34 |
| IR62 | 15 | 6 | 2 | 3 | 100.00 | 2.00 | 4.33 | 0.67 | 1.44 | 2.17 | 0.29 |
| IR62 | 15 | 6 | 3 | 3 | 100.00 | 1.78 | 4.78 | 0.59 | 1.59 | 2.15 | 0.46 |
| IR62 | 15 | 6 | 4 | 3 | 100.00 | 1.00 | 0.00 | 0.33 | 0.00 | 0.00 | 0.59 |
| IR62 | 15 | 7 | 1 | 3 | 100.00 | 1.67 | 4.33 | 0.56 | 1.44 | 2.60 | 0.36 |
| IR62 | 15 | 7 | 2 | 3 | 100.00 | 2.00 | 1.67 | 0.67 | 0.56 | 0.83 | 0.17 |
| IR62 | 15 | 7 | 3 | 3 | 100.00 | 1.56 | 3.33 | 0.52 | 1.11 | 2.48 | 0.37 |
| IR62 | 15 | 7 | 4 | 3 | 100.00 | 1.00 | 4.00 | 0.33 | 1.33 | 4.00 | 0.59 |
| IR62 | 15 | 8 | 1 | 3 | 66.67 | 1.00 | 8.00 | 0.50 | 4.00 | 8.00 | 0.25 |
| IR62 | 15 | 8 | 2 | 3 | 100.00 | 1.67 | 3.00 | 0.56 | 1.00 | 1.80 | 0.27 |
| IR62 | 15 | 8 | 3 | 3 | 77.78 | 1.11 | 3.78 | 0.46 | 1.72 | 3.43 | 0.35 |
| IR62 | 15 | 8 | 4 | 3 | 66.67 | 0.67 | 0.33 | 0.33 | 0.17 | 0.50 | 0.51 |
| IR62 | 15 | 9 | 1 | 3 | 66.67 | 0.67 | 2.00 | 0.33 | 1.00 | 3.00 | 0.30 |
| IR62 | 15 | 9 | 2 | 3 | 66.67 | 0.67 | 0.00 | 0.33 | 0.00 | 0.00 | 0.15 |
| IR62 | 15 | 9 | 3 | 3 | 44.44 | 0.44 | 0.67 | 0.33 | 0.50 | 1.50 | 0.15 |
| IR62 | 15 | 9 | 4 | 3 | 0.00 | 0.00 | 0.00 | 0.33 | 0.50 | 1.50 |  |
| IR62 | 15 | 10 | 1 | 3 | 66.67 | 0.67 | 0.00 | 0.33 | 0.00 | 0.00 | 0.48 |
| IR62 | 15 | 10 | 2 | 3 | 66.67 | 0.67 | 3.33 | 0.33 | 1.67 | 5.00 | 0.21 |
| IR62 | 15 | 10 | 3 | 3 | 44.44 | 0.44 | 1.11 | 0.33 | 0.83 | 2.50 | 0.23 |
| IR62 | 15 | 10 | 4 | 3 | 0.00 | 0.00 | 0.00 | 0.33 | 0.83 | 2.50 |  |
| IR62 | 15 | 11 | 1 | 3 | 66.67 | 0.67 | 3.33 | 0.33 | 1.67 | 5.00 | 0.43 |
| IR62 | 15 | 11 | 2 | 3 | 33.33 | 0.33 | 0.00 | 0.33 | 0.00 | 0.00 | 0.15 |
| IR62 | 15 | 11 | 3 | 3 | 33.33 | 0.33 | 1.11 | 0.33 | 0.83 | 2.50 | 0.19 |
| IR62 | 15 | 11 | 4 | 3 | 0.00 | 0.00 | 0.00 | 0.33 | 0.83 | 2.50 |  |
| IR62 | 15 | 12 | 1 | 3 | 66.67 | 1.67 | 2.67 | 0.83 | 1.33 | 1.60 | 0.46 |
| IR62 | 15 | 12 | 2 | 3 | 33.33 | 0.33 | 0.00 | 0.33 | 0.00 | 0.00 | 0.13 |
| IR62 | 15 | 12 | 3 | 3 | 33.33 | 0.67 | 0.89 | 0.58 | 0.67 | 0.80 | 0.19 |
| IR62 | 15 | 12 | 4 | 3 | 0.00 | 0.00 | 0.00 | 0.58 | 0.67 | 0.80 |  |
| IR62 | 15 | 13 | 1 | 3 | 66.67 | 0.67 | 0.00 | 0.33 | 0.00 | 0.00 | 0.43 |
| IR62 | 15 | 13 | 2 | 3 | 0.00 | 0.00 | 0.00 | 0.33 | 0.00 | 0.00 |  |
| IR62 | 15 | 13 | 3 | 3 | 22.22 | 0.22 | 0.00 | 0.33 | 0.00 | 0.00 | 0.14 |
| IR62 | 15 | 13 | 4 | 3 | 0.00 | 0.00 | 0.00 | 0.33 | 0.00 | 0.00 |  |
| IR62 | 15 | 14 | 1 | 3 | 66.67 | 0.67 | 0.00 | 0.33 | 0.00 | 0.00 | 0.49 |
| IR62 | 15 | 14 | 2 | 3 | 0.00 | 0.00 | 0.00 | 0.33 | 0.00 | 0.00 |  |
| IR62 | 15 | 14 | 3 | 3 | 22.22 | 0.22 | 0.00 | 0.33 | 0.00 | 0.00 | 0.16 |
| IR62 | 15 | 14 | 4 | 3 | 0.00 | 0.00 | 0.00 | 0.33 | 0.00 | 0.00 |  |
| IR62 | 15 | 15 | 1 | 3 | 66.67 | 0.67 | 0.00 | 0.33 | 0.00 | 0.00 | 0.64 |
| IR62 | 15 | 15 | 2 | 3 | 0.00 | 0.00 | 0.00 | 0.33 | 0.00 | 0.00 |  |
| IR62 | 15 | 15 | 3 | 3 | 22.22 | 0.22 | 0.00 | 0.33 | 0.00 | 0.00 | 0.21 |
| IR62 | 15 | 15 | 4 | 3 | 0.00 | 0.00 | 0.00 | 0.33 | 0.00 | 0.00 |  |
| IR62 | 15 | 16 | 1 | 3 | 66.67 | 0.67 | 0.00 | 0.33 | 0.00 | 0.00 | 0.59 |
| IR62 | 15 | 16 | 2 | 3 | 0.00 | 0.00 | 0.00 | 0.33 | 0.00 | 0.00 |  |
| IR62 | 15 | 16 | 3 | 3 | 22.22 | 0.22 | 0.00 | 0.33 | 0.00 | 0.00 | 0.20 |
| IR62 | 15 | 16 | 4 | 3 | 0.00 | 0.00 | 0.00 | 0.33 | 0.00 | 0.00 |  |
| IR62 | 15 | 17 | 1 | 3 | 33.33 | 0.33 | 0.00 | 0.33 | 0.00 | 0.00 | 0.42 |
| IR62 | 15 | 17 | 2 | 3 | 0.00 | 0.00 | 0.00 | 0.33 | 0.00 | 0.00 |  |
| IR62 | 15 | 17 | 3 | 3 | 11.11 | 0.11 | 0.00 | 0.33 | 0.00 | 0.00 | 0.14 |
| IR62 | 15 | 17 | 4 | 3 | 0.00 | 0.00 | 0.00 | 0.33 | 0.00 | 0.00 |  |
| IR62 | 15 | 18 | 1 | 3 | 0.00 | 0.00 | 0.00 |  |  |  |  |
| IR62 | 15 | 18 | 2 | 3 | 0.00 | 0.00 | 0.00 |  |  |  |  |
| IR62 | 15 | 18 | 3 | 3 | 0.00 | 0.00 | 0.00 |  |  |  |  |
| IR62 | 15 | 18 | 4 | 3 | 0.00 | 0.00 | 0.00 |  |  |  |  |
| IR62 | 15 | 19 | 1 | 3 | 0.00 | 0.00 | 0.00 |  |  |  |  |
| IR62 | 15 | 19 | 2 | 3 | 0.00 | 0.00 | 0.00 |  |  |  |  |
| IR62 | 15 | 19 | 3 | 3 | 0.00 | 0.00 | 0.00 |  |  |  |  |
| IR62 | 15 | 19 | 4 | 3 | 0.00 | 0.00 | 0.00 |  |  |  |  |
| IR62 | 15 | 20 | 1 | 3 | 0.00 | 0.00 | 0.00 |  |  |  |  |
| IR62 | 15 | 20 | 2 | 3 | 0.00 | 0.00 | 0.00 |  |  |  |  |
| IR62 | 15 | 20 | 3 | 3 | 0.00 | 0.00 | 0.00 |  |  |  |  |
| IR62 | 15 | 20 | 4 | 3 | 0.00 | 0.00 | 0.00 |  |  |  |  |
| IR62 | 20 | 1 | 1 | 3 | 100.00 | 9.50 | 34.17 | 3.17 | 11.39 | 3.61 | 0.36 |
| IR62 | 20 | 1 | 2 | 3 | 100.00 | 9.50 | 34.17 | 3.17 | 11.39 | 3.61 | 0.36 |
| IR62 | 20 | 1 | 3 | 3 | 100.00 | 10.00 | 33.00 | 3.33 | 11.00 | 3.30 | 0.29 |
| IR62 | 20 | 1 | 4 | 3 | 100.00 | 9.00 | 35.33 | 3.00 | 11.78 | 3.93 | 0.43 |
| IR62 | 20 | 2 | 1 | 3 | 83.33 | 1.83 | 6.00 | 0.75 | 2.31 | 3.18 | 0.34 |
| IR62 | 20 | 2 | 2 | 3 | 83.33 | 1.83 | 6.00 | 0.75 | 2.31 | 3.18 | 0.34 |
| IR62 | 20 | 2 | 3 | 3 | 100.00 | 2.00 | 8.33 | 0.67 | 2.78 | 4.17 | 0.36 |
| IR62 | 20 | 2 | 4 | 3 | 66.67 | 1.67 | 3.67 | 0.83 | 1.83 | 2.20 | 0.33 |
| IR62 | 20 | 3 | 1 | 3 | 66.67 | 1.67 | 5.50 | 0.67 | 2.28 | 3.61 | 0.26 |
| IR62 | 20 | 3 | 2 | 3 | 66.67 | 1.67 | 5.50 | 0.67 | 2.28 | 3.61 | 0.26 |
| IR62 | 20 | 3 | 3 | 3 | 100.00 | 3.00 | 9.67 | 1.00 | 3.22 | 3.22 | 0.36 |
| IR62 | 20 | 3 | 4 | 3 | 33.33 | 0.33 | 1.33 | 0.33 | 1.33 | 4.00 | 0.15 |
| IR62 | 20 | 4 | 1 | 3 | 33.33 | 1.00 | 3.50 | 1.00 | 3.50 | 2.10 | 0.17 |
| IR62 | 20 | 4 | 2 | 3 | 33.33 | 1.00 | 3.50 | 1.00 | 3.50 | 2.10 | 0.17 |
| IR62 | 20 | 4 | 3 | 3 | 33.33 | 1.67 | 7.00 | 1.67 | 7.00 | 4.20 | 0.14 |
| IR62 | 20 | 4 | 4 | 3 | 33.33 | 0.33 | 0.00 | 0.33 | 0.00 | 0.00 | 0.21 |
| IR62 | 20 | 5 | 1 | 3 | 33.33 | 1.17 | 3.83 | 1.17 | 3.83 | 1.92 | 0.12 |
| IR62 | 20 | 5 | 2 | 3 | 33.33 | 1.17 | 3.83 | 1.17 | 3.83 | 1.92 | 0.12 |
| IR62 | 20 | 5 | 3 | 3 | 33.33 | 2.00 | 7.67 | 2.00 | 7.67 | 3.83 | 0.90 |
| IR62 | 20 | 5 | 4 | 3 | 33.33 | 0.33 | 0.00 | 0.33 | 0.00 | 0.00 | 0.16 |
| IR62 | 20 | 6 | 1 | 3 | 33.33 | 1.17 | 3.17 | 1.17 | 3.17 | 1.58 | 0.19 |
| IR62 | 20 | 6 | 2 | 3 | 33.33 | 1.17 | 3.17 | 1.17 | 3.17 | 1.58 | 0.19 |
| IR62 | 20 | 6 | 3 | 3 | 33.33 | 2.00 | 6.33 | 2.00 | 6.33 | 3.17 | 0.15 |
| IR62 | 20 | 6 | 4 | 3 | 33.33 | 0.33 | 0.00 | 0.33 | 0.00 | 0.00 | 0.23 |
| IR62 | 20 | 7 | 1 | 3 | 11.11 | 0.56 | 2.56 |  |  |  | 0.64 |
| IR62 | 20 | 7 | 2 | 3 | 0.00 | 0.00 | 0.00 |  |  |  |  |
| IR62 | 20 | 7 | 3 | 3 | 33.33 | 1.67 | 7.67 | 1.67 | 7.67 | 4.60 | 0.19 |
| IR62 | 20 | 7 | 4 | 3 | 0.00 | 0.00 | 0.00 |  |  |  |  |
| IR62 | 20 | 8 | 1 | 3 | 0.00 | 0.00 | 0.00 |  |  |  |  |
| IR62 | 20 | 8 | 2 | 3 | 0.00 | 0.00 | 0.00 |  |  |  |  |
| IR62 | 20 | 8 | 3 | 3 | 0.00 | 0.00 | 0.00 |  |  |  |  |
| IR62 | 20 | 8 | 4 | 3 | 0.00 | 0.00 | 0.00 |  |  |  |  |
| IR62 | 20 | 9 | 1 | 3 | 0.00 | 0.00 | 0.00 |  |  |  |  |
| IR62 | 20 | 9 | 2 | 3 | 0.00 | 0.00 | 0.00 |  |  |  |  |
| IR62 | 20 | 9 | 3 | 3 | 0.00 | 0.00 | 0.00 |  |  |  |  |
| IR62 | 20 | 9 | 4 | 3 | 0.00 | 0.00 | 0.00 |  |  |  |  |
| IR62 | 20 | 10 | 1 | 3 | 0.00 | 0.00 | 0.00 |  |  |  |  |
| IR62 | 20 | 10 | 2 | 3 | 0.00 | 0.00 | 0.00 |  |  |  |  |
| IR62 | 20 | 10 | 3 | 3 | 0.00 | 0.00 | 0.00 |  |  |  |  |
| IR62 | 20 | 10 | 4 | 3 | 0.00 | 0.00 | 0.00 |  |  |  |  |
| IR62 | 20 | 11 | 1 | 3 | 0.00 | 0.00 | 0.00 |  |  |  |  |
| IR62 | 20 | 11 | 2 | 3 | 0.00 | 0.00 | 0.00 |  |  |  |  |
| IR62 | 20 | 11 | 3 | 3 | 0.00 | 0.00 | 0.00 |  |  |  |  |
| IR62 | 20 | 11 | 4 | 3 | 0.00 | 0.00 | 0.00 |  |  |  |  |
| IR62 | 20 | 12 | 1 | 3 | 0.00 | 0.00 | 0.00 |  |  |  |  |
| IR62 | 20 | 12 | 2 | 3 | 0.00 | 0.00 | 0.00 |  |  |  |  |
| IR62 | 20 | 12 | 3 | 3 | 0.00 | 0.00 | 0.00 |  |  |  |  |
| IR62 | 20 | 12 | 4 | 3 | 0.00 | 0.00 | 0.00 |  |  |  |  |
| IR62 | 20 | 13 | 1 | 3 | 0.00 | 0.00 | 0.00 |  |  |  |  |
| IR62 | 20 | 13 | 2 | 3 | 0.00 | 0.00 | 0.00 |  |  |  |  |
| IR62 | 20 | 13 | 3 | 3 | 0.00 | 0.00 | 0.00 |  |  |  |  |
| IR62 | 20 | 13 | 4 | 3 | 0.00 | 0.00 | 0.00 |  |  |  |  |
| IR62 | 20 | 14 | 1 | 3 | 0.00 | 0.00 | 0.00 |  |  |  |  |
| IR62 | 20 | 14 | 2 | 3 | 0.00 | 0.00 | 0.00 |  |  |  |  |
| IR62 | 20 | 14 | 3 | 3 | 0.00 | 0.00 | 0.00 |  |  |  |  |
| IR62 | 20 | 14 | 4 | 3 | 0.00 | 0.00 | 0.00 |  |  |  |  |
| IR62 | 20 | 15 | 1 | 3 | 0.00 | 0.00 | 0.00 |  |  |  |  |
| IR62 | 20 | 15 | 2 | 3 | 0.00 | 0.00 | 0.00 |  |  |  |  |
| IR62 | 20 | 15 | 3 | 3 | 0.00 | 0.00 | 0.00 |  |  |  |  |
| IR62 | 20 | 15 | 4 | 3 | 0.00 | 0.00 | 0.00 |  |  |  |  |
| IR62 | 20 | 16 | 1 | 3 | 0.00 | 0.00 | 0.00 |  |  |  |  |
| IR62 | 20 | 16 | 2 | 3 | 0.00 | 0.00 | 0.00 |  |  |  |  |
| IR62 | 20 | 16 | 3 | 3 | 0.00 | 0.00 | 0.00 |  |  |  |  |
| IR62 | 20 | 16 | 4 | 3 | 0.00 | 0.00 | 0.00 |  |  |  |  |
| IR62 | 20 | 17 | 1 | 3 | 0.00 | 0.00 | 0.00 |  |  |  |  |
| IR62 | 20 | 17 | 2 | 3 | 0.00 | 0.00 | 0.00 |  |  |  |  |
| IR62 | 20 | 17 | 3 | 3 | 0.00 | 0.00 | 0.00 |  |  |  |  |
| IR62 | 20 | 17 | 4 | 3 | 0.00 | 0.00 | 0.00 |  |  |  |  |
| IR62 | 20 | 18 | 1 | 3 | 0.00 | 0.00 | 0.00 |  |  |  |  |
| IR62 | 20 | 18 | 2 | 3 | 0.00 | 0.00 | 0.00 |  |  |  |  |
| IR62 | 20 | 18 | 3 | 3 | 0.00 | 0.00 | 0.00 |  |  |  |  |
| IR62 | 20 | 18 | 4 | 3 | 0.00 | 0.00 | 0.00 |  |  |  |  |
| IR62 | 20 | 19 | 1 | 3 | 0.00 | 0.00 | 0.00 |  |  |  |  |
| IR62 | 20 | 19 | 2 | 3 | 0.00 | 0.00 | 0.00 |  |  |  |  |
| IR62 | 20 | 19 | 3 | 3 | 0.00 | 0.00 | 0.00 |  |  |  |  |
| IR62 | 20 | 19 | 4 | 3 | 0.00 | 0.00 | 0.00 |  |  |  |  |
| IR62 | 20 | 20 | 1 | 3 | 0.00 | 0.00 | 0.00 |  |  |  |  |
| IR62 | 20 | 20 | 2 | 3 | 0.00 | 0.00 | 0.00 |  |  |  |  |
| IR62 | 20 | 20 | 3 | 3 | 0.00 | 0.00 | 0.00 |  |  |  |  |
| IR62 | 20 | 20 | 4 | 3 | 0.00 | 0.00 | 0.00 |  |  |  |  |
| IR62 | 25 | 1 | 1 | 3 | 100.00 | 5.50 | 16.67 | 1.83 | 5.56 | 3.03 | 0.25 |
| IR62 | 25 | 1 | 2 | 3 | 100.00 | 5.50 | 16.67 | 1.83 | 5.56 | 3.03 | 0.25 |
| IR62 | 25 | 1 | 3 | 3 | 100.00 | 5.67 | 16.33 | 1.89 | 5.44 | 2.88 | 0.23 |
| IR62 | 25 | 1 | 4 | 3 | 100.00 | 5.33 | 17.00 | 1.78 | 5.67 | 3.19 | 0.27 |
| IR62 | 25 | 2 | 1 | 3 | 83.33 | 1.33 | 2.50 | 0.53 | 1.00 | 1.90 | 0.21 |
| IR62 | 25 | 2 | 2 | 3 | 83.33 | 1.33 | 2.50 | 0.53 | 1.00 | 1.90 | 0.21 |
| IR62 | 25 | 2 | 3 | 3 | 66.67 | 1.00 | 2.00 | 0.50 | 1.00 | 2.00 | 0.17 |
| IR62 | 25 | 2 | 4 | 3 | 100.00 | 1.67 | 3.00 | 0.56 | 1.00 | 1.80 | 0.25 |
| IR62 | 25 | 3 | 1 | 3 | 66.67 | 0.67 | 0.17 | 0.33 | 0.08 | 0.25 | 0.23 |
| IR62 | 25 | 3 | 2 | 3 | 66.67 | 0.67 | 0.17 | 0.33 | 0.08 | 0.25 | 0.23 |
| IR62 | 25 | 3 | 3 | 3 | 66.67 | 0.67 | 0.00 | 0.33 | 0.00 | 0.00 | 0.23 |
| IR62 | 25 | 3 | 4 | 3 | 66.67 | 0.67 | 0.33 | 0.33 | 0.17 | 0.50 | 0.22 |
| IR62 | 25 | 4 | 1 | 3 | 33.33 | 0.33 | 0.00 | 0.33 | 0.00 | 0.00 | 0.13 |
| IR62 | 25 | 4 | 2 | 3 | 33.33 | 0.33 | 0.00 | 0.33 | 0.00 | 0.00 | 0.13 |
| IR62 | 25 | 4 | 3 | 3 | 33.33 | 0.33 | 0.00 | 0.33 | 0.00 | 0.00 | 0.12 |
| IR62 | 25 | 4 | 4 | 3 | 33.33 | 0.33 | 0.00 | 0.33 | 0.00 | 0.00 | 0.15 |
| IR62 | 25 | 5 | 1 | 3 | 33.33 | 0.33 | 0.83 | 0.33 | 0.83 | 2.50 | 0.13 |
| IR62 | 25 | 5 | 2 | 3 | 33.33 | 0.33 | 0.83 | 0.33 | 0.83 | 2.50 | 0.13 |
| IR62 | 25 | 5 | 3 | 3 | 33.33 | 0.33 | 0.00 | 0.33 | 0.00 | 0.00 | 0.14 |
| IR62 | 25 | 5 | 4 | 3 | 33.33 | 0.33 | 1.67 | 0.33 | 1.67 | 5.00 | 0.11 |
| IR62 | 25 | 6 | 1 | 3 | 11.11 | 0.33 | 0.89 |  |  |  | 0.59 |
| IR62 | 25 | 6 | 2 | 3 | 0.00 | 0.00 | 0.00 |  |  |  |  |
| IR62 | 25 | 6 | 3 | 3 | 0.00 | 0.00 | 0.00 |  |  |  |  |
| IR62 | 25 | 6 | 4 | 3 | 33.33 | 1.00 | 2.67 | 1.00 | 2.67 | 2.67 | 0.18 |
| IR62 | 25 | 7 | 1 | 3 | 0.00 | 0.00 | 0.00 |  |  |  |  |
| IR62 | 25 | 7 | 2 | 3 | 0.00 | 0.00 | 0.00 |  |  |  |  |
| IR62 | 25 | 7 | 3 | 3 | 0.00 | 0.00 | 0.00 |  |  |  |  |
| IR62 | 25 | 7 | 4 | 3 | 0.00 | 0.00 | 0.00 |  |  |  |  |
| IR62 | 25 | 8 | 1 | 3 | 0.00 | 0.00 | 0.00 |  |  |  |  |
| IR62 | 25 | 8 | 2 | 3 | 0.00 | 0.00 | 0.00 |  |  |  |  |
| IR62 | 25 | 8 | 3 | 3 | 0.00 | 0.00 | 0.00 |  |  |  |  |
| IR62 | 25 | 8 | 4 | 3 | 0.00 | 0.00 | 0.00 |  |  |  |  |
| IR62 | 25 | 9 | 1 | 3 | 0.00 | 0.00 | 0.00 |  |  |  |  |
| IR62 | 25 | 9 | 2 | 3 | 0.00 | 0.00 | 0.00 |  |  |  |  |
| IR62 | 25 | 9 | 3 | 3 | 0.00 | 0.00 | 0.00 |  |  |  |  |
| IR62 | 25 | 9 | 4 | 3 | 0.00 | 0.00 | 0.00 |  |  |  |  |
| IR62 | 25 | 10 | 1 | 3 | 0.00 | 0.00 | 0.00 |  |  |  |  |
| IR62 | 25 | 10 | 2 | 3 | 0.00 | 0.00 | 0.00 |  |  |  |  |
| IR62 | 25 | 10 | 3 | 3 | 0.00 | 0.00 | 0.00 |  |  |  |  |
| IR62 | 25 | 10 | 4 | 3 | 0.00 | 0.00 | 0.00 |  |  |  |  |
| IR62 | 25 | 11 | 1 | 3 | 0.00 | 0.00 | 0.00 |  |  |  |  |
| IR62 | 25 | 11 | 2 | 3 | 0.00 | 0.00 | 0.00 |  |  |  |  |
| IR62 | 25 | 11 | 3 | 3 | 0.00 | 0.00 | 0.00 |  |  |  |  |
| IR62 | 25 | 11 | 4 | 3 | 0.00 | 0.00 | 0.00 |  |  |  |  |
| IR62 | 25 | 12 | 1 | 3 | 0.00 | 0.00 | 0.00 |  |  |  |  |
| IR62 | 25 | 12 | 2 | 3 | 0.00 | 0.00 | 0.00 |  |  |  |  |
| IR62 | 25 | 12 | 3 | 3 | 0.00 | 0.00 | 0.00 |  |  |  |  |
| IR62 | 25 | 12 | 4 | 3 | 0.00 | 0.00 | 0.00 |  |  |  |  |
| IR62 | 25 | 13 | 1 | 3 | 0.00 | 0.00 | 0.00 |  |  |  |  |
| IR62 | 25 | 13 | 2 | 3 | 0.00 | 0.00 | 0.00 |  |  |  |  |
| IR62 | 25 | 13 | 3 | 3 | 0.00 | 0.00 | 0.00 |  |  |  |  |
| IR62 | 25 | 13 | 4 | 3 | 0.00 | 0.00 | 0.00 |  |  |  |  |
| IR62 | 25 | 14 | 1 | 3 | 0.00 | 0.00 | 0.00 |  |  |  |  |
| IR62 | 25 | 14 | 2 | 3 | 0.00 | 0.00 | 0.00 |  |  |  |  |
| IR62 | 25 | 14 | 3 | 3 | 0.00 | 0.00 | 0.00 |  |  |  |  |
| IR62 | 25 | 14 | 4 | 3 | 0.00 | 0.00 | 0.00 |  |  |  |  |
| IR62 | 25 | 15 | 1 | 3 | 0.00 | 0.00 | 0.00 |  |  |  |  |
| IR62 | 25 | 15 | 2 | 3 | 0.00 | 0.00 | 0.00 |  |  |  |  |
| IR62 | 25 | 15 | 3 | 3 | 0.00 | 0.00 | 0.00 |  |  |  |  |
| IR62 | 25 | 15 | 4 | 3 | 0.00 | 0.00 | 0.00 |  |  |  |  |
| IR62 | 25 | 16 | 1 | 3 | 0.00 | 0.00 | 0.00 |  |  |  |  |
| IR62 | 25 | 16 | 2 | 3 | 0.00 | 0.00 | 0.00 |  |  |  |  |
| IR62 | 25 | 16 | 3 | 3 | 0.00 | 0.00 | 0.00 |  |  |  |  |
| IR62 | 25 | 16 | 4 | 3 | 0.00 | 0.00 | 0.00 |  |  |  |  |
| IR62 | 30 | 1 | 1 | 3 | 100.00 | 9.33 | 26.67 | 3.11 | 8.89 | 2.87 | 0.33 |
| IR62 | 30 | 1 | 2 | 3 | 100.00 | 9.33 | 26.67 | 3.11 | 8.89 | 2.87 | 0.33 |
| IR62 | 30 | 1 | 3 | 3 | 100.00 | 8.00 | 23.67 | 2.67 | 7.89 | 2.96 | 0.28 |
| IR62 | 30 | 1 | 4 | 3 | 100.00 | 10.67 | 29.67 | 3.56 | 9.89 | 2.78 | 0.32 |
| IR62 | 30 | 2 | 1 | 3 | 100.00 | 2.67 | 7.50 | 0.89 | 2.50 | 2.82 | 0.34 |
| IR62 | 30 | 2 | 2 | 3 | 100.00 | 2.67 | 7.50 | 0.89 | 2.50 | 2.82 | 0.34 |
| IR62 | 30 | 2 | 3 | 3 | 100.00 | 2.33 | 6.67 | 0.78 | 2.22 | 2.86 | 0.31 |
| IR62 | 30 | 2 | 4 | 3 | 100.00 | 3.00 | 8.33 | 1.00 | 2.78 | 2.78 | 0.36 |
| IR62 | 30 | 3 | 1 | 3 | 50.00 | 4.33 | 18.50 | 3.08 | 13.67 | 4.34 | 0.23 |
| IR62 | 30 | 3 | 2 | 3 | 50.00 | 4.33 | 18.50 | 3.08 | 13.67 | 4.34 | 0.23 |
| IR62 | 30 | 3 | 3 | 3 | 33.33 | 3.67 | 17.67 | 3.67 | 17.67 | 4.82 | 0.13 |
| IR62 | 30 | 3 | 4 | 3 | 66.67 | 5.00 | 19.33 | 2.50 | 9.67 | 3.87 | 0.33 |
| IR62 | 30 | 4 | 1 | 3 | 50.00 | 2.17 | 9.83 | 1.50 | 7.00 | 4.63 | 0.18 |
| IR62 | 30 | 4 | 2 | 3 | 50.00 | 2.17 | 9.83 | 1.50 | 7.00 | 4.63 | 0.18 |
| IR62 | 30 | 4 | 3 | 3 | 33.33 | 1.67 | 8.33 | 1.67 | 8.33 | 5.00 | 0.11 |
| IR62 | 30 | 4 | 4 | 3 | 66.67 | 2.67 | 11.33 | 1.33 | 5.67 | 4.25 | 0.24 |
| IR62 | 30 | 5 | 1 | 3 | 50.00 | 2.00 | 9.33 | 1.08 | 5.08 | 4.82 | 0.33 |
| IR62 | 30 | 5 | 2 | 3 | 50.00 | 2.00 | 9.33 | 1.08 | 5.08 | 4.82 | 0.33 |
| IR62 | 30 | 5 | 3 | 3 | 33.33 | 0.33 | 1.67 | 0.33 | 1.67 | 5.00 | 0.19 |
| IR62 | 30 | 5 | 4 | 3 | 66.67 | 3.67 | 17.00 | 1.83 | 8.50 | 4.64 | 0.47 |
| IR62 | 30 | 6 | 1 | 3 | 50.00 | 2.67 | 8.33 | 1.42 | 4.17 | 1.67 | 0.37 |
| IR62 | 30 | 6 | 2 | 3 | 50.00 | 2.67 | 8.33 | 1.42 | 4.17 | 1.67 | 0.37 |
| IR62 | 30 | 6 | 3 | 3 | 33.33 | 0.33 | 0.00 | 0.33 | 0.00 | 0.00 | 0.22 |
| IR62 | 30 | 6 | 4 | 3 | 66.67 | 5.00 | 16.67 | 2.50 | 8.33 | 3.33 | 0.39 |
| IR62 | 30 | 7 | 1 | 3 | 16.67 | 0.17 | 0.50 |  |  |  | 0.13 |
| IR62 | 30 | 7 | 2 | 3 | 16.67 | 0.17 | 0.50 |  |  |  | 0.13 |
| IR62 | 30 | 7 | 3 | 3 | 0.00 | 0.00 | 0.00 |  |  |  |  |
| IR62 | 30 | 7 | 4 | 3 | 33.33 | 0.33 | 1.00 | 0.33 | 1.00 | 3.00 | 0.27 |
| IR62 | 30 | 8 | 1 | 3 | 16.67 | 0.17 | 0.00 |  |  |  | 0.88 |
| IR62 | 30 | 8 | 2 | 3 | 16.67 | 0.17 | 0.00 |  |  |  | 0.88 |
| IR62 | 30 | 8 | 3 | 3 | 0.00 | 0.00 | 0.00 |  |  |  |  |
| IR62 | 30 | 8 | 4 | 3 | 33.33 | 0.33 | 0.00 | 0.33 | 0.00 | 0.00 | 0.18 |
| IR62 | 30 | 9 | 1 | 3 | 16.67 | 1.83 | 6.83 |  |  |  | 0.14 |
| IR62 | 30 | 9 | 2 | 3 | 16.67 | 1.83 | 6.83 |  |  |  | 0.14 |
| IR62 | 30 | 9 | 3 | 3 | 0.00 | 0.00 | 0.00 |  |  |  |  |
| IR62 | 30 | 9 | 4 | 3 | 33.33 | 3.67 | 13.67 | 3.67 | 13.67 | 3.73 | 0.28 |
| IR62 | 30 | 10 | 1 | 3 | 16.67 | 0.33 | 0.83 |  |  |  | 0.97 |
| IR62 | 30 | 10 | 2 | 3 | 16.67 | 0.33 | 0.83 |  |  |  | 0.97 |
| IR62 | 30 | 10 | 3 | 3 | 0.00 | 0.00 | 0.00 |  |  |  |  |
| IR62 | 30 | 10 | 4 | 3 | 33.33 | 0.67 | 1.67 | 0.67 | 1.67 | 2.50 | 0.19 |
| IR62 | 30 | 11 | 1 | 3 | 16.67 | 0.33 | 0.67 |  |  |  | 0.82 |
| IR62 | 30 | 11 | 2 | 3 | 16.67 | 0.33 | 0.67 |  |  |  | 0.82 |
| IR62 | 30 | 11 | 3 | 3 | 0.00 | 0.00 | 0.00 |  |  |  |  |
| IR62 | 30 | 11 | 4 | 3 | 33.33 | 0.67 | 1.33 | 0.67 | 1.33 | 2.00 | 0.16 |
| IR62 | 30 | 12 | 1 | 3 | 0.00 | 0.00 | 0.00 |  |  |  |  |
| IR62 | 30 | 12 | 2 | 3 | 0.00 | 0.00 | 0.00 |  |  |  |  |
| IR62 | 30 | 12 | 3 | 3 | 0.00 | 0.00 | 0.00 |  |  |  |  |
| IR62 | 30 | 12 | 4 | 3 | 0.00 | 0.00 | 0.00 |  |  |  |  |
| IR62 | 30 | 13 | 1 | 3 | 0.00 | 0.00 | 0.00 |  |  |  |  |
| IR62 | 30 | 13 | 2 | 3 | 0.00 | 0.00 | 0.00 |  |  |  |  |
| IR62 | 30 | 13 | 3 | 3 | 0.00 | 0.00 | 0.00 |  |  |  |  |
| IR62 | 30 | 13 | 4 | 3 | 0.00 | 0.00 | 0.00 |  |  |  |  |
| IR62 | 30 | 14 | 1 | 3 | 0.00 | 0.00 | 0.00 |  |  |  |  |
| IR62 | 30 | 14 | 2 | 3 | 0.00 | 0.00 | 0.00 |  |  |  |  |
| IR62 | 30 | 14 | 3 | 3 | 0.00 | 0.00 | 0.00 |  |  |  |  |
| IR62 | 30 | 14 | 4 | 3 | 0.00 | 0.00 | 0.00 |  |  |  |  |
| IR62 | 30 | 15 | 1 | 3 | 0.00 | 0.00 | 0.00 |  |  |  |  |
| IR62 | 30 | 15 | 2 | 3 | 0.00 | 0.00 | 0.00 |  |  |  |  |
| IR62 | 30 | 15 | 3 | 3 | 0.00 | 0.00 | 0.00 |  |  |  |  |
| IR62 | 30 | 15 | 4 | 3 | 0.00 | 0.00 | 0.00 |  |  |  |  |
| IR62 | 30 | 16 | 1 | 3 | 0.00 | 0.00 | 0.00 |  |  |  |  |
| IR62 | 30 | 16 | 2 | 3 | 0.00 | 0.00 | 0.00 |  |  |  |  |
| IR62 | 30 | 16 | 3 | 3 | 0.00 | 0.00 | 0.00 |  |  |  |  |
| IR62 | 30 | 16 | 4 | 3 | 0.00 | 0.00 | 0.00 |  |  |  |  |
| IR62 | 35 | 1 | 1 | 3 | 100.00 | 11.11 | 30.00 | 3.70 | 10.00 | 2.77 | 0.34 |
| IR62 | 35 | 1 | 2 | 3 | 100.00 | 18.33 | 48.33 | 6.11 | 16.11 | 2.64 | 0.17 |
| IR62 | 35 | 1 | 3 | 3 | 100.00 | 5.00 | 15.00 | 1.67 | 5.00 | 3.00 | 0.45 |
| IR62 | 35 | 1 | 4 | 3 | 100.00 | 10.00 | 26.67 | 3.33 | 8.89 | 2.67 | 0.39 |
| IR62 | 35 | 2 | 1 | 3 | 100.00 | 3.89 | 15.67 | 1.30 | 5.22 | 5.04 | 0.35 |
| IR62 | 35 | 2 | 2 | 3 | 100.00 | 8.33 | 25.33 | 2.78 | 8.44 | 3.04 | 0.22 |
| IR62 | 35 | 2 | 3 | 3 | 100.00 | 1.33 | 5.00 | 0.44 | 1.67 | 3.75 | 0.44 |
| IR62 | 35 | 2 | 4 | 3 | 100.00 | 2.00 | 16.67 | 0.67 | 5.56 | 8.33 | 0.40 |
| IR62 | 35 | 3 | 1 | 3 | 66.67 | 0.67 | 0.00 | 0.33 | 0.00 | 0.00 | 0.24 |
| IR62 | 35 | 3 | 2 | 3 | 66.67 | 0.67 | 0.00 | 0.33 | 0.00 | 0.00 | 0.15 |
| IR62 | 35 | 3 | 3 | 3 | 66.67 | 0.67 | 0.00 | 0.33 | 0.00 | 0.00 | 0.24 |
| IR62 | 35 | 3 | 4 | 3 | 66.67 | 0.67 | 0.00 | 0.33 | 0.00 | 0.00 | 0.32 |
| IR62 | 35 | 4 | 1 | 3 | 44.44 | 0.78 | 0.67 |  |  |  | 0.23 |
| IR62 | 35 | 4 | 2 | 3 | 0.00 | 0.00 | 0.00 |  |  |  |  |
| IR62 | 35 | 4 | 3 | 3 | 66.67 | 1.00 | 0.67 | 0.50 | 0.33 | 0.67 | 0.47 |
| IR62 | 35 | 4 | 4 | 3 | 66.67 | 1.33 | 1.33 | 0.67 | 0.67 | 1.00 | 0.27 |
| IR62 | 35 | 5 | 1 | 3 | 22.22 | 0.22 | 0.00 |  |  |  | 0.12 |
| IR62 | 35 | 5 | 2 | 3 | 0.00 | 0.00 | 0.00 |  |  |  |  |
| IR62 | 35 | 5 | 3 | 3 | 33.33 | 0.33 | 0.00 | 0.33 | 0.00 | 0.00 | 0.19 |
| IR62 | 35 | 5 | 4 | 3 | 33.33 | 0.33 | 0.00 | 0.33 | 0.00 | 0.00 | 0.17 |
| IR62 | 35 | 6 | 1 | 3 | 22.22 | 0.22 | 0.00 |  |  |  | 0.94 |
| IR62 | 35 | 6 | 2 | 3 | 0.00 | 0.00 | 0.00 |  |  |  |  |
| IR62 | 35 | 6 | 3 | 3 | 33.33 | 0.33 | 0.00 | 0.33 | 0.00 | 0.00 | 0.13 |
| IR62 | 35 | 6 | 4 | 3 | 33.33 | 0.33 | 0.00 | 0.33 | 0.00 | 0.00 | 0.15 |
| IR62 | 35 | 7 | 1 | 3 | 0.00 | 0.00 | 0.00 |  |  |  |  |
| IR62 | 35 | 7 | 2 | 3 | 0.00 | 0.00 | 0.00 |  |  |  |  |
| IR62 | 35 | 7 | 3 | 3 | 0.00 | 0.00 | 0.00 |  |  |  |  |
| IR62 | 35 | 7 | 4 | 3 | 0.00 | 0.00 | 0.00 |  |  |  |  |
| IR62 | 35 | 8 | 1 | 3 | 0.00 | 0.00 | 0.00 |  |  |  |  |
| IR62 | 35 | 8 | 2 | 3 | 0.00 | 0.00 | 0.00 |  |  |  |  |
| IR62 | 35 | 8 | 3 | 3 | 0.00 | 0.00 | 0.00 |  |  |  |  |
| IR62 | 35 | 8 | 4 | 3 | 0.00 | 0.00 | 0.00 |  |  |  |  |
| IR62 | 35 | 9 | 1 | 3 | 0.00 | 0.00 | 0.00 |  |  |  |  |
| IR62 | 35 | 9 | 2 | 3 | 0.00 | 0.00 | 0.00 |  |  |  |  |
| IR62 | 35 | 9 | 3 | 3 | 0.00 | 0.00 | 0.00 |  |  |  |  |
| IR62 | 35 | 9 | 4 | 3 | 0.00 | 0.00 | 0.00 |  |  |  |  |
| IR62 | 35 | 10 | 1 | 3 | 0.00 | 0.00 | 0.00 |  |  |  |  |
| IR62 | 35 | 10 | 2 | 3 | 0.00 | 0.00 | 0.00 |  |  |  |  |
| IR62 | 35 | 10 | 3 | 3 | 0.00 | 0.00 | 0.00 |  |  |  |  |
| IR62 | 35 | 10 | 4 | 3 | 0.00 | 0.00 | 0.00 |  |  |  |  |
| IR62 | 35 | 11 | 1 | 3 | 0.00 | 0.00 | 0.00 |  |  |  |  |
| IR62 | 35 | 11 | 2 | 3 | 0.00 | 0.00 | 0.00 |  |  |  |  |
| IR62 | 35 | 11 | 3 | 3 | 0.00 | 0.00 | 0.00 |  |  |  |  |
| IR62 | 35 | 11 | 4 | 3 | 0.00 | 0.00 | 0.00 |  |  |  |  |
| IR62 | 35 | 12 | 1 | 3 | 0.00 | 0.00 | 0.00 |  |  |  |  |
| IR62 | 35 | 12 | 2 | 3 | 0.00 | 0.00 | 0.00 |  |  |  |  |
| IR62 | 35 | 12 | 3 | 3 | 0.00 | 0.00 | 0.00 |  |  |  |  |
| IR62 | 35 | 12 | 4 | 3 | 0.00 | 0.00 | 0.00 |  |  |  |  |
| IR62 | 35 | 13 | 1 | 3 | 0.00 | 0.00 | 0.00 |  |  |  |  |
| IR62 | 35 | 13 | 2 | 3 | 0.00 | 0.00 | 0.00 |  |  |  |  |
| IR62 | 35 | 13 | 3 | 3 | 0.00 | 0.00 | 0.00 |  |  |  |  |
| IR62 | 35 | 13 | 4 | 3 | 0.00 | 0.00 | 0.00 |  |  |  |  |
| IR62 | 35 | 14 | 1 | 3 | 0.00 | 0.00 | 0.00 |  |  |  |  |
| IR62 | 35 | 14 | 2 | 3 | 0.00 | 0.00 | 0.00 |  |  |  |  |
| IR62 | 35 | 14 | 3 | 3 | 0.00 | 0.00 | 0.00 |  |  |  |  |
| IR62 | 35 | 14 | 4 | 3 | 0.00 | 0.00 | 0.00 |  |  |  |  |
| IR62 | 35 | 15 | 1 | 3 | 0.00 | 0.00 | 0.00 |  |  |  |  |
| IR62 | 35 | 15 | 2 | 3 | 0.00 | 0.00 | 0.00 |  |  |  |  |
| IR62 | 35 | 15 | 3 | 3 | 0.00 | 0.00 | 0.00 |  |  |  |  |
| IR62 | 35 | 15 | 4 | 3 | 0.00 | 0.00 | 0.00 |  |  |  |  |
| IR62 | 35 | 16 | 1 | 3 | 0.00 | 0.00 | 0.00 |  |  |  |  |
| IR62 | 35 | 16 | 2 | 3 | 0.00 | 0.00 | 0.00 |  |  |  |  |
| IR62 | 35 | 16 | 3 | 3 | 0.00 | 0.00 | 0.00 |  |  |  |  |
| IR62 | 35 | 16 | 4 | 3 | 0.00 | 0.00 | 0.00 |  |  |  |  |
| IR62 | 35 | 17 | 1 | 3 | 0.00 | 0.00 | 0.00 |  |  |  |  |
| IR62 | 35 | 17 | 2 | 3 | 0.00 | 0.00 | 0.00 |  |  |  |  |
| IR62 | 35 | 17 | 3 | 3 | 0.00 | 0.00 | 0.00 |  |  |  |  |
| IR62 | 35 | 17 | 4 | 3 | 0.00 | 0.00 | 0.00 |  |  |  |  |
| IR62 | 35 | 18 | 1 | 3 | 0.00 | 0.00 | 0.00 |  |  |  |  |
| IR62 | 35 | 18 | 2 | 3 | 0.00 | 0.00 | 0.00 |  |  |  |  |
| IR62 | 35 | 18 | 3 | 3 | 0.00 | 0.00 | 0.00 |  |  |  |  |
| IR62 | 35 | 18 | 4 | 3 | 0.00 | 0.00 | 0.00 |  |  |  |  |
| IR62 | 35 | 19 | 1 | 3 | 0.00 | 0.00 | 0.00 |  |  |  |  |
| IR62 | 35 | 19 | 2 | 3 | 0.00 | 0.00 | 0.00 |  |  |  |  |
| IR62 | 35 | 19 | 3 | 3 | 0.00 | 0.00 | 0.00 |  |  |  |  |
| IR62 | 35 | 19 | 4 | 3 | 0.00 | 0.00 | 0.00 |  |  |  |  |
| IR62 | 35 | 20 | 1 | 3 | 0.00 | 0.00 | 0.00 |  |  |  |  |
| IR62 | 35 | 20 | 2 | 3 | 0.00 | 0.00 | 0.00 |  |  |  |  |
| IR62 | 35 | 20 | 3 | 3 | 0.00 | 0.00 | 0.00 |  |  |  |  |
| IR62 | 35 | 20 | 4 | 3 | 0.00 | 0.00 | 0.00 |  |  |  |  |
